# In Situ Inhibitor Synthesis and Screening by Fluorescence Polarization: An Efficient Approach for Accelerating Drug Discovery

**DOI:** 10.1002/anie.202211510

**Published:** 2022-10-11

**Authors:** Zhihong Li, Yue Wu, Shuai Zhen, Kaijun Su, Linjian Zhang, Fulai Yang, Michael A. McDonough, Christopher J. Schofield, Xiaojin Zhang

**Affiliations:** ^1^ State Key Laboratory of Natural Medicines Jiangsu Key Laboratory of Drug Design and Optimization, and Department of Chemistry China Pharmaceutical University Nanjing 211198 China; ^2^ Chemistry Research Laboratory and the Ineos Oxford Institute for Antimicrobial Research University of Oxford 12 Mansfield Road Oxford OX1 3TA UK

**Keywords:** Drug Discovery, Fluorescence Polarization, Hypoxia, In Situ Inhibitor Synthesis and Screening, PHD2

## Abstract

Target‐directed dynamic combinatorial chemistry has emerged as a useful tool for hit identification, but has not been widely used, in part due to challenges associated with analyses involving complex mixtures. We describe an operationally simple alternative: in situ inhibitor synthesis and screening (ISISS), which links high‐throughput bioorthogonal synthesis with screening for target binding by fluorescence. We exemplify the ISISS method by showing how coupling screening for target binding by fluorescence polarization with the reaction of acyl‐hydrazides and aldehydes led to the efficient discovery of a potent and novel acylhydrazone‐based inhibitor of human prolyl hydroxylase 2 (PHD2), a target for anemia treatment, with equivalent in vivo potency to an approved medicine.

## Graphical abstract

In situ inhibitor synthesis and screening (ISISS) links high‐throughput bioorthogonal synthesis with screening tor target binding by fluorescence, as exemplified by the efficient identification of in vivo orally active inhibitors of PHD2, a target for anemia treatment.

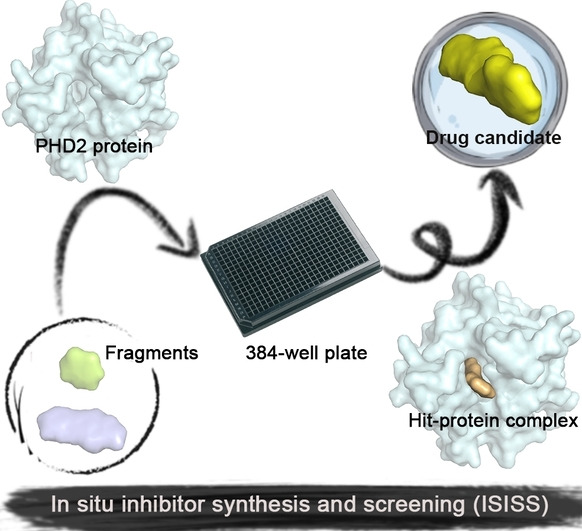

The efficient discovery of small‐molecule modulators of biomacromolecule function remains a limiting step in target validation and drug discovery.^[1]^ In the conventional procedure for hit compound discovery, iterative cycles of “design‐synthesis‐screen” are carried out until a potent candidate for further research is obtained. Target‐directed dynamic combinatorial chemistry (tdDCC) has been developed as an alternative method for hit identification.^[2]^ tdDCC involves the target‐based selection of hits from dynamic mixtures of reversibly reacting compounds (Figure S1).^[3]^ tdDCC can enable the spatially resolved identification of compounds that bind strongly to a defined target protein.^[4]^

The utility of tdDCC is, however, dependent on methods for real‐time monitoring of dynamically interchanging mixtures, which can be challenging. Assay methods for tdDCC, including high‐performance liquid chromatography (HPLC),[^3c^, ^5^] mass spectrometry (MS),^[6]^ and nuclear magnetic resonance (NMR),^[7]^ have been developed. In general, however, these assays are limited to small dynamic combinatorial library (DCL) sizes, employ a relatively large amount of protein (>10 μM) and are operationally cumbersome.

An approach to identify protease inhibitors involving the reversible in situ reaction of aldehydes and nucleophiles monitoring inhibition of hydrolysis of a fluorogenic reporter substrate has been reported.^[8]^ A fluorescence polarization (FP) assay has been applied in combination with fragment ligation to optimize protein binding: a fluorescein‐labeled substrate analog peptide with a C‐terminal aldehyde was extended by in situ reaction with nucleophilic fragments to enhance protein binding affinity.^[9]^ Here, we report how in situ synthesis and screening of inhibitors in individual wells (ISISS) enabled the efficient discovery of inhibitors of a human enzyme suitable for in vivo use. The ISISS method couples a biorthogonal reaction with an FP‐based target binding assay and enables time‐independent detection of a large variety of fragment combinations. The ISISS method is operationally simple and can be conducted in a 384‐well plate high‐throughput format (Figure 1).

**Figure 1:**
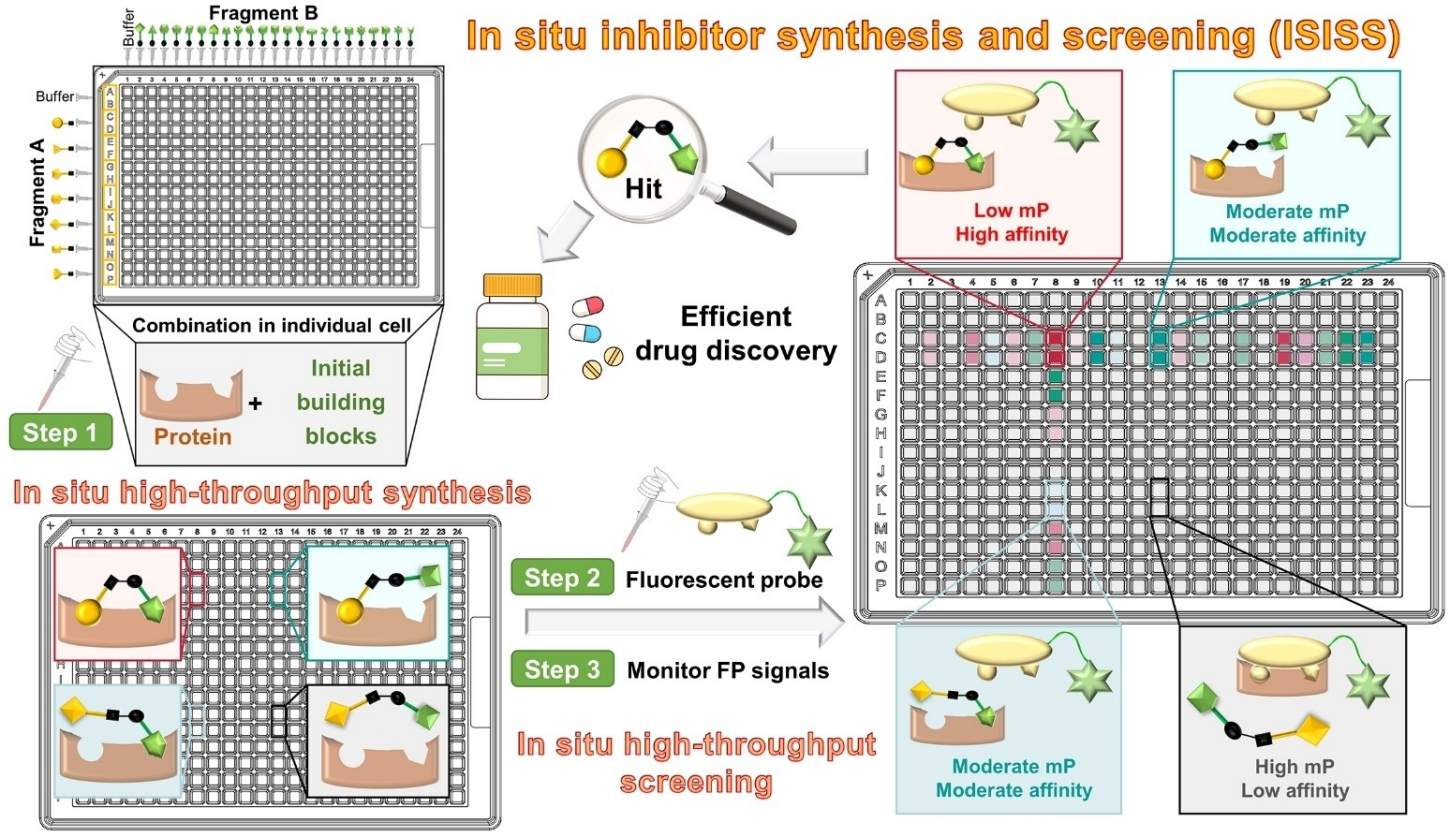
Overview of principles and procedures for in situ inhibitor synthesis and screening (ISISS). mP: millipolarization.

We applied the FP‐based ISISS strategy to discovery of in vivo active inhibitors of human prolyl hydroxylase 2 (PHD2), a target for treatment of chronic kidney disease (CKD) related anemia. The ISISS method employs a fluorescein‐labeled probe, which was prepared by linking fluorescein isothiocyanate (FITC) and a potent PHD2 inhibitor (the probe structure is shown in Figure S2) and which monitors binding of competitive ligands using a low concentration of human PHD2 (20 nM) via FP analysis (Figure S2).^[10]^ PHD catalysis negatively regulates biosynthesis of erythropoietin, hence PHD inhibitors promote hemoglobin (Hb) production and erythropoiesis.^[11]^ PHD2 inhibitors have the potential to revolutionize the treatment of anemia, with the first‐in‐class PHD2 inhibitor, Roxadustat, now approved for clinical use.^[12]^ Here we report how the ISISS method efficiently enabled identification of PHD2 inhibitors with similar potency to Roxadustat, including in an in vivo context.

Informed by the structural features of the PHD2 active site (Figure 2A) and biorthogonal acylhydrazone formation reaction,^[13]^ we identified potential building blocks for ISISS to identify PHD inhibitors, comprising hydrazides **A1**–**A5** (Figure 2A) and commercially available aldehydes **B1**–**B90** (Figure S3). Docking studies suggested that a potent PHD2 inhibitor might be obtained by acylhydrazone formation (Figure S4): Fragments **A** could occupy the cosubstrate (2‐oxoglutarate, 2OG) binding site (pocket 1), while fragments **B** could occupy the substrate (hypoxia inducible factor‐α, HIF‐α) binding site (pocket 2) (Figure 2A). FP assays showed that **A1**–**A5** manifest weak binding to PHD2 at 1 μM, while none of the aldehyde fragments **B** (3 μM) bound to PHD2 (Figure S5).

**Figure 2:**
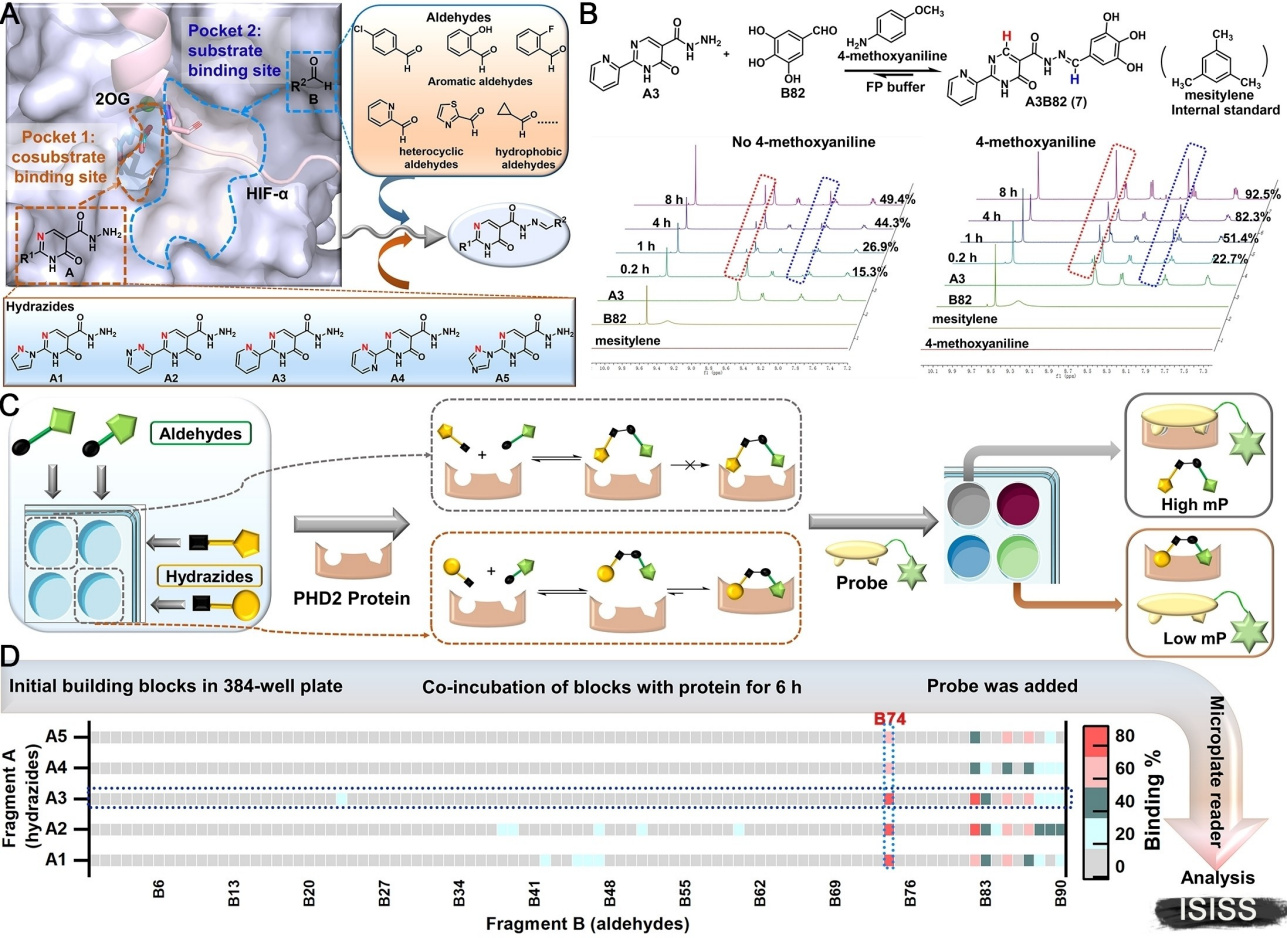
A) Validation of ISISS based on PHD2 structural features including substrate binding mode (PDB ID: 5 L9B),^[11a]^ employing acyl‐hydrazide derivatives and aldehydes. B) Real‐time ^1^H NMR analysis of the reaction of **A3** (50 mM) and **B82** (150 mM) in FP buffer (50 μL D_2_O and 450 μL DMSO‐*d*_6_). C) Schematic of FP‐based ISISS. D) Heat map of ISISS involving hydrazide **A** (1 μM) and aldehyde **B** (3 μM) components. gray: 0 %‐16 %, cyan: 17 %‐35 %, dark green: 36 %‐54 %, light red: 55 %‐72 %, red: 73 %‐92 % binding.

The conditions for ISISS with PHD2 were then investigated by ^1^H NMR and FP assays. ^1^H NMR experiments were conducted to monitor acylhydrazone formation in real time and the stability of the products/reversibility of their formation. The FP assays showed good compatibility with 4‐methoxyaniline (Figure S6), the presence of which substantially improves the efficiency of acylhydrazone exchange (Figure 2B). We then performed ISISS in a 384‐well plate by reacting hydrazides (**A1**–**A5**) and aldehydes (**B1**–**B90**) with 4‐methoxyaniline in an FP buffer containing PHD2. The FP probe was added after mixing the acyl hydrazine and aldehyde components (6 h); the FP signal was recorded when the polarization value (mP) was stable as observed using a microplate reader. (Figure 2C). The binding affinities were calculated by measuring changes in the mP.[^8^, ^12^] Heat map analysis of the ISISS output compounds (Figure 2D) reveals that compounds with phenolic hydroxyl groups derived from the aldehyde component of the reactions exhibit excellent affinity for PHD2, especially those from the 2,3‐dihydroxy aldehyde fragment (**B74**).

Compounds **1**–**10** were selected for validation by synthesis, considering both their structural diversity and potency. The affinities of purified **1**–**10** for PHD2 were evaluated by the FP assay; the results exhibited a similar trend to that from ISISS. The most potent scaffolds **2** (**A2B74**, *K*_i_=0.068±0.006 μM) and **3** (**A3B74**, *K*_i_=0.048±0.004 μM) manifest a comparable PHD2 binding affinity compared to Roxadustat (*K*_i_=0.073±0.010 μM). Molecular modeling of **2** and **3** indicated that the nitrogen atoms of fragment **A** could form a 5‐membered chelate ring with the active site ferrous iron (Figure 3B). In the model, the carbonyl group of the pyrimidine ring is positioned to H‐bond with Arg252, while the oxygen of acylhydrazone is positioned to form an H‐bond with Tyr310. Notably, the phenolic hydroxyls are positioned to form H‐bonds with Val241 and Ser242 (Figure 3C/D), respectively (the phenyl ring extends towards solvent). Although we only considered binding of, and only draw, the thermodynamically most stable *E/trans* form of the acyl‐hydrazones,^[15]^ we cannot exclude the possibility that, at least in some cases, binding of the *Z/cis* form is relevant.

**Figure 3:**
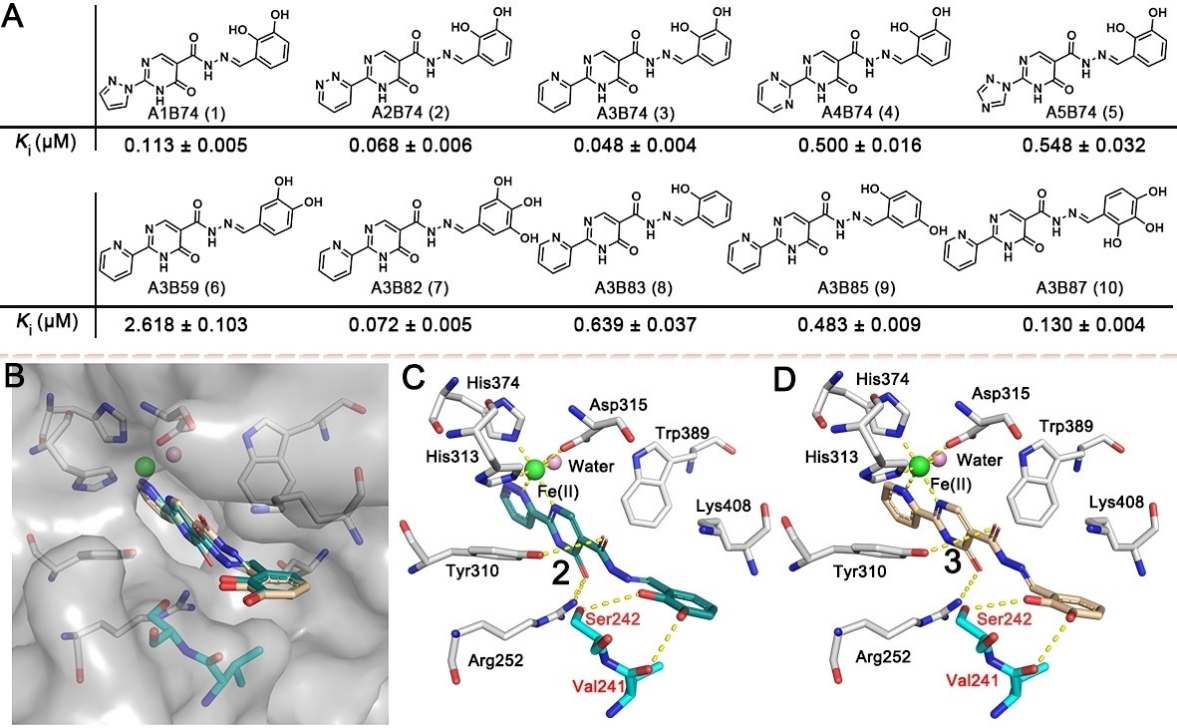
A) Acylhydrazone scaffolds (**1**–**10**) identified by ISISS and their binding affinities for PHD2, mean±SD. B, C) Proposed binding modes of **2** (blackish green) and B, D) **3** (wheat) with PHD2 (PDB ID: 4KBZ);^[16]^ hydrogen bonds are represented as yellow dashed lines.

The dihydroxy/catechol fragment has been identified as having potential to cause toxicity.^[17]^ Hence, based on the binding modes of **2** and **3**, we performed further ISISS studies using the components with halogen, carboxyl, and other groups (Figure S3, **B91**–**B102**) aiming to replace the dihydroxy moiety, thereby improving the druggability of the outputs. Scatter plots of the screening results are shown in Figure 4A. Tight binding acylhydrazone derivatives (**11**–**18**) were identified; most of them exhibited affinities at the nanomolar level for PHD2. Notably, **17**, an isomer of **16**, differing only in the positions of the phenylhydroxyl and phenylchloro groups showed substantially more potent PHD2 inhibition with an apparent *K*_i_ value of 0.027±0.002 μM compared to 0.394±0.017 μM for **16** (Figure 4B). Docking of **17** with the PHD2 active site implies that the *ortho* chloro‐group of **17** can form an H‐bond with Ser242, while the *meta* hydroxyl group can H‐bond with Val241 (Figure 4C).

**Figure 4:**
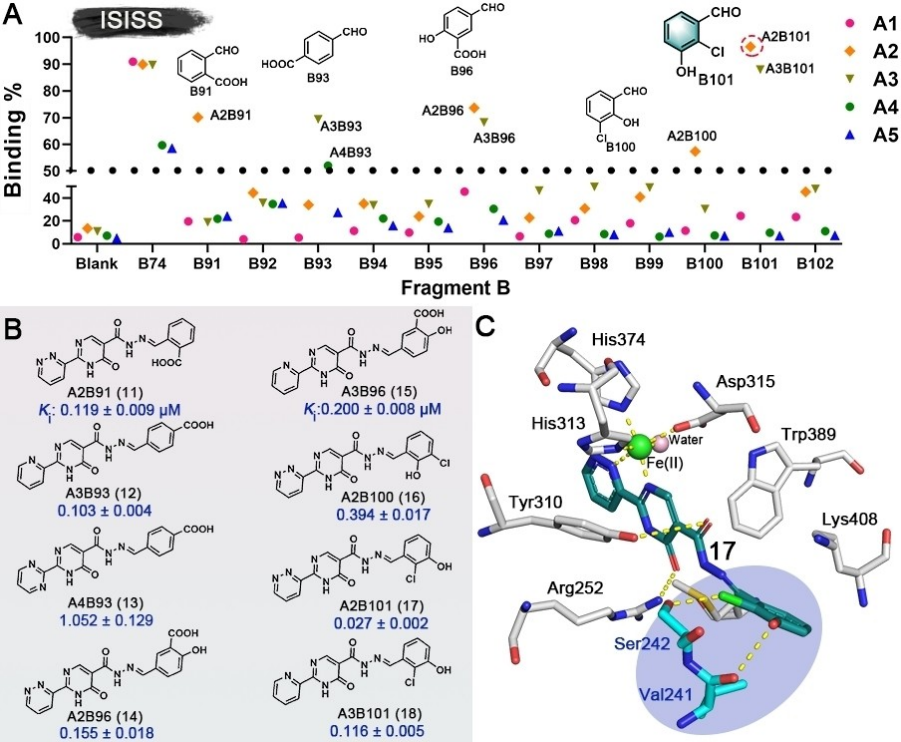
A) Scatter plot of inhibition of ISISS PHD2 binders. B) Acylhydrazone scaffolds (**11**–**18**) and their binding affinities for PHD2, mean±SD. C) Proposed binding mode of **17** with PHD2 (based on PDB ID: 4KBZ),^[16]^ hydrogen bonds: yellow dashed lines.

The cytotoxicities of **1**–**18** were evaluated in hepatocellular carcinoma Hep3B cells; none of them exhibited obvious cytotoxicity (Figure S7). RT‐qPCR assays were performed to investigate upregulation of the HIF‐associated erythropoietin (EPO) gene by the best obtained ISISS binder (**17**). Importantly, the results show that **17** increases *EPO* mRNA in a concentration‐dependent manner with comparable efficiency to Roxadustat (Figure 5A). Metabolic stability assays showed >80 % of administered **17** was present (t_1/2_>100 min) after 50 min in rat, mouse, and human liver microsomes; **17** was also stable in rat plasma (Figure S8).

**Figure 5:**
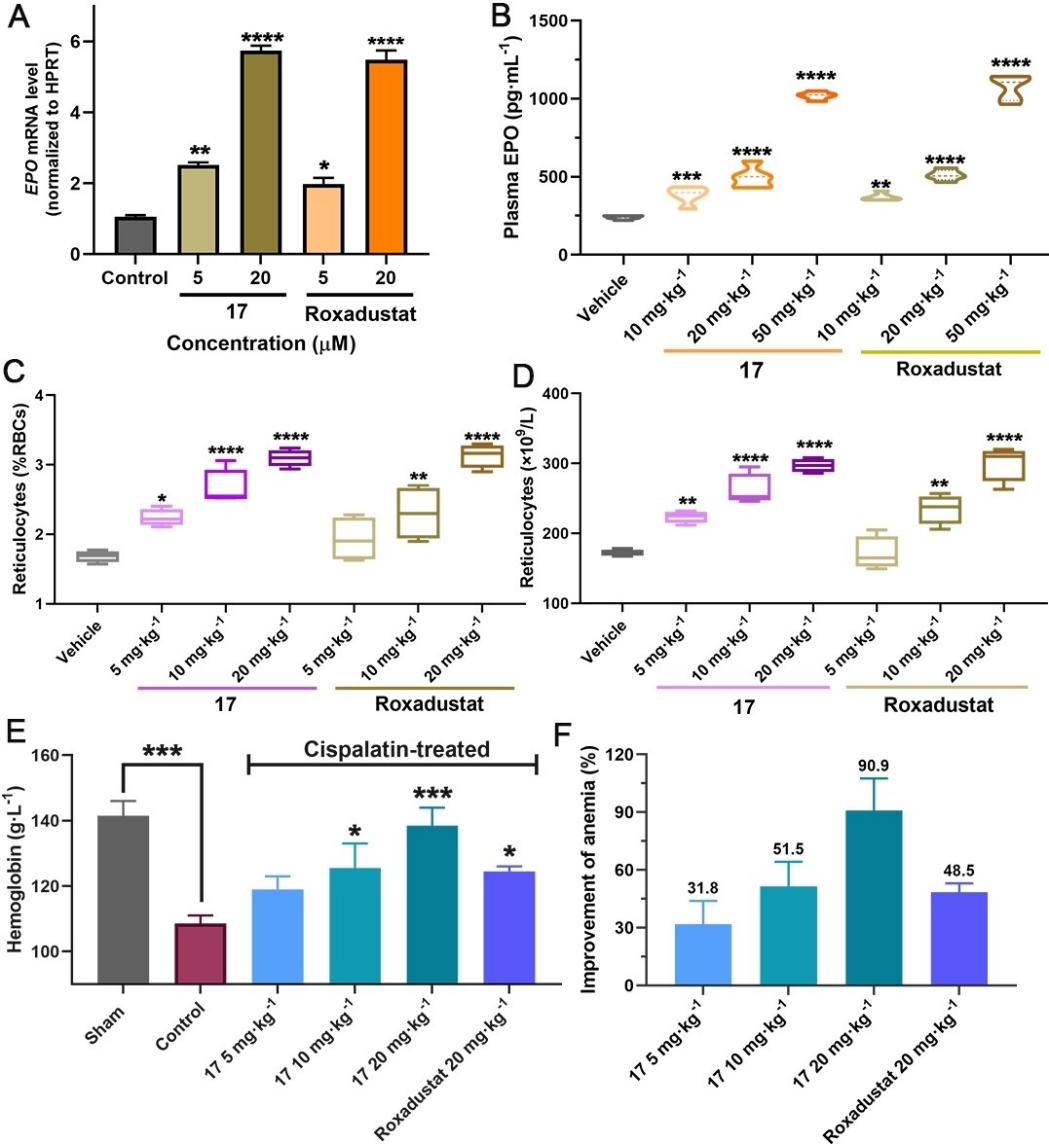
A) Expression of *EPO* mRNA by RT‐qPCR after treatment with **17** in Hep3B cells (reference gene: *HPRT*). B) **17** and Roxadustat (10, 20, and 50 mg kg^−1^) mediated plasma EPO response in mice (C57BL/6 J) with *po* administration. C, D) **17** and Roxadustat (5, 10, and 20 mg kg^−1^) mediated changes in reticulocytes in mice with *po* administration. E) Changes in Hb after orally administrated **17** or Roxadustat in C57BL/6 J mouse models of anemia induced by cisplatin. F) Anti‐anemia activities of **17** and Roxadustat in mouse models of anemia induced by cisplatin. Mean±SEM. P values were analyzed by two‐way ANOVA comparing with the vehicle/sham/control group (*, P<0.05; **, P<0.01; ***, P<0.001; ****, P<0.0001).

Encouraged by the potency of PHD2 inhibition in vitro, eight representative ISISS compounds (20 mg kg^−1^) were selected for treating C57BL/6 J male mice via po administration, initially measuring reticulocyte levels to assess activity. The results indicated that **17** was superior with respect to inducing reticulocytes compared to the other tested ISISS compounds, performing as well as, or slightly better than, Roxadustat (Figure S9). To further investigate its in vivo efficacy, multiple doses of **17** and Roxadustat were evaluated for their ability to stimulate reticulocytes and EPO production in C57BL/6 J mice (Figure 5B). Plasma EPO analyses show that **17** causes dose‐dependent induction of EPO, similarly to Roxadustat. A dose‐dependent increase in reticulocytes was observed in the **17**‐treated group. **17** exhibited similar or better pharmacodynamics compared to Roxadustat (Figure 5C/D). The anti‐anemia efficacy of **17** was then investigated in cisplatin‐induced anemia mice; Figure 5E, the hemoglobin (Hb) levels of **17**‐treated mice were increased in a dose‐dependent manner by po administered **17**, indicating that **17** can upregulate EPO and stimulate erythropoiesis production, in a manner useful for the treatment of anemia (Figure 5F).

To further evaluate the in vivo safety profiles of **17**, both subacute (10, 50, and 100 mg kg^−1^) and acute (400 mg kg^−1^) oral toxicity studies were carried out in ICR mice. These revealed no obvious abnormality after treatment with **17**: there were no significant differences in the body weight, organ/body weight ratio, blood biochemistry indexes (alanine aminotransferase, ALT; aspartate aminotransferase, AST; blood urea nitrogen, BUN; and creatinine, CRE) between the treated groups and vehicle groups (Figure S10/11). Hematoxylin and eosin (H&E) staining for major organs demonstrated that the **17**‐treated group (100 mg kg^−1^) exhibited normal architecture comparable to the vehicle (Figure S11). These results imply that **17** has an acceptable in vivo safety profile for further clinical development.

The overall results reveal how ISISS, which integrates in situ synthesis and FP‐based screening can enable the efficient discovery of novel binders/inhibitors of a pharmacologically challenging target. Importantly, ISISS enabled the rapid identification of compounds that tightly bind to PHD2 that are suitable for in vivo use. Thus, acylhydrazone derivative **17** is a potent PHD2 inhibitor manifesting anti‐anemia activity in a cisplatin‐induced mouse model with an excellent in vivo safety profile. There is scope for variations on the ISISS technology exemplified here, i.e. by using different synthesis reactions and screening methods. In this regard it is notable that our ISISS work was driven by an assay measuring binding to PHD2 rather than inhibition of turnover. Further, although we characterized all the individual reaction products in the ISISS screen by MS and spectroscopy, this only needs be done for compounds that are progressed to the next stage. We anticipate that the ISISS methodology, which is suitable for use by those not expert in synthetic chemistry, will be applicable to many other targets.

## Conflict of interest

The authors declare no conflict of interest.

## Supporting information

As a service to our authors and readers, this journal provides supporting information supplied by the authors. Such materials are peer reviewed and may be re‐organized for online delivery, but are not copy‐edited or typeset. Technical support issues arising from supporting information (other than missing files) should be addressed to the authors.

Supporting Information

## Data Availability

The data that support the findings of this study are available in the supplementary material of this article.
