# Supplementary material for: In Situ Inhibitor Synthesis and Screening by Fluorescence Polarization: An Efficient Approach for Accelerating Drug Discovery
Source: Angew Chem Int Ed Engl. 2022 Oct 11;61(45):e202211510. doi: 10.1002/anie.202211510 (PMC9827864; doi:10.1002/anie.202211510)
Supplement: Supplementary file 1 — Supporting Information [file ANIE-61-0-s001.pdf]

Angewandte Chemie
Eine Zeitschrift der Gesellschaft Deutscher Chemiker

Supporting Information

In Situ Inhibitor Synthesis and Screening by Fluorescence Polarization: An Efficient Approach for Accelerating Drug Discovery

Z. Li, Y. Wu, S. Zhen, K. Su, L. Zhang, F. Yang, M. A. McDonough, C. J. Schofield, X. Zhang**

Contents

1. Supplementary figures S3

Figure S1. Schematic illustration of conventional procedure and tdDCC method. S3

Figure S2. Schematic of the FP assay for PHD2. ..... S4

Figure S3. Structures of commercially available aldehydes (**B1−B102**). S5

Figure S4. Docked binding modes of compounds and PHD2 ..... S6

Figure S5. Inhibitory % of **A1-A5** and **B1-B102** for PHD2. ..... S7

Figure S6. Evaluation of FP-based tdDCC screening conditions using FP assay. S8

Figure S7. Cytotoxicity of acylhydrazones (**1-18**) toward Hep3B cells. S9

Figure S8. Stability of compound **17** S10

Figure S9. Reticulocytes evaluation *in vivo* S11

Figure S10. Acute oral toxicity of tests for compound **17**. S12

Figure S11. Subacute oral toxicity of compound **17** S13

2. *In-vitro* evaluation S14

2.1 Fluorescence polarization (FP) competition assays withf PHD2 ..... S14

2.2 In situ inhibitor synthesis and screening (ISISS) assay S14

2.3 RT-qPCR assay S14

2.4 Cell viability assay S14

3. Molecular Modeling S15

4. Metabolic stability assay S15

5. Pharmacodynamic assay S15

6. Subacute and acute oral toxicity assay S16

7. NMR experiment S16

8. Synthesis S16

Ethical statement S21

^{1}H NMR, HR-MS, and ^{13}C NMR spectrums of **A1-A5** and the final products of **1-18**. S22

References S48

Author contribution S48

1. Supplementary figures

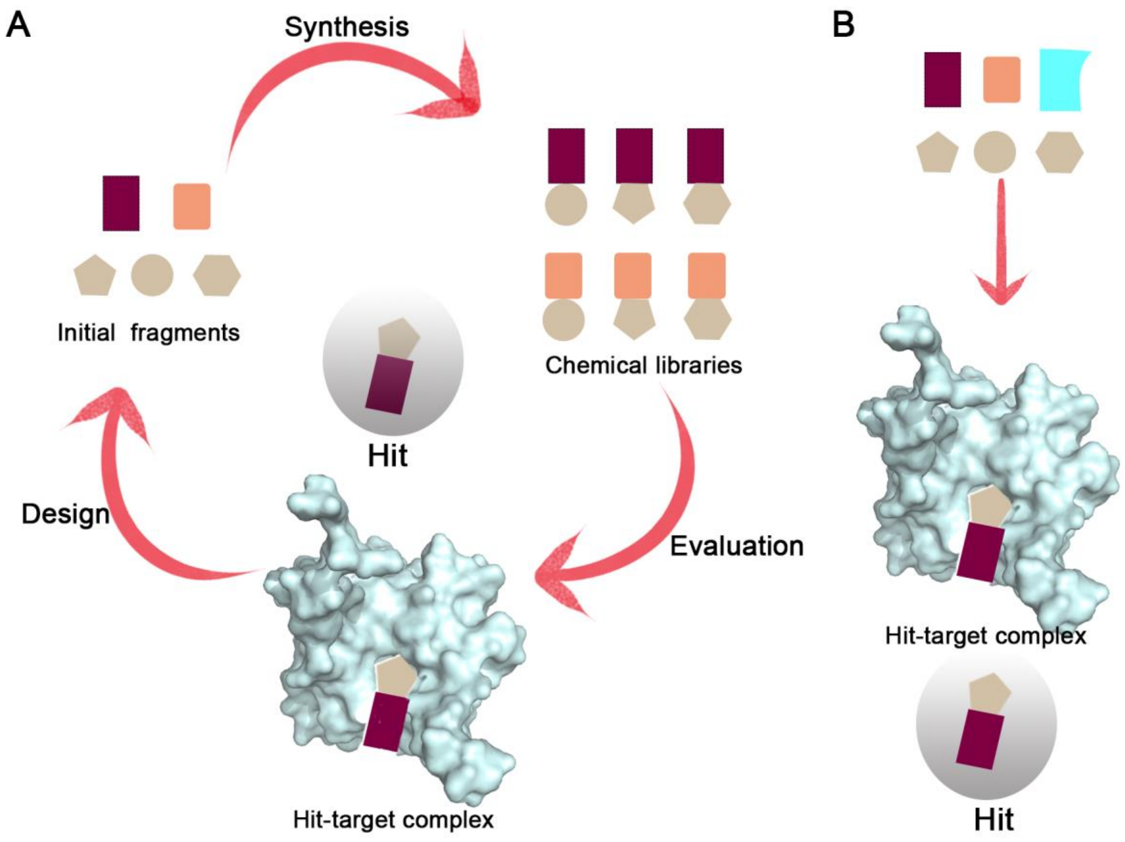

Figure S1 Schematic illustration of a conventional screening procedure (A) and target-directed dynamic combinatorial chemistry (tdDCC) (B).

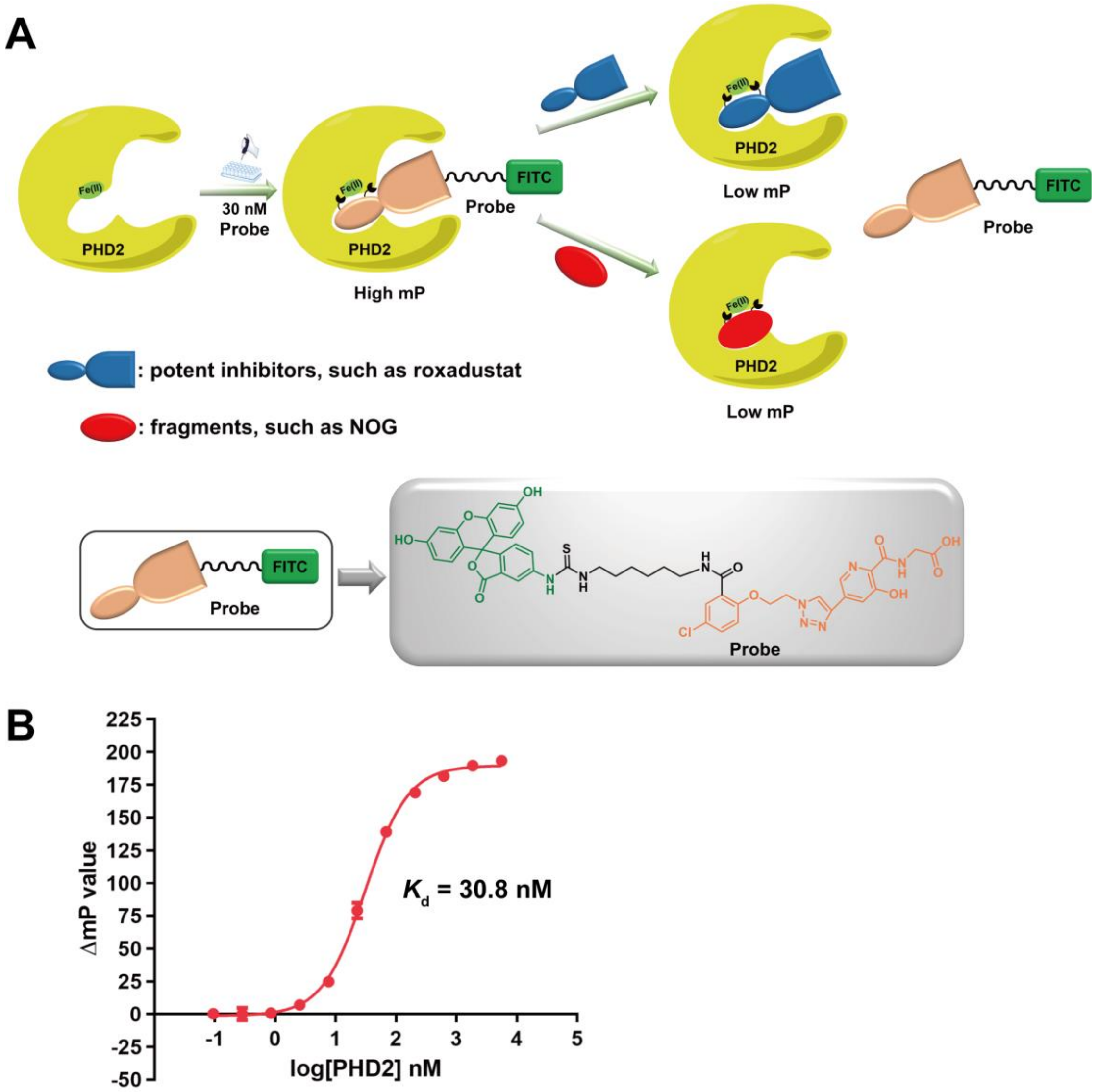

Figure S2 (A) Schematic of the FP assay for PHD2. FP is used to investigate binding of the probe and PHD2 and the displacement of the probe by PHD2 inhibitors (with both weak and strong affinities)[1]. mP: millipolarization. (B) The K_d value was determined using a constant concentration of probe and titrating with increasing concentrations of PHD2.

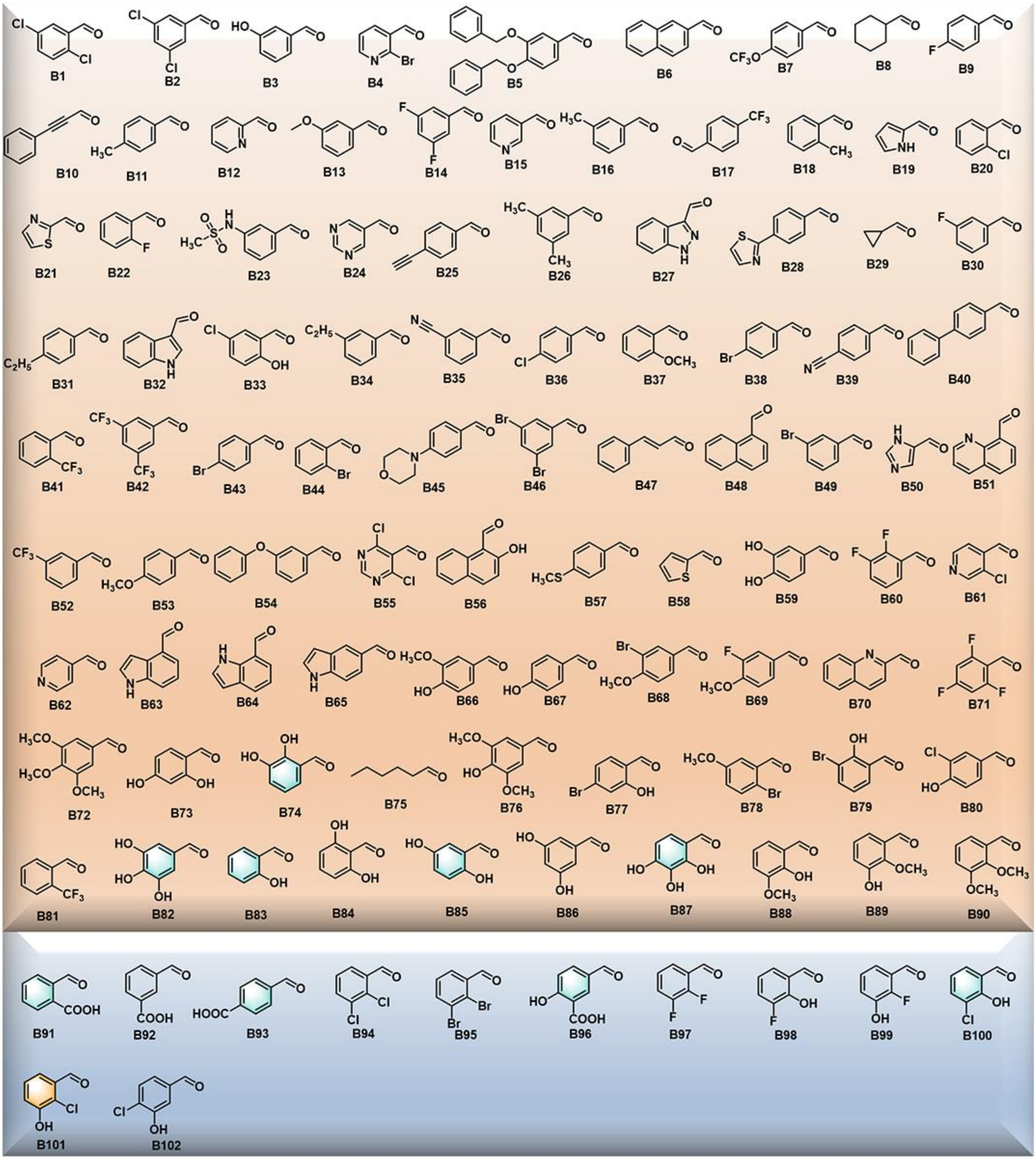

Figure S3. Structures of commercially available aldehydes used in our work (**B1−B102**).

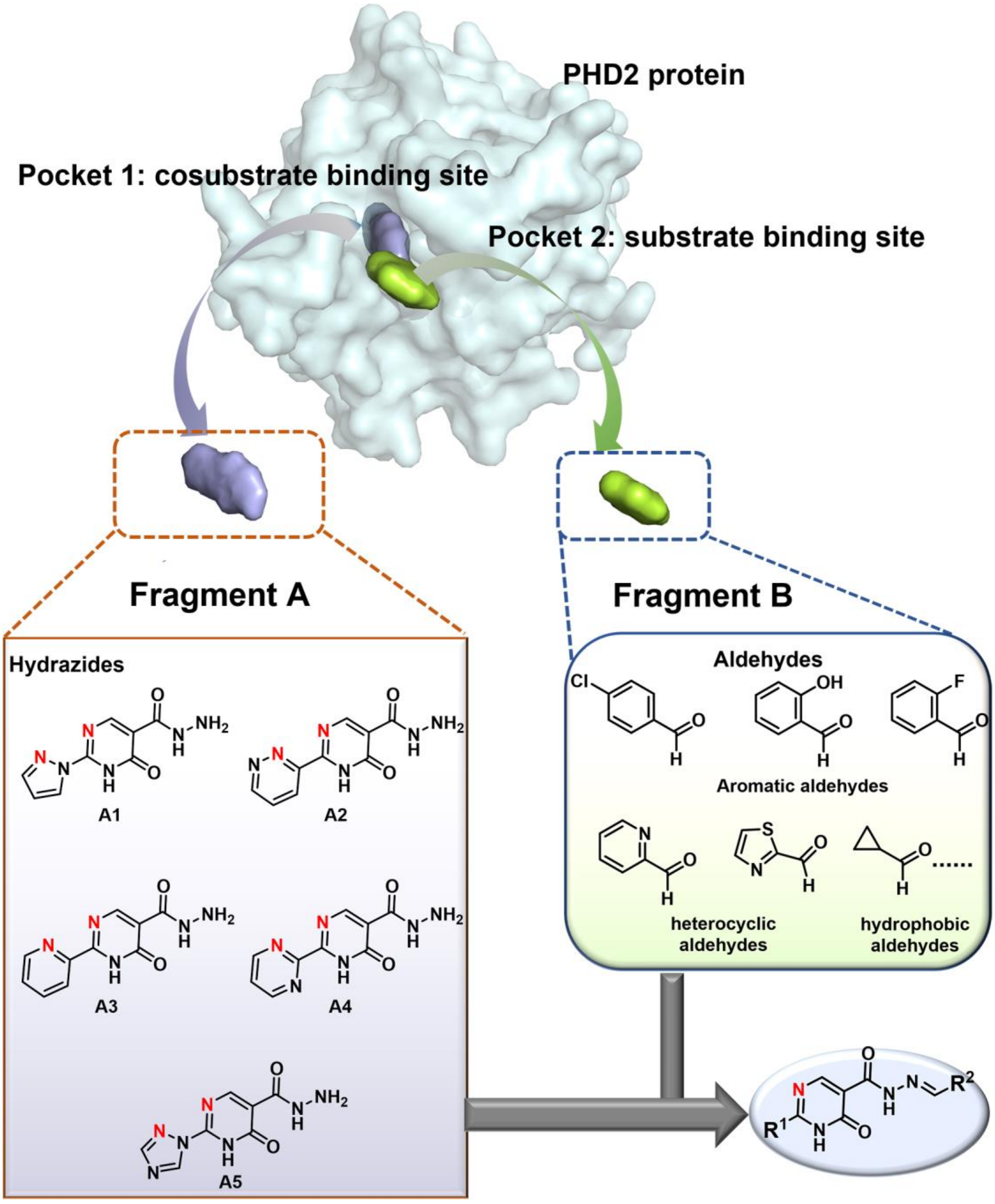

Figure S4 Docked binding modes of in situ synthesized / screened compounds at the active site of PHD2 (PDB ID: 4KBZ)[2].

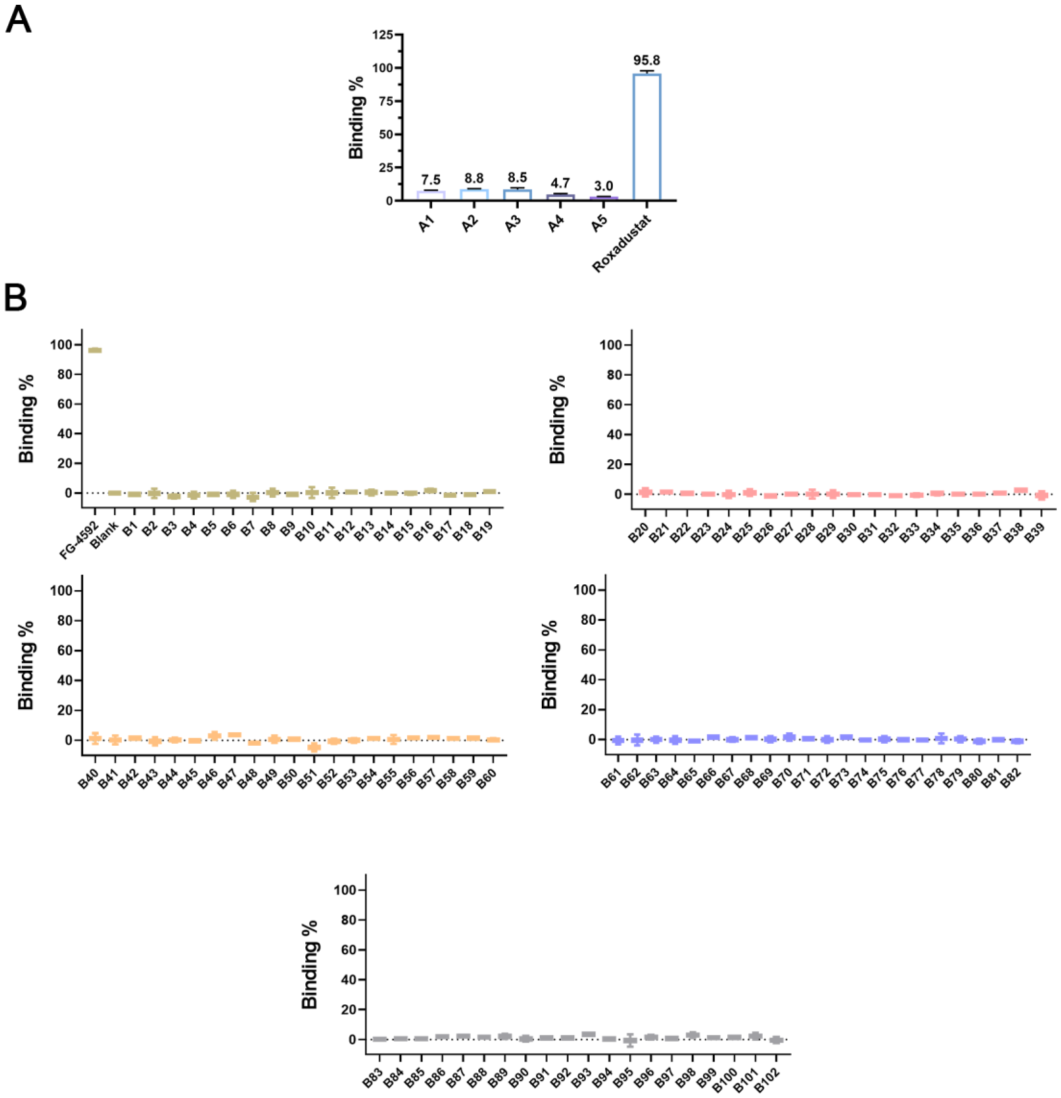

Figure S5. Inhibition of hydrazide derivatives (**A1−A5**, 1 µM) and **B1**-**B102** (3 µM) for PHD2. Errors: mean ± SD, n = 3.

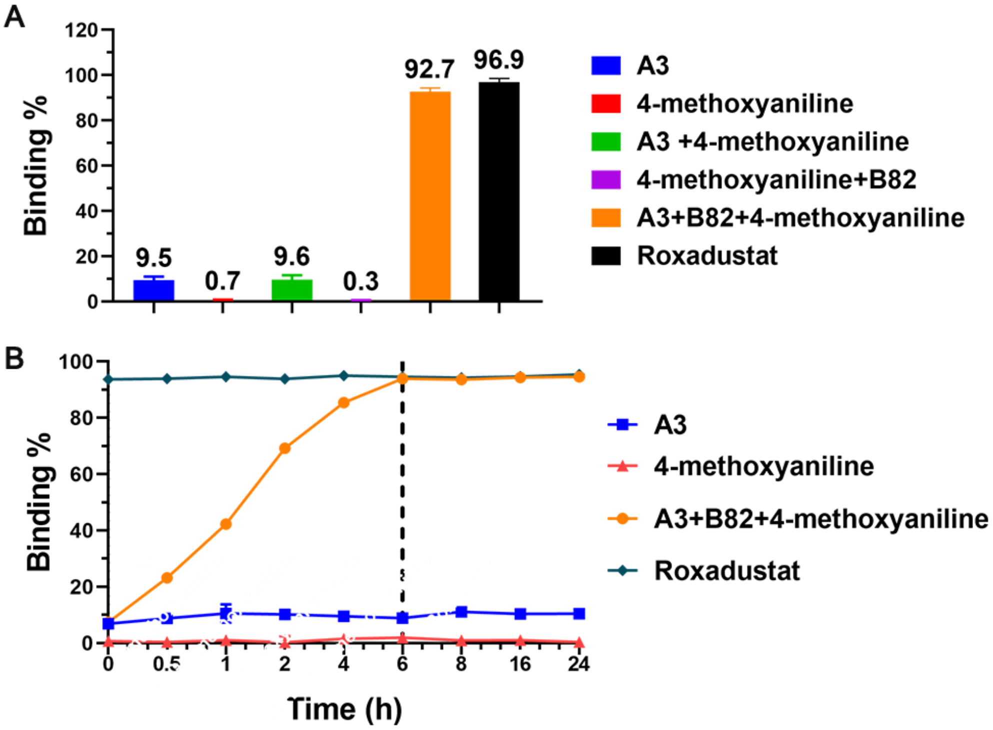

Figure S6. Evaluation of FP-based in situ screening conditions used in the ISISS assays. (A) PHD2 inhibition after 24 hours of co-incubation of the corresponding components. Errors: mean ± SD, n = 3. (B) Real-time monitoring of PHD2 inhibition of the corresponding components. Errors: mean ± SD, n = 3.

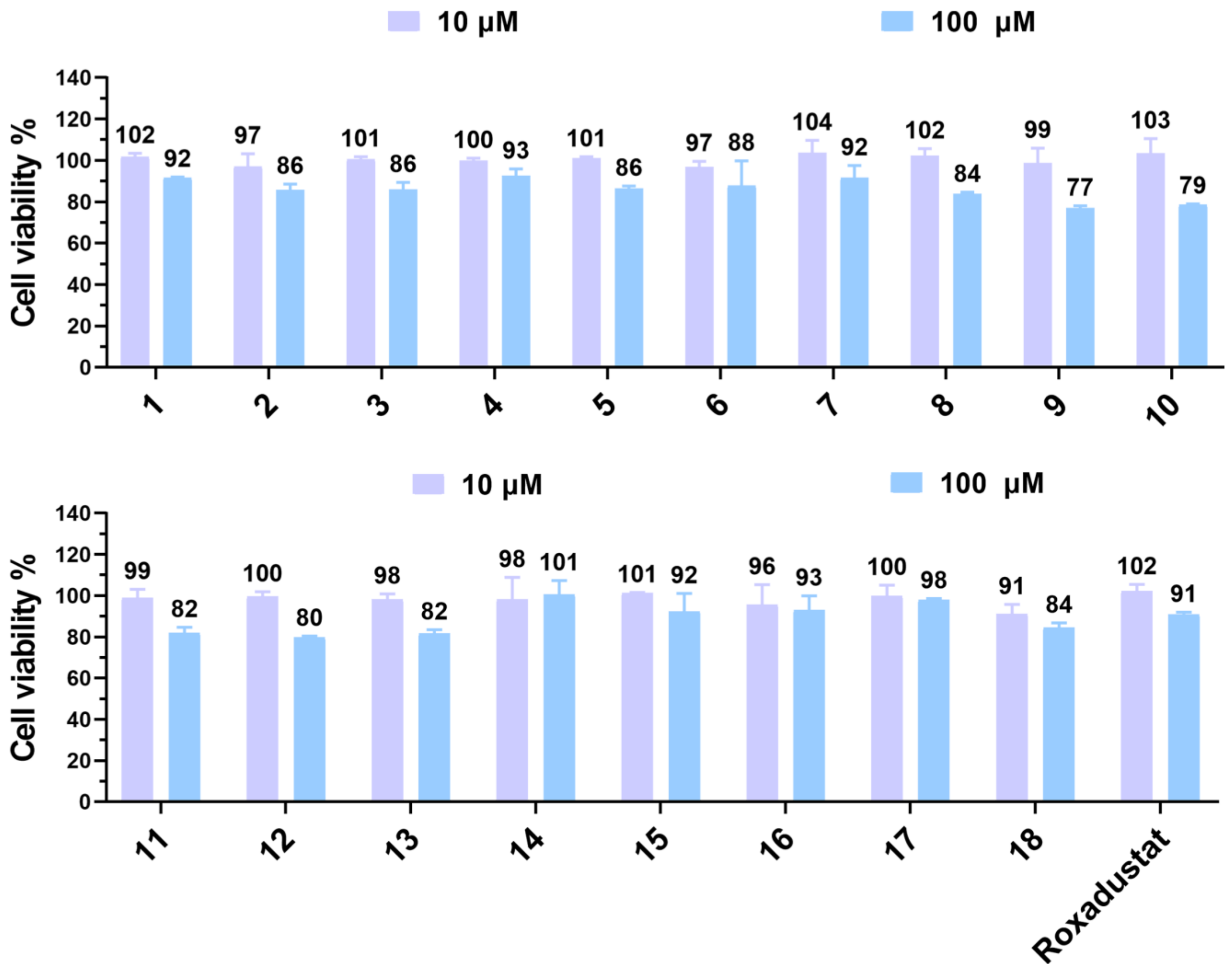

Figure S7. Cytotoxicity of acylhydrazones (**1**-**18**) towards Hep3B cells. The results indicate that the compounds have no significant cytotoxic activity at the tested concentrations (10 µM and 100 µM). Errors: mean ± SD, n = 3.

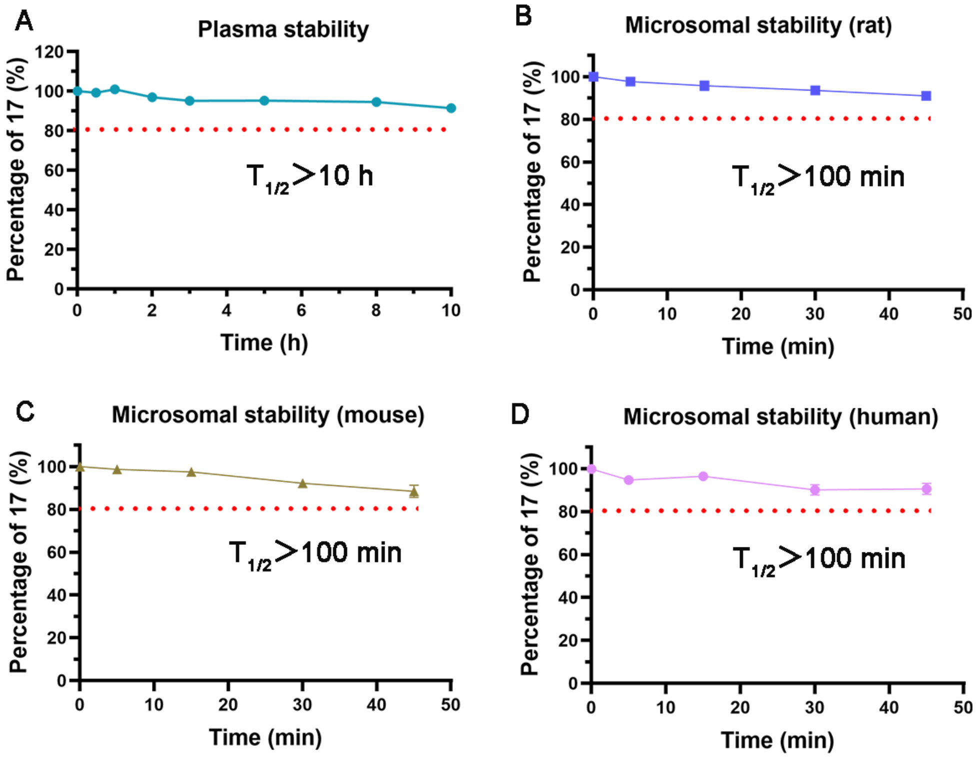

Figure S8. Stability of compound **17**. (A) Plasma stability (rat); (B) Microsomal stability (rat); (C) Microsomal stability (mouse); (D) Microsomal stability (human). Errors: mean ± SD, n = 3.

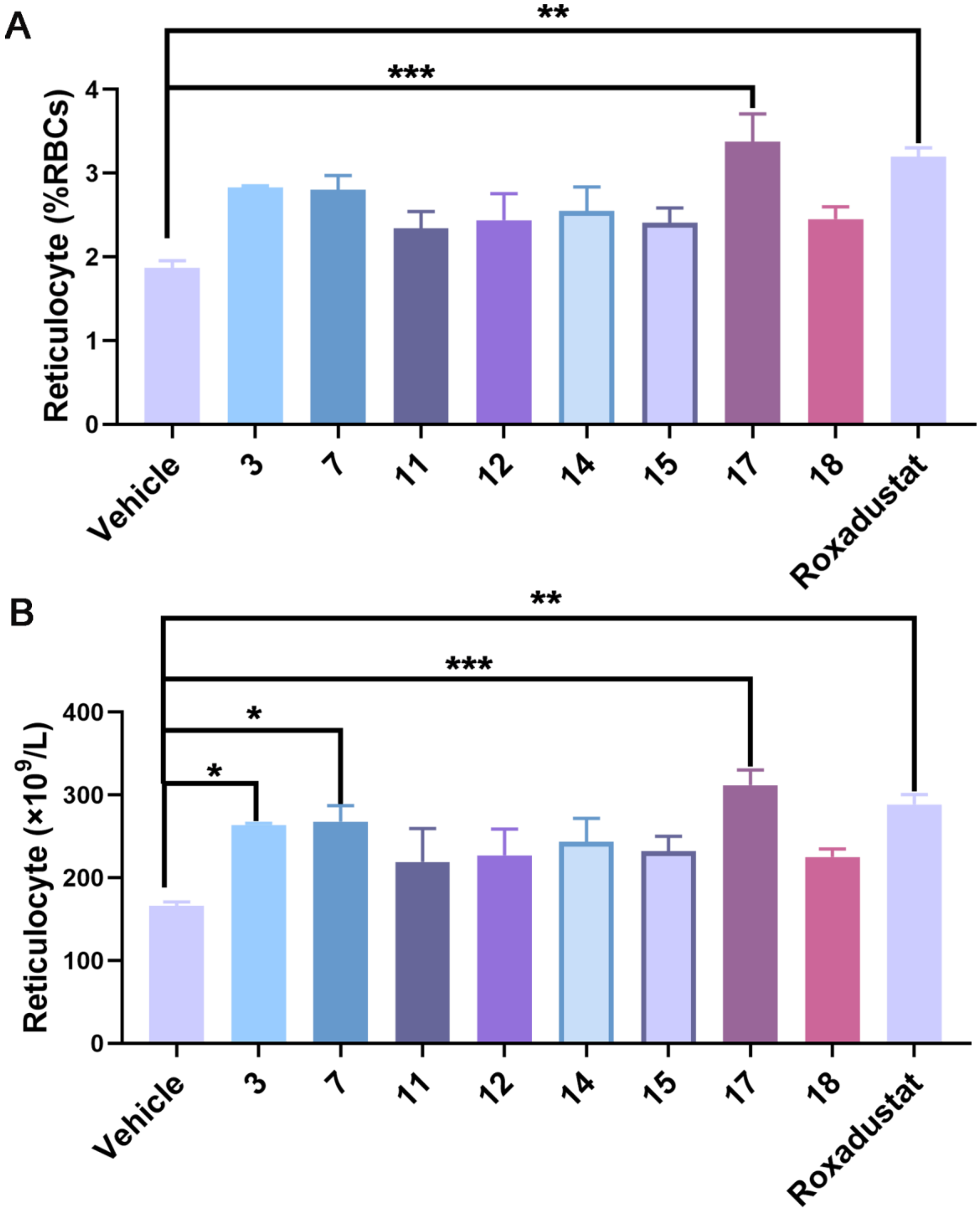

Figure S9. Reticulocyte evaluation of **17** *in vivo*[3]. Reticulocytes and reticulocyte % were measured 72 h (three times *p.o.* administration) after treatment with representative compounds (20 mg·kg^{-1}). Errors: mean ± SEM. The results indicate that compound **17** exhibits a similar reticulocyte effect as does Roxadustat. P values were analyzed by the one-way ANOVA test comparing with the vehicle group (*, $P < 0.05$; **, $P < 0.01$; ***, $P < 0.001$).

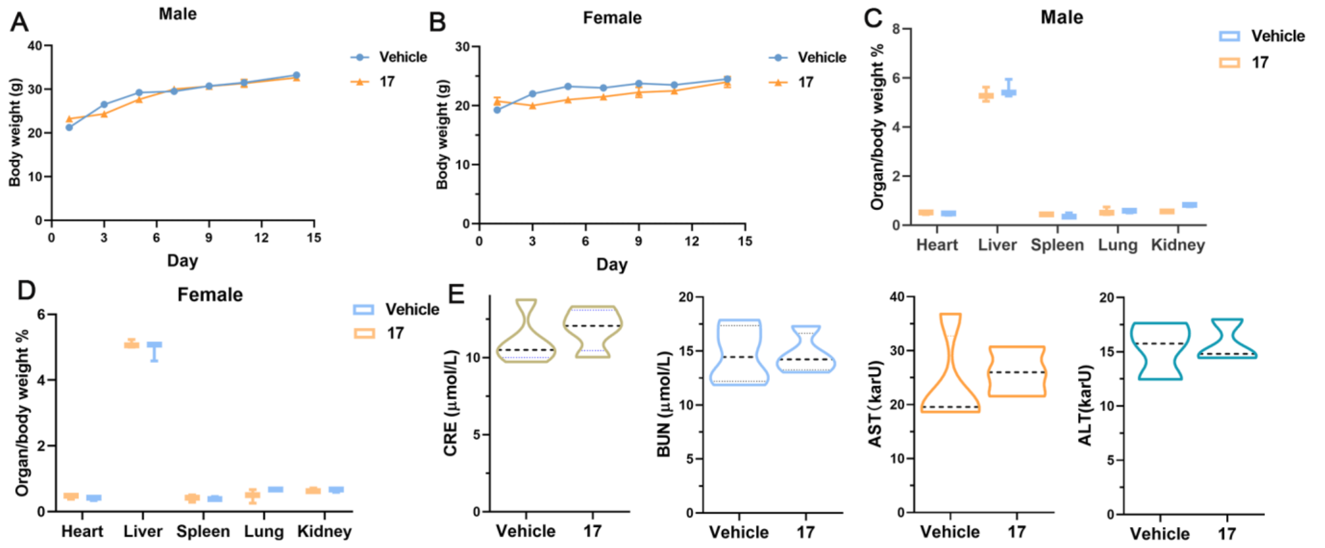

Figure S10. Acute oral toxicity of tests for compound **17**. The effects on body weight (A/B), and organ/body weight ratio (C/D) in mice (400 mg·kg^{-1}). Blood biochemistry analysis of the indicators (E), including alanine aminotransferase (ALT), aspartate aminotransferase (AST), blood urea nitrogen (BUN), and creatinine (CRE) in mice, errors: mean ± SEM. The acute toxicity studies revealed no obvious abnormality after treatment with **17** (LD_{50} >400 mg·kg^{-1}). There were no significant differences in the body weight, organ/body weight ratio, and blood biochemistry indexes (ALT, AST, BUN, and CRE) between the **17**-treated group and the vehicle group.

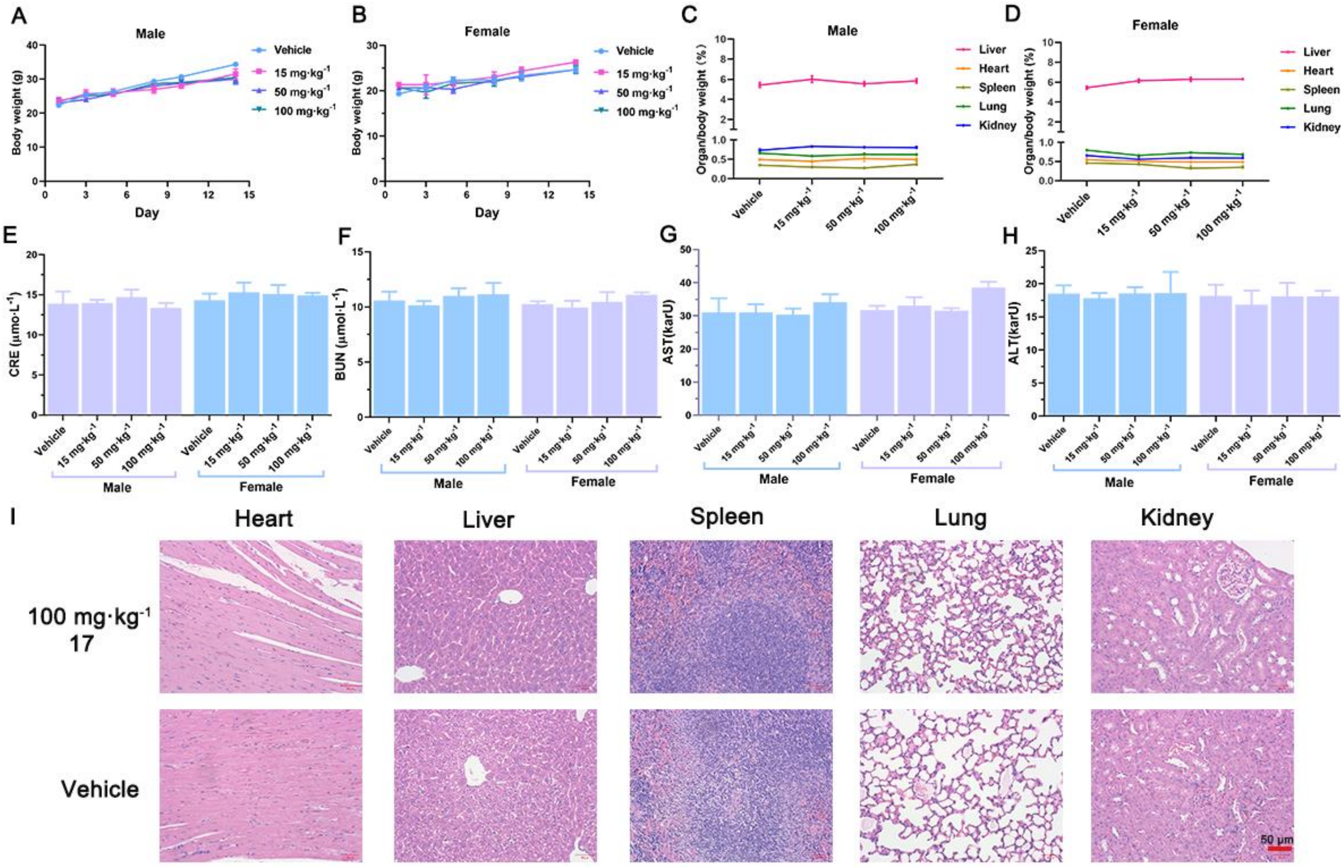

Figure S11. Subacute oral toxicity of compound **17**. The effects of compound **17** on body weight (A/B), and organ/body weight ratio (C/D) in mice (*po*, 15, 50, and 100 mg·kg^{-1}). (E-H) Blood biochemistry analysis of the indexes of BUN ALT, AST, and CRE in ICR mice. (I) H&E staining of major organs from ICR mice after treatment of 0.5% CMCNa and **17** (100 mg·kg^{-1}) on alternate days for two weeks, error: mean ± SEM. The subacute oral toxicity study (14 days) did not exhibit any treatment-associated adverse effects on behavior, body weight, organ/body weight ratio, and blood biochemistry indexes (BUN ALT, AST, and CRE) at doses of 15, 50, and 100 mg·kg^{-1}. The major organs were further investigated using hematoxylin and eosin (H&E) staining. The **17**-treated group (100 mg·kg^{-1}) exhibited normal architecture comparable to the vehicle. Scale bar: 50 μm.

2. *In-vitro* evaluation

2.1 Fluorescence polarization (FP) competition assay of PHD2 [4]

FP assays were performed using a SPARK Multi-Mode Microplate Reader (Tecan) with excitation and emission filters appropriate for the fluorophore used in the binding experiment. Fluorescence polarization experiments were performed in 384-well, flat bottom, black assay plates (#3575, Corning) in a final volume of 60 μL. The final assay buffer contained: 30 nM fluorescence Probe, 20 nM PHD2 (181-426), 10 mM HEPES, pH 7.4, 150 mM NaCl, 0.05% Tween-20, and at the 1% DMSO (except for DMSO tolerance experiments, in which different DMSO concentrations were used as indicated). All plates measured in fluorescence polarization assays were incubated for a minimum of 30 min at RT. Fluorescence polarization was measured from the top of the well with a Tecan SPARK plate reader with polarized filters and optical modules for fluorescein (λ_{ex} = 485 nm ± 25 nm, λ_{em} = 535 nm ± 25 nm). The standard error of the mean (SEM) values, were calculated by dividing the sample standard deviation by the square root of the sample size and are recorded as ± values for IC_{50}. Dose-dependent experiments were performed in the same manner using at least 10 concentrations of the test compounds in 3-fold serial dilutions from 100 μM. For each assay, negative controls (equivalent to 0% displacement) contained the fluorescent ligand, PHD2 (181-426), and 20 μL FP buffer; blank controls contained only the fluorescent ligand and 40 μL buffer. The reaction mixtures were incubated at RT for 60 min, then total fluorescence and fluorescence polarization measurements were taken. The percentage inhibition was calculated using Equation **1**, where mP_{free} is the signal for the free probe (blank control) and mP_{bound} is the signal for the bound probe (negative control).

Equation **1**: $\%\ inhibition = 100*(1 - (mP_{bound} - mP) / (mP_{bound} - mP_{free}))$

IC_{50}s were determined for duplicate measurements by non-linear least-squares analysis using GraphPad Prism 8.0.

The K_i values of the inhibitors were calculated using the Cheng-Prusoff equation.[5]

Cheng-Prusoff equation: $K_i = IC_{50} / (1 + [L]/K_d)$

The K_d value was determined using a constant concentration of probe and titrating with PHD2 protein at increasing concentrations. [L] represents the concentration of the PHD2 protein used in the FP assay.

2.2 In situ inhibitor synthesis and screening (ISISS) assay

Fragments **A** (1 μM), fragments **B** (3 μM), and PHD2 protein (20 nM) were incubated in 384-well plates for 6 h, The fluorescent probe (30 nM) was then added with further incubation for 0.5 h. Fluorescence polarization was measured from the top of the well with a Tecan SPARK plate reader with polarized filters and optical modules for fluorescein (λex = 485 nm ± 25 nm, λem = 535 nm ± 25 nm).

2.3 RT-qPCR assay[6]

The RNA extraction and expression of *EPO* was determined by RT-qPCR. Total RNA of **17** and Roxadustat treated Hep3B cells was isolated using TRIzol (Invitrogen). RT-qPCR analyses of *EPO* (5'-GAGCCCAGAAGGAAGCCATC-3' (forward primer), 5'-CGGAAAGTGTCAGCAGTGATTG-3' (reverse primer)) was performed by using Real-Time qPCR No.1 (QuantStudio 3). *HPRT* 5′-GACCAGTCAACAGGGGACAT-3′ (forward primer) and 5′-AACACTTCGTGGGGTCCTTTTC-3′ (reverse primer) were used for normalization. RT-qPCR analysis of *EPO* was employed the 7500 Fast Real-Time PCR System (ThermoFisher). Each cycle PCR cycle involved: 5 s at 95 °C (denaturation) and 30 s at 60 °C (annealing and extension). A total of 40 cycles was employed.

2.4 Cell viability assay[7]

Hep3B cells in the logarithmic growth phase were seeded into a 96-well plate and maintained under standard conditions (5% CO_2, 37 °C) for 24 h. They were then treated with the compounds (**1-18**, Roxadustat) at 72 h. Each well was subsequently treated with 10 μL CCK-8, then incubated at 37 °C for another 1 h, followed by detection of the absorbance using the multi-mode microplate reader (Tecan SPARK) at 450 nm. Cell viability% = (1-(control group-compound treated group) / (control group-blank group)) *100.

3. Molecular Modeling

A structure of PHD2 in complex with Fe and a 2OG competing inhibitor (PDB ID: 4KBZ) was obtained from PDB website. The structure files of protein and compounds for docking were prepared using the Discovery Studio (DS) 2020 software. A water molecule HOH624, conserved in the active binding site of PHD2, was retained during the docking. Residues of PHD2 around the native ligand (radius = 10 Å) were defined as the binding sites for docking. Subsequently, the ligand was removed; the Fe (II) was retained during the docking. Compounds were docked into the binding sites using GOLD 5.1. Docking procedures were performed using the default setting with 100 genetic algorithm (GA) runs of ligands. For each GA run, a maximum of 125,000 operations was performed. When the top ten solutions possessed RMSD values within 1.5 Å, the docking was terminated. These obtained small molecule-protein complexes were visualized using PyMOL (TM) Molecular Graphic System, Version 2.3.0.

4. Metabolic stability assay [8]

17 was preincubated with microsomes from different species (0.5 mg/mL) at 1 μM for 5 min at 37 °C in 100 mM PBS buffer (pH 7.4). Subsequently, the bioreaction was catalyzed by adding NADPH (1 mM). After co-incubation for different times (0, 15, 30, 45, and 60 min) at 37 °C, the bioreaction was terminated by addition of cold acetonitrile. The clear supernatant samples were investigated by LC-MS/MS. Compound **17** (100 μM) was incubated with rat plasma at different times. The samples were centrifuged at 5000 rpm for 10 min; the supernatants were then analyzed by HPLC.

5. Pharmacodynamic assay [9]

The experimental mice (SPF, C57BL/7J, male) were obtained from SPF (Beijing) Biotechnology Co., Ltd (permit number: SCXK (Jing) 2019-0010). All animal related experiments were conducted under a China Pharmaceutical University (the certification of using the housing facility of laboratory animals: SYXK (Su) 2018-0019) IACUC approved protocol in compliance with the guide for the care and use of laboratory animals (number: 202013705).

5.1 *In vivo* reticulocytes increased assay

Representative compounds and Roxadustat were formulated in normal saline solution. Mice (C57BL/6J, n=5 per dose level) were dosed *p.o.* with a volume of 0.2 mL. After three consecutive days of administration, these test samples were obtained via orbital venous plexus. Subsequently, these samples were analyzed for reticulocytes in the center for new drug safety evaluation and research of China Pharmaceutical University (ADVIA 2120 hematology system).

5.2 *In vivo* EPO increased assay

Plasma EPO was evaluated using the erythropoietin quantikine ELISA kit for mice (MEP00B). **17** and Roxadustat were formulated in normal saline solution. C57BL/6J (n=5) mouse were dosed *p.o*. in 0.2 mL (10, 20, 50 mg·kg^{-1}). After 4 hours, these test samples were obtained via orbital venous plexus. Plasma was collected by centrifugation at 3000 rpm, these plasmas were detected by EPO ELISA.

5.3 Improvement of anemia induced by cisplatin administration

C57BL/6J mice were treated with cisplatin (*po*, 7 mg·kg^{-1}) on days 0, 7, 14, and 28 of the experiment. On the 29th day, the animals were randomized into sham, control, and treated groups (n=5). Then, **17** and Roxadustat (5, 10, 20 mg·kg^{-1}) were administrated for the treatment anemia caused by cisplatin. The mice were treated with the compounds every alternate day for 30 days. the test sample is obtained via orbital venous plexus. The hemoglobin of samples was analyzed in the center for new drug safety evaluation and research at China Pharmaceutical University (ADVIA 2120 hematology system).

6. Subacute and acute oral toxicity assay [10]

The experimental mice (SPF, ICR, male and female) were obtained from Nantong University (permit number: SCXK (Su) 2019-0001). All animal related experiments were conducted under China Pharmaceutical University guidelines (certification of using the housing facility for laboratory animals: SYXK (Su) 2018-0019) IACUC approved protocols in compliance with the guide for the care and use of laboratory animals (number: 202013705).

6.1 Subacute oral toxicity assay

ICR mice were divided into 8 groups (n = 6), including 15 mg·kg^{-1}, 50 mg·kg^{-1}, 100 mg·kg^{-1} and vehicle groups for males and females, respectively. Experimental mice were treated with **17** through oral administration every other day for 14 days. The body weight of the mice was recorded. The mice were then sacrificed and dissected; the heart, kidney, liver, spleen, lung were extracted. Further, the blood was collected. Blood biochemistry assays were carried out as follows: The blood samples were centrifuged at 5000 rpm for 20 min, and the supernatant was subjected to blood biochemistry analysis. Typical functional indicators including urea; CRE, creatinine; AST, aspartate aminotransferase; ALT, blood urea nitrogen; BUN were investigated by the appropriate kit (Nanjing Jiancheng Bioengineering Institute). Additionally, major organs were further investigated using hematoxylin and eosin (H&E) by the pathology and PDX efficacy evaluation center of China Pharmaceutical University.

6.2 Acute oral toxicity assay

ICR mice were divided into 4 groups (n = 6), including **17** 400 mg·kg^{-1}, and vehicle groups for males and females, respectively. Experimental mice were treated with **17** through oral administration once. Body weight and the heart, kidney, liver, spleen, and lung weight were recorded. Blood was collected, and the samples were analyzed by blood biochemistry assays (CRE, AST, ALT, BUN).

7. NMR experiments [11]

A solution of aldehyde **B82** (60 µL, 0.5 M, DMSO-*d$_6$*), 4-methoxyaniline (2 µL, 6.0 M, DMSO-*d$_6$*), mesitylene (100 µL, 0.1 M, DMSO-*d$_6$*), and acylhydrazide **A3** (100 µL, 0.1 M, DMSO-*d$_6$*) was added to a mixture of DMSO-*d$_6$* (188 µL) and FP buffer D_2O (50 µL), in a 5 mm NMR tube; following incubation at room temperature, the sample was evaluated by a Bruker AV-400 instrument.

8. Synthesis

Materials were from commercial sources and were used as received. Organic solvents were concentrated via a rotary evaporator (EYELA OBS-2100) under reduced pressure (IKA VACSTAR digital) at 35 °C - 50 °C. Reactions were monitored using silica gel TLC plates (GF254, 0.25 mm) and visualized under UV (365/254 nm) light. A Mettler MP420 automatic melting point apparatus was used to determine melting points. Proton nuclear magnetic resonance (^{1}H NMR) and carbon nuclear magnetic resonance (^{13}C NMR) spectra were determined using Bruker AV-400/600 instruments. Coupling constants are reported in Hz and multiplicities are quoted as singlet (s), doublet(d), triplet (t), quartet (q), heptet (h), multiplet (m), and broad signal (bs). High-resolution mass spectra (HRMS) were recorded on a Water Q-Tof micro mass spectrometer. The purity (≥95%) of the target compounds for biological testing was evaluated by the HPLC analysis using an Amethyst C18-P (4.6 × 150 mm, 5 µm, Waters) column eluting with methanol/water (90:10 v:v) with a flow rate of 0.5 mL/min; peaks were detected at 254 nm under UV.

Synthetic Procedures

Synthesis of A1

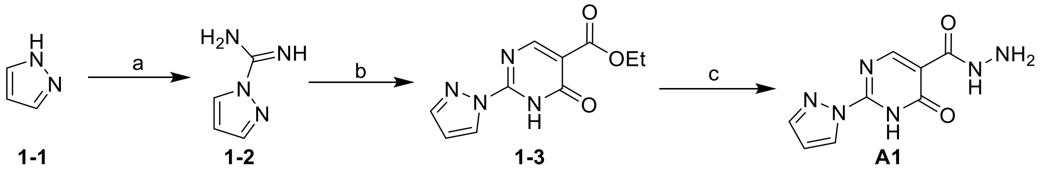

1-1 **1-2** **1-3** **A1**

Scheme 1. *Reagents and conditions*: (a) cyanamide, HCl (1,4-dioxane), 70 °C, 6 h, 87%; (b) NaOEt, EtOH, diethyl 2-(ethoxymethylene)malonate, 80 °C, 4 h, 85%;

(c) $N_2H_4 \cdot H_2O$, MeOH, 80 °C, 6.0 h, 41 %.

1-3 were synthesized according to procedures described elsewhere[4]. The ^1H spectra of **1-3** were identical to those reported[4]. Ethyl 6-oxo-2-(1*H*-pyrazol-1-yl)-1,6-dihydropyrimidine-5-carboxylate (**1-3**) was a white solid. ^1H NMR (400 MHz, DMSO-d_6) *δ* 13.39 (s, 1H), 8.63 (s, 1H), 8.62 (s, 1H), 7.98 (s, 1H), 6.68 (m, 1H), 4.26 (q, *J* = 7.1 Hz, 2H), 1.29 (t, *J* = 7.1 Hz, 3H).

Synthesis of 6-oxo-2-(1*H*-pyrazol-1-yl)-1,6-dihydropyrimidine-5-carbohydrazide (**A1**)

To **1-3** (0.263 g, 1.1 mmol) in MeOH (8 mL) was added $N_2H_4 \cdot H_2O$ (0.101 g, 2.0 mmol). The reaction was heated at 80 °C for 6.0 h, then cooled to room temperature. Subsequently, the precipitate was filtered and washed with ice-cold MeOH to afford the white solid **A1** (0.102 g, 41%). mp 266.8−268.3 °C. ^1H NMR (400 MHz, DMSO-d_6) *δ* 11.12 (s, 1H), 8.54 (s, 2H), 7.70 (s, 1H), 6.48 (s, 1H), 4.44 (s, 2H). HRMS (ESI): calcd. for $C_8H_8N_6O_2$ $[M + H]^+$ 221.0781, found 221.0779. HPLC: t_R = 1.52 min, 100.0%.

Synthesis of A2

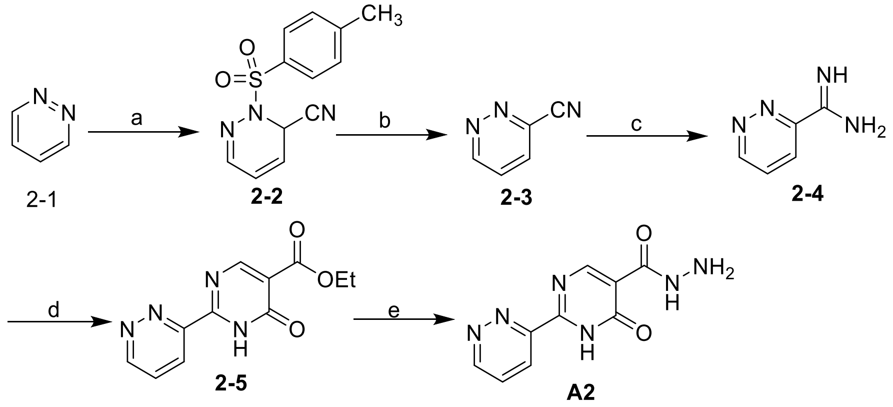

Scheme 2. *Reagents and conditions*: (a) TMSCN, $AlCl_3$, *p*-TosCl, DCM, rt, 5 h, 75%; (b) DBU, THF, rt, 6 h; DBU, THF, rt, 90%; (c) NaOMe, NH_4Cl, MeOH, rt, 82%; (d) NaOEt, EtOH, diethyl 2-(ethoxymethylene)malonate, 80 °C, 4 h, 53%; (e) $N_2H_4 \cdot H_2O$, MeOH, 80 °C, 6.0 h, 51%.

The intermediate **2-4** was synthesized as reported[4]. The ^1H NMR spectrum of **2-4** was identical to that reported[4]. Ethyl 6-oxo-2-(pyridazin-3-yl)-1,6-dihydropyrimidine-5-carboxylate (**2-4**) was a white solid. ^1H NMR (400 MHz, DMSO-d_6) *δ* 9.51 (s, 1H), 9.38 (d, *J* = 4.8 Hz, 1H), 8.04 (d, *J* = 8.4 Hz, 1H), 7.88 (dd, *J* = 8.5, 5.0 Hz, 1H), 4.38 (q, *J* = 7.0 Hz, 2H), 1.37 (t, *J* = 7.1 Hz, 3H).

Synthesis of 6-oxo-2-(pyridazin-3-yl)-1,6-dihydropyrimidine-5-carbohydrazide (**A2**).

To intermediate **2-5** (0.372 g, 1.6 mmol) in MeOH (10 mL) was added $N_2H_4 \cdot H_2O$ (0.151 g, 3.0 mmol). The reaction was heated at 80 °C for 6.0 h, then cooled to room temperature. Subsequently, the precipitate was filtered and rinsed with ice-cold MeOH to afford the white solid **A2** (0.181 g, 51%). mp 241.5−242.3 °C. ^1H NMR (400 MHz, DMSO-d_6) *δ* 11.31 (s, 1H), 9.30 (d, *J* = 4.8 Hz, 1H), 8.70 (s, 1H), 8.45 (d, *J* = 8.6 Hz, 1H), 7.82 (dd, *J* = 8.6, 5.0 Hz, 1H), 7.12 (s, 2H). HRMS (ESI): calcd. for $C_9H_8N_6O_2$ $[M + H]^+$ 233.0781, found 233.0777. HPLC: t_R = 1.47 min, 100.0%.

Synthesis of A3

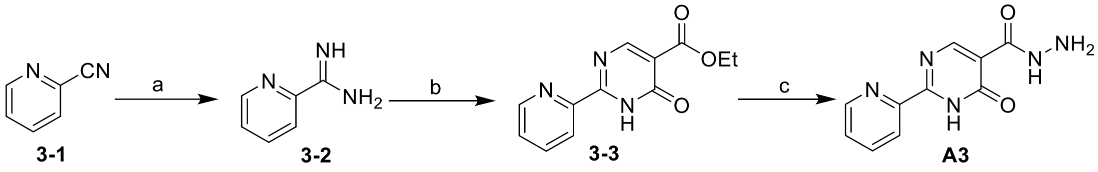

Scheme 3. *Reagents and conditions*: (a) NaOMe, NH_4Cl, MeOH, rt, 89% ;(b) NaOEt, EtOH, diethyl 2-(ethoxymethylene) malonate, 80 °C, 4 h, 71%; (c) $N_2H_4 \cdot H_2O$, MeOH, 80 °C, 6.0 h, 52%.

The intermediate **3-3** was synthesized as reported[4]. The ^1H spectrum of **3-3** was identical to that reported[4]. Ethyl 6-oxo-2-(pyridin-2-

yl)-1,6-dihydropyrimidine-5-carboxylate (**3-3**) was a white solid. ^{1}H NMR (400 MHz, DMSO-*d*$_6$) *δ* 8.68 (d, *J* = 4.1 Hz, 1H), 8.63 (s, 1H), 8.38 (d, *J* = 7.9 Hz, 1H), 7.94 (td, *J* = 7.8, 1.7 Hz, 1H), 7.54 – 7.48 (m, 1H), 4.20 (q, *J* = 7.1 Hz, 2H), 1.27 (t, *J* = 7.1 Hz, 3H).

Synthesis of 6-oxo-2-(pyridin-2-yl)-1,6-dihydropyrimidine-5-carbohydrazide (**A3**).
To intermediate **3-3** (0.420 g, 1.7 mmol) in MeOH (10 mL) was added $N_2H_4{\cdot}H_2O$ (0.173 g, 3.4 mmol). The reaction was heated at 80 °C for 6.0 h, then cooled to room temperature. Subsequently, the precipitate was filtered and rinsed with ice-cold MeOH to afford the white solid **A3** (0.206 g, 52%). mp > 300 °C. ^{1}H NMR (400 MHz, DMSO-*d*$_6$) *δ* 11.25 (s, 1H), 8.68 (s, 2H), 8.39 (d, *J* = 7.8 Hz, 1H), 7.94 (t, *J* = 7.3 Hz, 1H), 7.56 – 7.44 (m, 1H), 5.03 (s, 2H). HRMS (ESI): calcd. for $C_8H_8N_6O_2$ [M + H]$^+$ 232.0829, found 232.0832. HPLC: t_R = 1.61 min, 100.0%.

Synthesis of A4

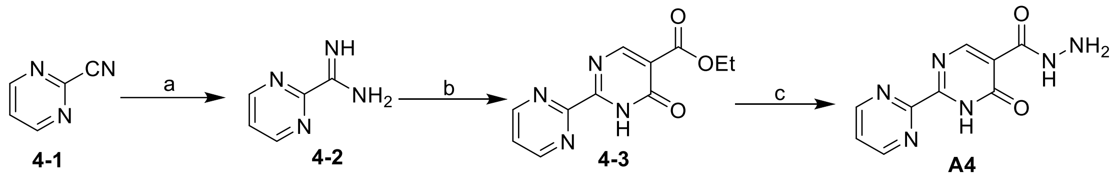

Scheme 4. *Reagents and conditions*: (a) NaOMe, NH_4Cl, MeOH, rt, 89% ;(b) NaOEt, EtOH, diethyl 2-(ethoxymethylene) malonate, 80 °C, 4 h, 71%; (c) $N_2H_4{\cdot}H_2O$, MeOH, 80 °C, 6.0 h, 55%.

The intermediate **4-3** was synthesized as reported[4]. The ^{1}H spectrum of **4-3** was identical to that reported[4]. Ethyl 6-oxo-1,6-dihydro-[2,2'-bipyrimidine]-5-carboxylate (**4-3**) was a white solid. ^{1}H NMR (400 MHz, DMSO-*d*$_6$) *δ* 8.96 (d, *J* = 4.9 Hz, 2H), 8.60 (s, 1H), 7.63 (t, *J* = 4.9 Hz, 1H), 4.22 (q, *J* = 7.1 Hz, 2H), 1.29 (t, *J* = 7.1 Hz, 3H).

Synthesis of 6-oxo-1,6-dihydro-[2,2'-bipyrimidine]-5-carbohydrazide (**A4**).
To intermediate **4-3** (0.370 g, 1.5 mmol) in MeOH (8 mL) was added $N_2H_4{\cdot}H_2O$ (0.151 g, 3.0 mmol). The reaction was heated at 80 °C for 6.0 h, then cooled to room temperature. Subsequently, the precipitate was filtered and rinsed with ice-cold MeOH to afford the white solid **A4** (0.192 g, 55%). mp ＞300 °C. ^{1}H NMR (400 MHz, DMSO-*d*$_6$) *δ* 11.36 (s, 1H), 8.93 (s, 2H), 8.68 (s, 1H), 7.59 (s, 1H), 4.56 (s, 2H). HRMS (ESI): calcd. for $C_9H_8N_6O_2$ [M + H]$^+$ 233.0787, found 233.0785. HPLC: t_R = 1.47 min, 98.39%.

Synthesis of A5

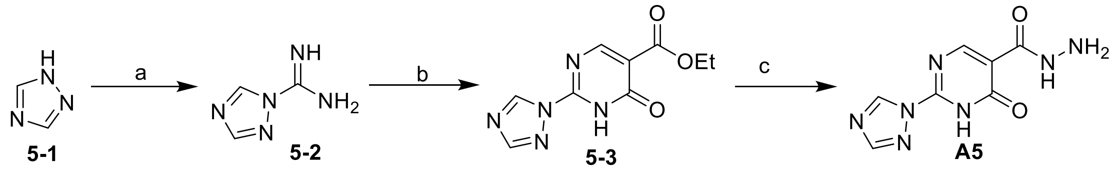

Scheme 5. *Reagents and conditions*: (a) cyanamide, HCl (1,4-dioxane), 70 °C, 6 h, 81%;(b) NaOEt, EtOH, diethyl 2-(ethoxymethylene) malonate, 80 °C, 4 h, 63%; (c) $N_2H_4{\cdot}H_2O$, MeOH, rt 8.0 h, 60%.

The intermediate **5-3** was synthesized as reported[4]. The ^{1}H spectrum of **5-3** was identical to that reported[4]. Ethyl 6-oxo-2-(1*H*-1,2,4-triazol-1-yl)-1,6-dihydropyrimidine-5-carboxylate (**5-3**) was a white solid. ^{1}H NMR (400 MHz, DMSO-*d*$_6$) *δ* 9.23 (s, 1H), 8.50 (s, 1H), 8.16 (s, 1H), 4.19 (q, *J* = 7.1 Hz, 2H), 1.27 (t, *J* = 7.1 Hz, 3H).

Synthesis of 6-oxo-2-(1*H*-1,2,4-triazol-1-yl)-1,6-dihydropyrimidine-5-carbohydrazide (**A5**)
To intermediate **5-3** (0.425 g, 1.8 mmol) in MeOH (12 mL) was added $N_2H_4{\cdot}H_2O$ (0.184 g, 3.6 mmol). The reaction was heated at 80 °C for 8.0 h, then cooled to room temperature. Subsequently, the precipitate was filtered and rinsed with ice-cold MeOH to afford the white solid **A4** (0.125 g, 31%). mp ＞300 °C. ^{1}H NMR (400 MHz, DMSO-*d*$_6$) *δ* 11.12 (s, 1H), 9.21 (s, 1H), 8.54 (s, 1H), 8.15 (s, 1H), 4.41 (s, 2H). HRMS (ESI): calcd. for $C_7H_7N_7O_2$ [M + H]$^+$ 222.0734, found 222.0727. HPLC: t_R = 1.47 min, 100.0%.

Synthesis of 1-18

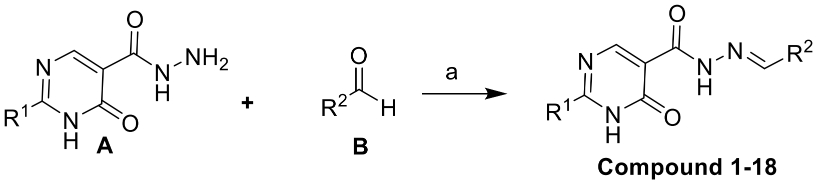

Compound 1-18

Scheme 6. *Reagents and conditions*: (a) DMSO, H_2O, 4-methoxyaniline, rt, 24 h, yield% ≥84%.

N'-(2,3-dihydroxybenzylidene)-6-oxo-2-(1*H*-pyrazol-1-yl)-1,6-dihydropyrimidine-5-carbohydrazide (**1**, **A1B74**). A solution of **A1** (0.132 g, 0.6 mmol), 2,3-dihydroxybenzaldehyde (**B74**, 0.138 g, 1.0 mmol), and 4-methoxyaniline (0.025 g, 0.20 mmol) in DMSO (1 mL) and H_2O (3 mL) was stirred at room temperature for 24 h. The mixture was poured into 5 mL H_2O and the precipitate was collected by filtration, then washed with cooled ethyl acetate to give the target compound **1** (**A1B74**, 0.186 g, 91%) as a slate gray solid. mp > 300 °C. ^{1}H NMR (400 MHz, DMSO-*d*$_6$) δ 14.22 (bs, 1H), 11.50 (s, 1H), 9.08 (s, 1H), 8.66 (s, 1H), 8.54 (s, 2H), 7.73 (s, 1H), 6.95 (d, *J* = 7.4 Hz, 1H), 6.83 (d, *J* = 8.0 Hz, 1H), 6.77 – 6.69 (m, 1H), 6.48 (s, 1H). HRMS (ESI): calcd. for $C_{17}H_{13}N_5O_3$ [M + H]$^+$ 341.0993, found 341.0997. HPLC: t_R = 1.60 min, 95.12%.

N'-(2,3-dihydroxybenzylidene)-6-oxo-2-(pyridazin-3-yl)-1,6-dihydropyrimidine-5-carbohydrazide (**2**, **A2B74**). A solution of **A2** (0.139 g, 0.6 mmol), 2,3-dihydroxybenzaldehyde (**B74**, 0.138 g, 1.0 mmol), and 4-methoxyaniline (0.025 g, 0.20 mmol) in DMSO (1 mL) and H_2O (3 mL) was stirred at room temperature for 24 h. The mixture was poured into 5 mL H_2O and the precipitate was collected by filtration, then washed with cooled ethyl acetate to give the target compound **2** (**A2B74**, 0.193 g, 92%) as a state gray solid. mp > 300 °C$^{.1}$H NMR (400 MHz, DMSO-*d*$_6$) *δ* 14.12 (bs, 1H), 12.82 (s, 1H), 11.08 (s, 1H), 9.50 (d, *J* = 4.8 Hz, 1H), 9.22 (s, 1H), 8.72 (s, 1H), 8.67 (s, 1H), 8.55 (d, *J* = 8.5 Hz, 1H), 8.01 (dd, *J* = 8.4, 5.1 Hz, 1H), 6.99 (d, *J* = 7.8 Hz, 1H), 6.87 (d, *J* = 7.7 Hz, 1H), 6.76 (t, *J* = 7.8 Hz, 1H). HRMS (ESI): calcd. for $C_{16}H_{12}N_6O_4$ [M + H]$^+$ 353.0993, found 353.0995. HPLC: t_R = 1.47 min, 95.11%.

N'-(2,3-dihydroxybenzylidene)-6-oxo-2-(pyridin-2-yl)-1,6-dihydropyrimidine-5-carbohydrazide (**3**, **A3B74**). A solution of **A3** (0.137 g, 0.6 mmol), 2,3-dihydroxybenzaldehyde (**B74**, 0.138 g, 1.0 mmol), and 4-methoxyaniline (0.025 g, 0.20 mmol) in DMSO (1 mL) and H_2O (3 mL) was stirred at room temperature for 24 h. The mixture was poured into 5 mL H_2O and the precipitate was collected by filtration, then washed with cooled ethyl acetate to give the target compound **3** (**A2B74**, 0.201 g, 97%) as a brown solid. mp > 300 °C. ^{1}H NMR (400 MHz, DMSO-*d*$_6$) *δ* 13.41 (bs, 1H), 12.73 (s, 1H), 11.07 (s, 1H), 9.24 (s, 1H), 8.84 (d, *J* = 4.3 Hz, 1H), 8.69 (s, 1H), 8.67 (s, 1H), 8.43 (d, *J* = 7.9 Hz, 1H), 8.13(td, *J* = 7.9, 1.4 Hz 1H), 7.75 (dd, *J* = 6.9, 4.8 Hz, 1H), 6.99 (dd, *J* = 8.9, 1.2 Hz, 1H), 6.87 (dd, *J* = 7.8, 1.3 Hz, 1H), 6.76 (t, *J* = 7.8 Hz, 1H). ^{13}C NMR (125 MHz, DMSO-*d*$_6$) δ 149.93, 146.65, 146.07, 138.78, 127.94, 123.81, 123.03, 120.88, 119.69, 119.13, 118.12, 115.16. HRMS (ESI): calcd. for $C_{17}H_{13}N_5O_4$ [M + H]$^+$ 352.1040, found 352.1032. HPLC: t_R = 1.67 min, 97.39%.

N'-(2,3-dihydroxybenzylidene)-6-oxo-1,6-dihydro-[2,2'-bipyrimidine]-5-carbohydrazide (**4**, **A4B74**). A solution of **A4** (0.139 g, 0.6 mmol), 2,3-dihydroxybenzaldehyde (**B74**, 0.138 g, 1.0 mmol), and 4-methoxyaniline (0.025 g, 0.20 mmol) in DMSO (1 mL) and H_2O (3 mL) was stirred at room temperature for 24 h. The mixture was poured into 5 mL H_2O and the precipitate was collected by filtration, then washed with cooled ethyl acetate to give the target compound **4** (**A4B74**, 0.190 g, 90%) as a light yellow solid. mp > 300 °C. ^{1}H NMR (400 MHz, DMSO-*d*$_6$) *δ* 13.67 (bs, 1H), 11.30 (bs, 1H), 9.15 (s, 1H), 9.00 (d, *J* = 4.0 Hz, 2H), 8.73 (s, 1H), 8.60 (s, 1H), 7.67 (d, *J* = 5.0 Hz, 1H), 6.96 (d, *J* = 7.7 Hz, 1H), 6.85 (d, *J* = 7.6 Hz, 1H), 6.78 – 6.69 (m, 1H). HRMS (ESI): calcd. for $C_{14}H_{11}N_7O_4$ [M + H]$^+$ 353.0993, found 353.0986. HPLC: t_R = 1.47 min, 96.77%

N'-(2,3-dihydroxybenzylidene)-6-oxo-2-(1*H*-1,2,4-triazol-1-yl)-1,6-dihydropyrimidine-5-carbohydrazide (**5**, **A5B74**). A solution of **A5** (0.132 g, 0.6 mmol), 2,3-dihydroxybenzaldehyde (**B74**, 0.138 g, 1.0 mmol), and 4-methoxyaniline (0.025 g, 0.20 mmol) in DMSO (1 mL) and H_2O (3 mL) was stirred at room temperature for 24 h. The mixture was poured into 5 mL H_2O and the precipitate was collected by filtration, then washed with cooled ethyl acetate to give the target compound **5** (**A5B74**, 0.185 g, 91%) as an olive solid. mp > 300 °C. ^{1}H NMR (400 MHz, DMSO-*d*$_6$) *δ* 14.09 (s, 1H), 11.45 (s, 1H), 9.24 (s, 1H), 9.10 (s, 1H), 8.67 (s, 1H), 8.54 (s, 1H), 8.17 (s, 1H), 6.95 (d, *J* = 7.2 Hz, 1H), 6.83 (d, *J* = 7.0 Hz, 1H), 6.74 (t, *J* = 7.7 Hz, 1H). HRMS (ESI): calcd. for $C_{14}H_{11}N_7O_4$ [M + H]$^+$ 342.0945, found 342.0947. HPLC: t_R = 1.47 min, 96.21%

N'-(3,4-dihydroxybenzylidene)-6-oxo-2-(pyridin-2-yl)-1,6-dihydropyrimidine-5-carbohydrazide (**6, A3B59**). The preparation method of compound **6** was analogous to that for **3**. Yield 93%. White solid. mp 294.7−295.2 °C. ^{1}H NMR (400 MHz, DMSO-d_6) *δ* 13.22(bs, 1H), 12.51 (s, 1H), 9.43 (s, 1H), 9.29 (s, 1H), 8.84 (d, *J* = 4.3 Hz, 1H), 8.67 (s, 1H), 8.42 (d, *J* = 7.8 Hz, 1H), 8.24 (s, 1H), 8.13 (td, *J* = 8.0 Hz, 1.1 Hz, 1H), 7.74 (dd, *J* = 6.9, 4.8 Hz, 1H), 7.27 (d, *J* = 3.0 Hz, 1H), 7.02 (m, 2H), 6.79 (d, *J* = 8.1 Hz, 1H). ^{13}C NMR (125 MHz, DMSO-d_6) δ 159.36, 149.90, 149.83, 148.71, 148.29, 146.14, 138.84, 128.06, 125.99, 123.73, 121.44, 116.02, 113.45. HRMS (ESI): calcd. for $C_{17}H_{13}N_5O_4$ $[M + H]^+$ 352.1040, found 352.1038. HPLC: t_R = 1.63 min, 98.03%.

6-oxo-2-(pyridin-2-yl)-*N*'-(3,4,5-trihydroxybenzylidene)-1,6-dihydropyrimidine-5-carbohydrazide (**7, A3B82**). The preparation method of compound **7** was analogous to that for **3**. Yield 87%. White solid. mp > 300 °C. ^{1}H NMR (400 MHz, DMSO-d_6) *δ* 13.18 (bs, 1H), 12.46 (bs, 1H), 9.15 (s, 2H), 8.84 (d, *J* = 3.7 Hz, 1H), 8.67 (s, 2H), 8.42 (d, *J* = 7.9 Hz, 1H), 8.11-8.15 (m, 2H), 7.76 – 7.73 (m, 1H), 6.75 (s, 2H). ^{13}C NMR (125 MHz, DMSO-d_6) *δ* 159.87, 150.77, 149.91, 146.63, 146.06, 138.77, 127.92, 123.80, 123.02, 121.69, 120.86, 119.67, 119.12, 118.10, 115.15. HRMS (ESI): calcd. for $C_{17}H_{13}N_5O_5$ [M + H]+ 368.0989, found 368.0963. HPLC: t_R = 1.40 min, 96.65%.

N'-(2-hydroxybenzylidene)-6-oxo-2-(pyridin-2-yl)-1,6-dihydropyrimidine-5-carbohydrazide (**8, A3B83**). The preparation method of compound **8** was analogous to that for **3**. Yield 94%. Yellow solid. mp > 300 °C. ^{1}H NMR (400 MHz, DMSO-d_6) *δ* 13.47 (bs, 1H), 12.74 (bs, 1H), 11.19 (s, 1H), 8.84 (d, *J* = 4.2 Hz, 1H), 8.70 (s, 2H), 8.43 (d, *J* = 7.8 Hz, 1H), 8.13 (t, *J* = 7.7 Hz, 1H), 7.75 (dd, *J* = 7.0, 5.2 Hz, 1H), 7.56 (d, *J* = 7.6 Hz, 1H), 7.36 – 7.28 (m, 1H), 6.95 (d, *J* = 7.7 Hz, 2H). HRMS (ESI): calcd. for $C_{17}H_{13}N_5O_3$ $[M + H]^+$ 336.1091, found 336.1085.HPLC: t_R = 1.84 min, 99.63%.

N'-(2,5-dihydroxybenzylidene)-6-oxo-2-(pyridin-2-yl)-1,6-dihydropyrimidine-5-carbohydrazide (**9, A3B85**). The preparation method of compound **9** was analogous to that for **3**. Yield 93%. Brown solid. mp > 300 °C. ^{1}H NMR (400 MHz, DMSO-d_6) *δ* 13.37 (bs, 1H), 12.61 (s, 1H), 11.33 (s, 1H), 10.02 (s, 1H), 8.84 (d, *J* = 4.3 Hz, 1H), 8.67 (s, 1H), 8.56 (s, 1H), 8.42 (d, *J* = 7.8 Hz, 1H), 8.15 – 8.08 (m, 1H), 7.74 (dd, *J* = 7.0, 4.9 Hz, 1H), 7.33 (d, *J* = 8.4 Hz, 1H), 6.38 (dd, *J* = 8.4, 2.0 Hz, 1H), 6.32 (d, *J* = 1.9 Hz, 1H). HRMS (ESI): calcd. for $C_{17}H_{13}N_5O_4$ $[M + H]^+$ 352.1040, found 352.1034. HPLC: t_R = 1.67 min, 97.18%.

6-oxo-2-(pyridin-2-yl)-*N*'-(2,3,4-trihydroxybenzylidene)-1,6-dihydropyrimidine-5-carbohydrazide (**10, A3B87**). The preparation method of compound **10** was analogous to that for **3**. Yield 94%. Yellow solid. mp > 300 °C. ^{1}H NMR (400 MHz, DMSO-d_6) *δ* 12.67 (bs, 1H), 11.37 (s, 1H), 9.51 (s, 1H), 8.84 (d, *J* = 4.1 Hz, 1H), 8.68 (s, 1H), 8.54 (s, 1H), 8.50 (s, 1H), 8.42 (d, *J* = 7.7 Hz, 1H), 8.15 – 8.08 (m, 1H), 7.74 (dd, *J* = 7.6, 4.3 Hz, 1H), 6.82 (d, *J* = 8.5 Hz, 1H), 6.41 (d, *J* = 8.4 Hz, 1H). HRMS (ESI): calcd. for $C_{17}H_{13}N_5O_5$ $[M + H]^+$ 368.0989, found 368.0978.HPLC: t_R = 1.67 min, 97.21%.

2-((2-(6-oxo-2-(pyridazin-3-yl)-1,6-dihydropyrimidine-5-carbonyl)hydrazineylidene)methyl)benzoic acid (**11, A2B91**). The preparation method of compound **11** was analogous to that for **2**. Yield 91%. Pale yellow solid. mp > 300 °C.^{1}H NMR (400 MHz, DMSO-d_6) *δ* 13.20 (s, 1H), 9.45 (d, *J* = 4.0 Hz, 1H), 9.07 (s, 1H), 8.74 (s, 1H), 8.49 (d, *J* = 8.2 Hz, 1H), 8.04 (d, *J* = 7.7 Hz, 1H), 7.95 (m, 2H), 7.64 (t, *J* = 7.6 Hz, 1H), 7.54 (t, *J* = 7.4 Hz, 1H). HRMS (ESI): calcd. for $C_{14}H_{17}N_2O_4$ $[M + H]^+$ 365.0993, found 365.1013. HPLC: t_R = 1.47 min, 97.09%

4-((2-(6-oxo-2-(pyridin-2-yl)-1,6-dihydropyrimidine-5-carbonyl)hydrazineylidene)methyl)benzoic acid (**12, A3B93**). The preparation method of compound **12** was analogous to that for **3**. Yield 96%. White solid. mp > 300 °C. ^{1}H NMR (300 MHz, DMSO-d_6) *δ* 12.94 (bs, 1H), 8.83 (d, *J* = 3.9 Hz, 1H), 8.70 (s, 1H), 8.55 (s, 1H), 8.42 (d, *J* = 7.6 Hz, 1H), 8.15 – 8.08 (m, 1H), 8.02 (d, *J* = 8.2 Hz, 2H), 7.88 (d, *J* = 8.3 Hz, 2H), 7.73 (dd, *J* = 6.8, 4.3 Hz, 1H). HRMS (ESI): calcd. for $C_{18}H_{13}N_5O_4$ $[M + H]^+$ 364.1040, found 380.1032. HPLC: t_R = 1.76 min, 97.37%.

4-((2-(6-oxo-1,6-dihydro-[2,2'-bipyrimidine]-5-carbonyl)hydrazineylidene)methyl)benzoic acid (**13, A4B93**). The preparation method of compound **13** was analogous to that for **4**. Yield 93%. White solid. mp > 300 °C. ^{1}H NMR (400 MHz, DMSO-d_6) *δ* 13.70 (bs, 1H), 13.15 (bs, 1H), 12.75 (s, 1H), 9.13 (d, *J* = 4.8 Hz, 2H), 8.74 (s, 1H), 8.56 (s, 1H), 8.03 (d, *J* = 8.1 Hz, 2H), 7.88 (d, *J* = 8.1 Hz, 2H), 7.82 (t, *J* = 4.8 Hz, 1H). HRMS (ESI): calcd. for $C_{14}H_{17}N_2O_4$ $[M + H]^+$ 365.0993, found 365.0992. HPLC: t_R = 1.40 min, 97.11%.

2-hydroxy-5-((2-(6-oxo-2-(pyridazin-3-yl)-1,6-dihydropyrimidine-5-carbonyl)hydrazineylidene)methyl)benzoic acid (**14, A2B96**). The preparation method of compound **14** was analogous to that for **2**. Yield 93%. White solid. mp > 300 °C. ^{1}H NMR (400 MHz, DMSO-d_6)

δ 13.90 (bs, 1H), 12.53 (bs, 1H), 9.52 (d, *J* = 3.8 Hz, 1H), 8.68 (bs, 1H), 8.56 (d, *J* = 8.4 Hz, 1H), 8.39 (s, 1H), 8.18 (s, 1H), 8.03 (dd, *J* = 8.5, 5.1 Hz, 1H), 7.81 (d, *J* = 7.6 Hz, 1H), 6.94 (d, *J* = 8.4 Hz, 1H) HRMS (ESI): calcd. for $C_{14}H_{17}N_2O_4$ $[M + H]^+$ 381.0942, found 381.0944. HPLC: t_R = 1.38 min, 100.0%.

2-hydroxy-5-((2-(6-oxo-2-(pyridin-2-yl)-1,6-dihydropyrimidine-5-carbonyl)hydrazineylidene)methyl)benzoic acid (**15, A3B96**). The preparation method of compound **15** was analogous to that for **3**. Yield 90%. Yellow solid. mp > 300 °C. ^{1}H NMR (400 MHz, DMSO-d_6) δ 13.49 (bs, 1H), 12.82 (bs, 1H), 8.84 (d, *J* = 4.3 Hz, 1H), 8.42 (d, *J* = 7.0 Hz, 2H), 8.21 (d, *J* = 1.8 Hz, 1H), 8.16 – 8.09 (m, 1H), 7.91 (dd, *J* = 8.7, 1.9 Hz, 1H), 7.75 (dd, *J* = 7.0, 5.1 Hz, 1H), 7.06 (d, *J* = 8.6 Hz, 1H). ^{13}C NMR (125 MHz, DMSO-d_6) δ 171.93, 163.18, 149.90, 148.62, 148.12, 138.86, 134.42, 130.15, 128.13, 128.12, 125.86, 123.79, 118.32, 114.10. HRMS (ESI): calcd. for $C_{18}H_{13}N_5O_5$ $[M + H]^+$ 380.0989, found 380.0989. HPLC: t_R = 1.37 min, 98.06%

N'-(3-chloro-2-hydroxybenzylidene)-6-oxo-2-(pyridazin-3-yl)-1,6-dihydropyrimidine-5-carbohydrazide (**16, A2B100**). The preparation method of compound **16** was analogous to that for **2**. Yield 93%. Pale yellow solid. mp > 300 °C. ^{1}H NMR (400 MHz, DMSO-d_6) δ 12.93 (bs, 1H), 12.25 (s, 1H), 9.51 (d, *J* = 3.9 Hz, 1H), 8.80 (s, 1H), 8.73 (s, 1H), 8.55 (d, *J* = 7.9 Hz, 1H), 8.02 (dd, *J* = 8.6, 5.0 Hz, 1H), 7.53 – 7.50 (m, 1H), 7.50 – 7.47 (m, 1H), 6.99 (t, *J* = 7.9 Hz, 1H). HRMS (ESI): calcd. for $C_{16}H_{11}ClN_6O_3$ $[M + H]^+$ 371.0654, found 371.0634. HPLC: t_R = 1.70 min, 98.94%.

N'-(2-chloro-3-hydroxybenzylidene)-6-oxo-2-(pyridazin-3-yl)-1,6-dihydropyrimidine-5-carbohydrazide (**17, A2B101**). The preparation method of compound **17** was analogous to that for **2**. Yield 85%. Gold solid. mp > 300 °C. ^{1}H NMR (400 MHz, DMSO-d_6) δ 14.26 (s, 1H, -CON*H*C-), 10.43 (s, 1H, -Ph-O*H*), 9.31 (dd, *J* = 4.9, 1.5 Hz, 1H, pyridazine-*H*), 8.80 (s, 1H, pyrimidine-*H*), 8.62 (s, 1H, -N=C*H*-), 8.41 (d, *J* = 7.4 Hz, 1H, pyridazine-*H*), 7.82 (dd, *J* = 8.5, 5.0 Hz, 1H, pyridazine-*H*), 7.48 (dd, *J* = 7.8, 1.3 Hz, 1H, -Ph-*H*), 7.23 (t, *J* = 7.9 Hz, 1H, -Ph-*H*), 7.04 (dd, *J* = 8.0, 1.4 Hz, 1H, -Ph-*H*). ^{13}C NMR (125 MHz, DMSO-d_6) δ 172.21, 163.49, 158.78, 157.91, 154.02, 152.21, 143.47, 133.38, 127.95, 127.68, 127.07, 120.65, 117.71, 117.63, 111.88. HRMS (ESI): calcd. for $C_{16}H_{11}ClN_6O_3$ $[M + H]^+$ 370.0654, found 371.0659. HPLC: t_R = 1.54 min, 96.43%.

N'-(2-chloro-3-hydroxybenzylidene)-6-oxo-2-(pyridin-2-yl)-1,6-dihydropyrimidine-5-carbohydrazide (**18, A3B101**). The preparation method of compound **18** was analogous to that for **2**. Yield 84%. Yellow solid. mp > 300 °C. ^{1}H NMR (400 MHz, DMSO-d_6) δ 12.88 (bs, 1H), 10.43 (s, 1H), 8.83 (d, *J* = 4.1 Hz, 1H), 8.71 (s, 1H), 8.69 (s, 1H), 8.41 (d, *J* = 7.7 Hz, 1H), 8.11 (t, *J* = 7.2 Hz, 1H), 7.72 (dd, *J* = 7.2, 4.9 Hz, 1H), 7.47 (d, *J* = 7.3 Hz, 1H), 7.23 (t, *J* = 7.9 Hz, 1H), 7.05 (d, *J* = 7.7 Hz, 1H). ^{13}C NMR (125 MHz, DMSO-d_6) δ 161.93, 154.05, 152.33, 149.75, 144.44, 138.24, 133.17, 127.98, 126.56, 123.78, 120.79, 117.90, 117.69, 112.90. HRMS (ESI): calcd. for $C_{17}H_{12}ClN_5O_3$ $[M + H]^+$ 370.0701, found 370.0693. HPLC: t_R = 1.67 min, 100.0%.

Ethical statement

All animal-related experiments were performed in compliance with the Guide for the Care and Use of Laboratory Animals (Ministry of Science and Technology of China, 2013, IACUC-2013-012) and approved by the Institutional Animal Care and Use Committee of China Pharmaceutical University (Approval No. 20190311-1).

^{1}H NMR, HR-MS, and ^{13}C NMR spectrums of A1-A5 and the final products of 1-18.

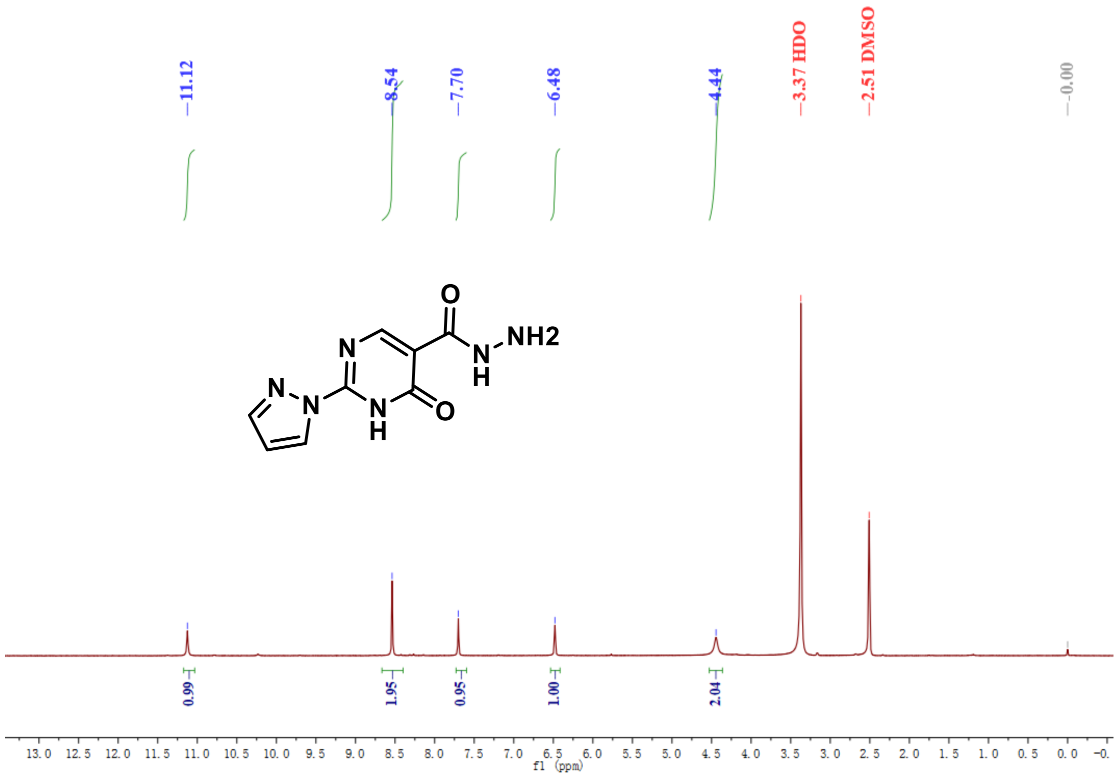

^{1}H NMR (400 MHz) of **A1** in DMSO-d_6

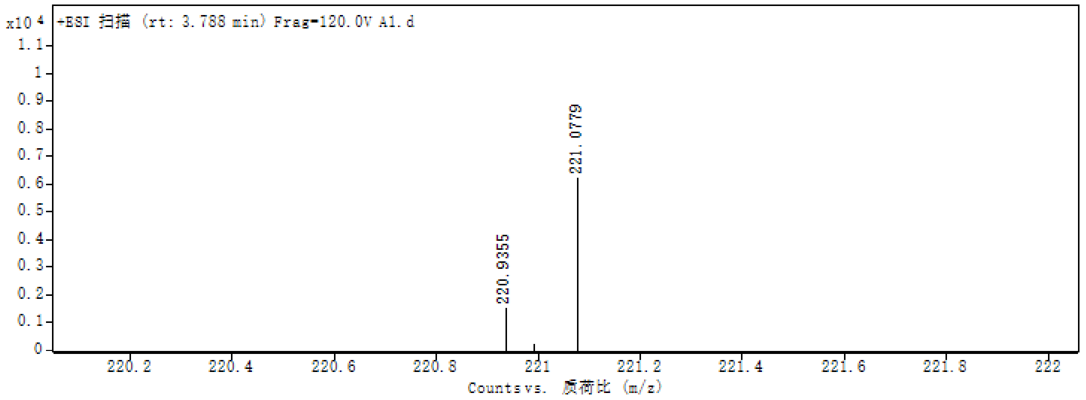

HR-MS of **A1** in MeOH

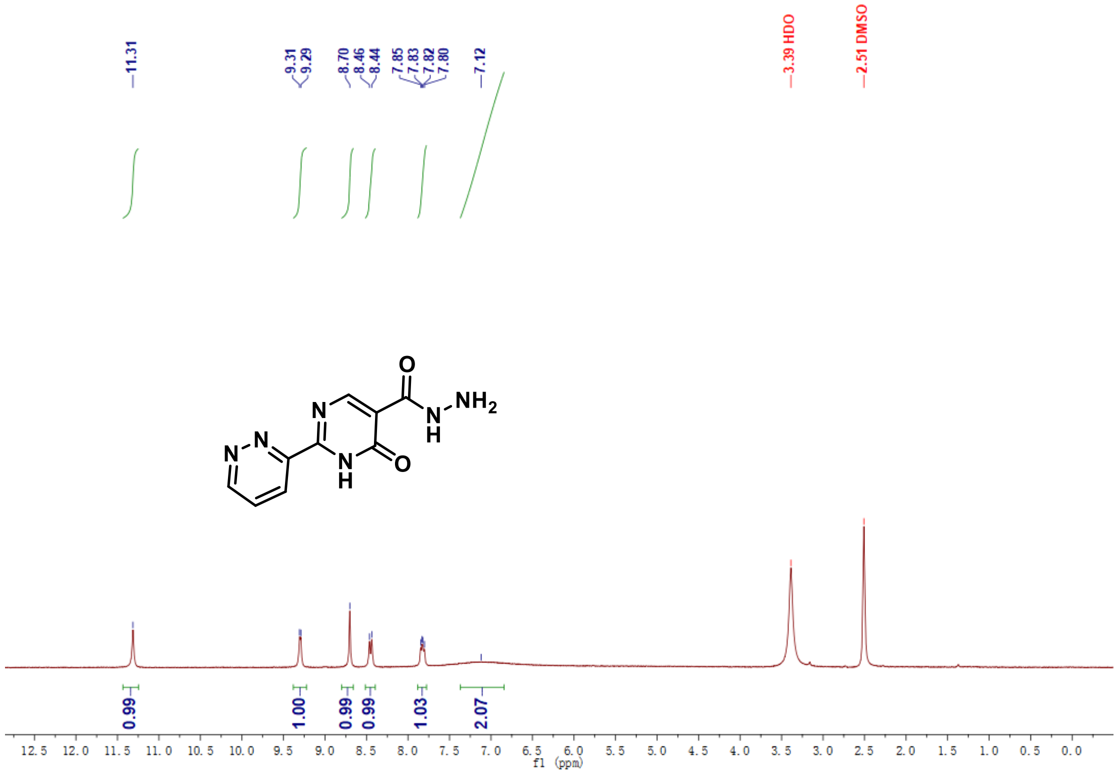

^{1}H NMR (400 MHz) of **A2** in DMSO-d_6

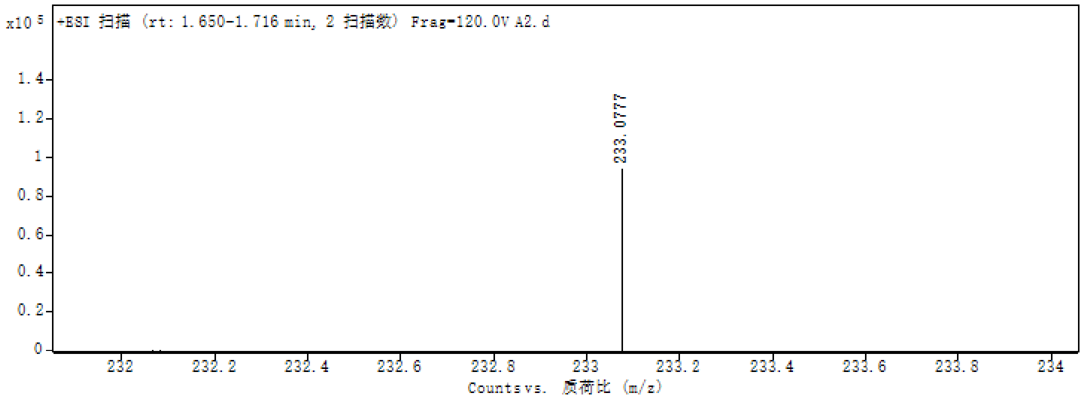

HR-MS of **A2** in MeOH

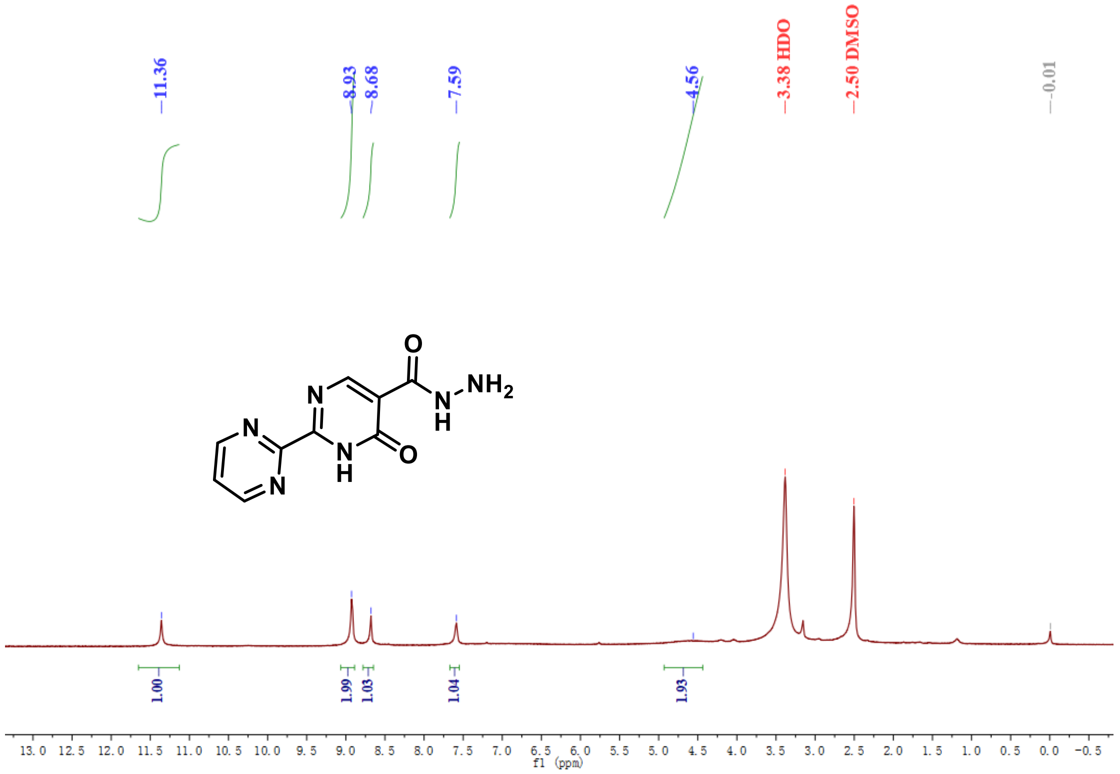

^{1}H NMR (400 MHz) of **A4** in DMSO-d_6

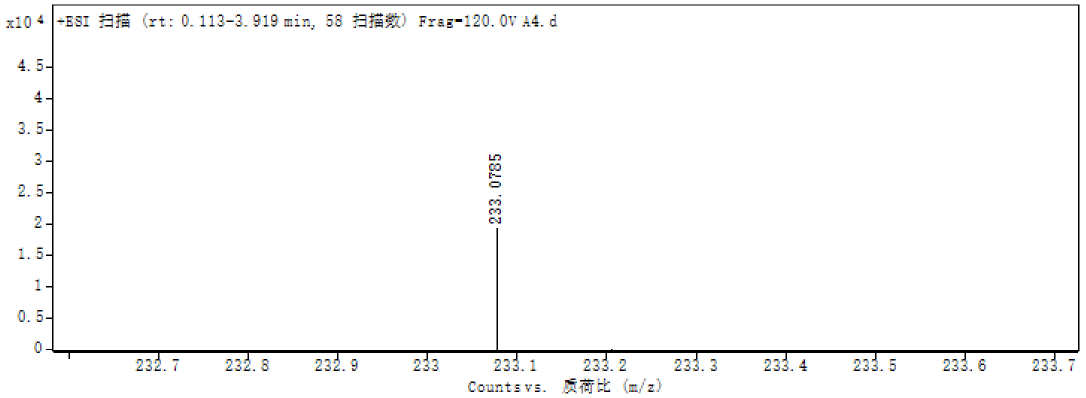

HR-MS of **A4** in MeOH

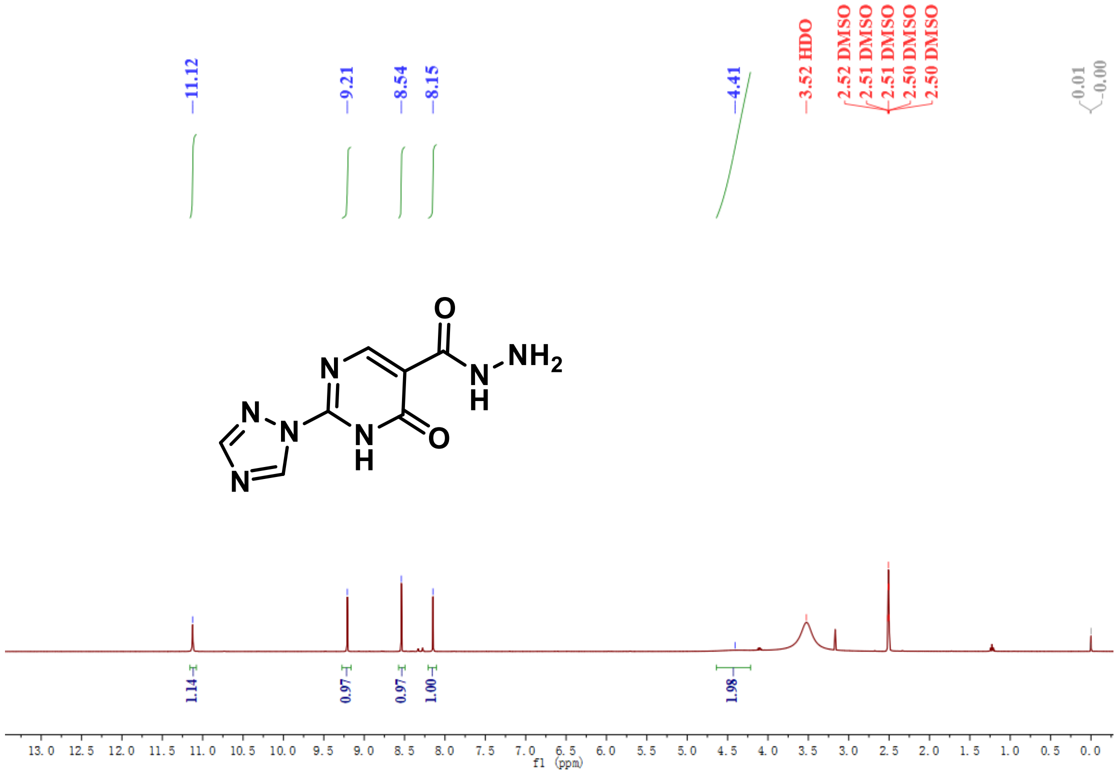

^{1}H NMR (400 MHz) of **A5** in DMSO-d_6

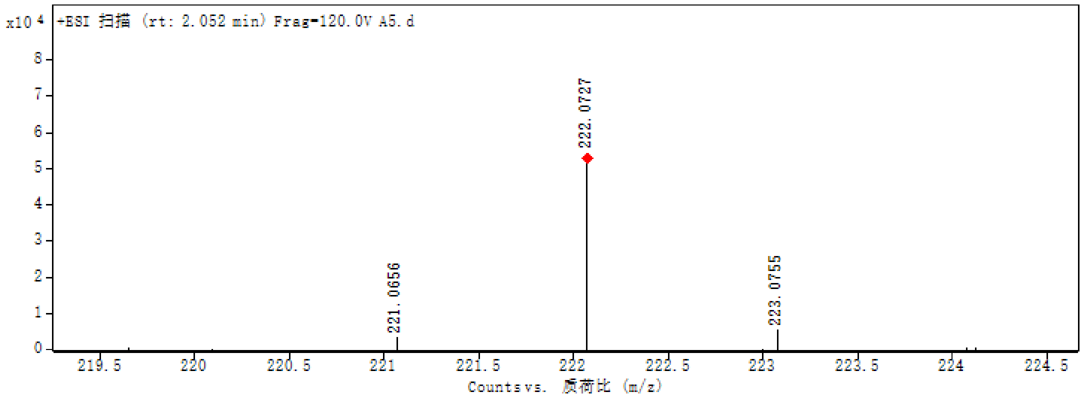

HR-MS of **A5** in MeOH

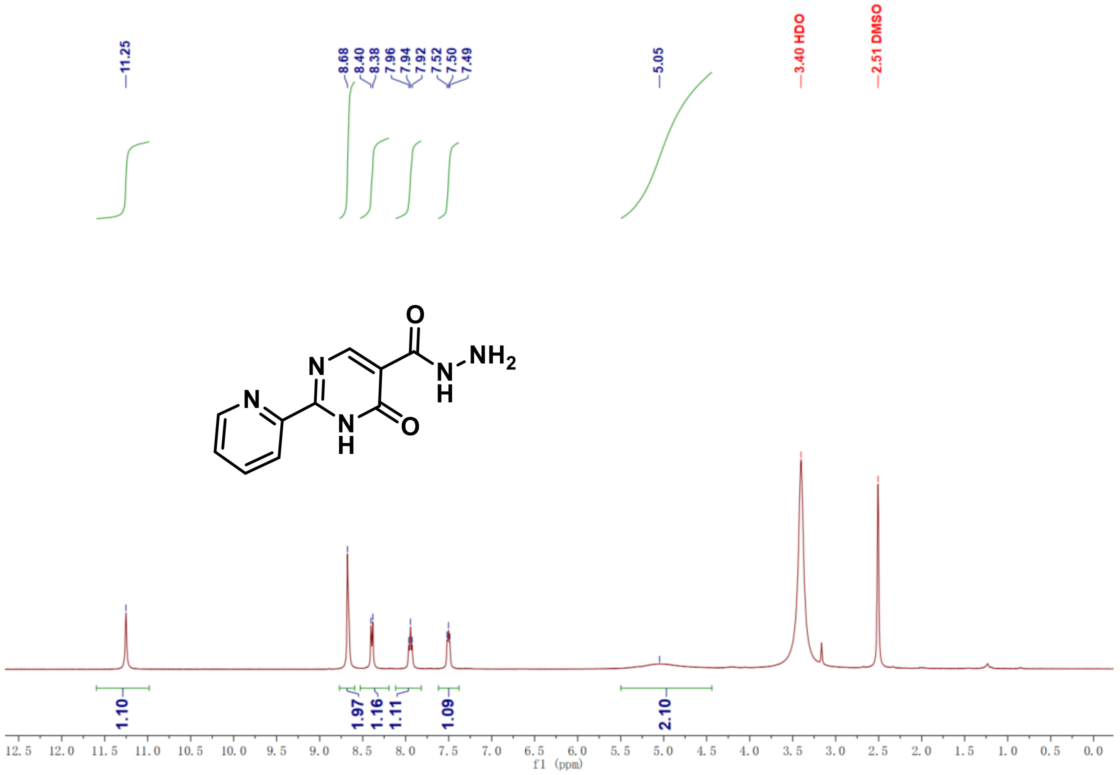

^{1}H NMR (400 MHz) of **A3** in DMSO-d_6

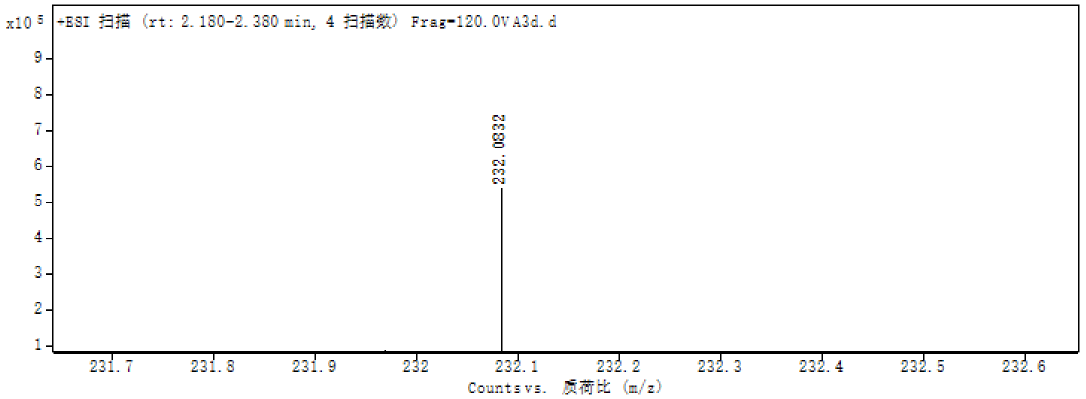

HR-MS of **A3** in MeOH

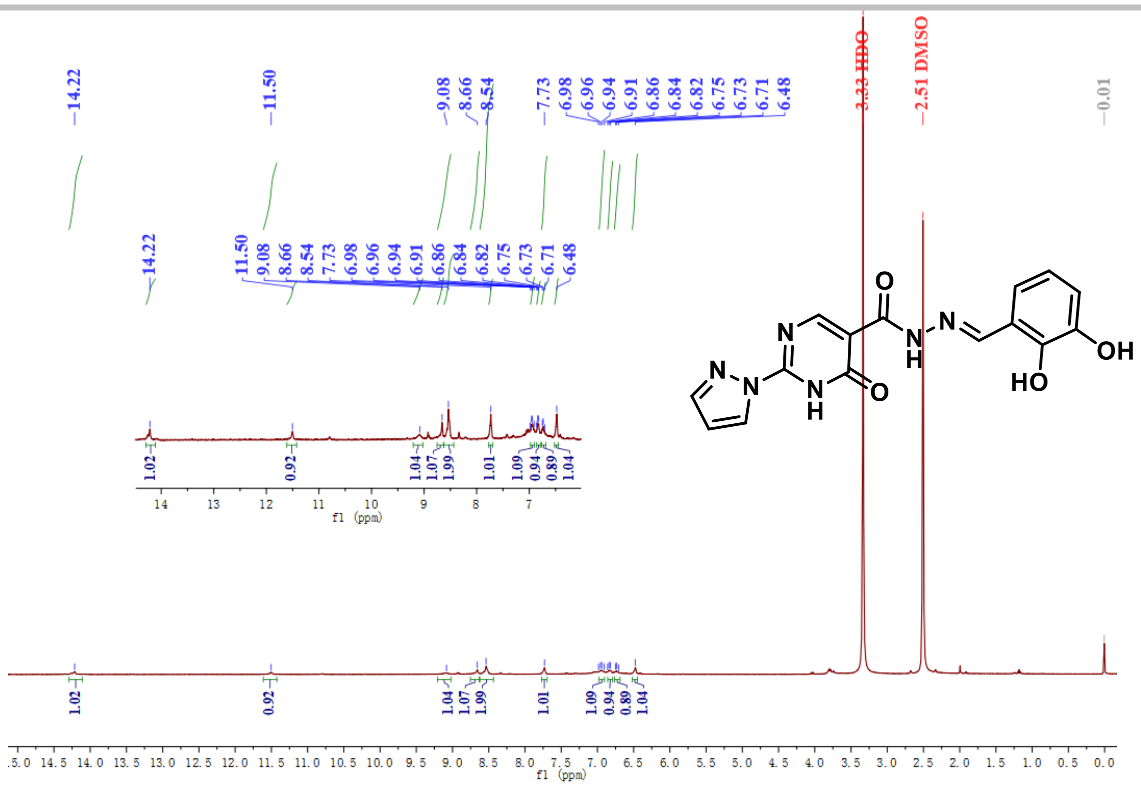

^{1}H NMR (400 MHz) of **1** in DMSO-d_6

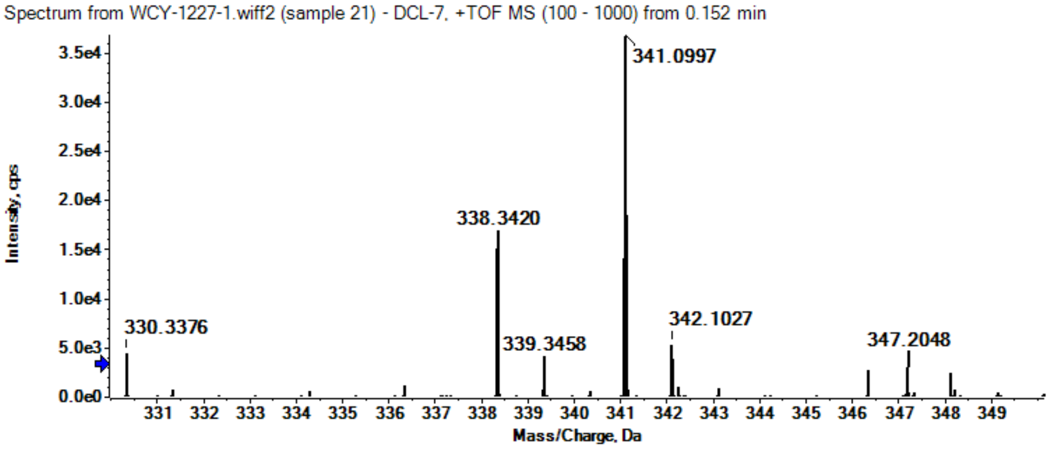

HR-MS of **1** in MeOH

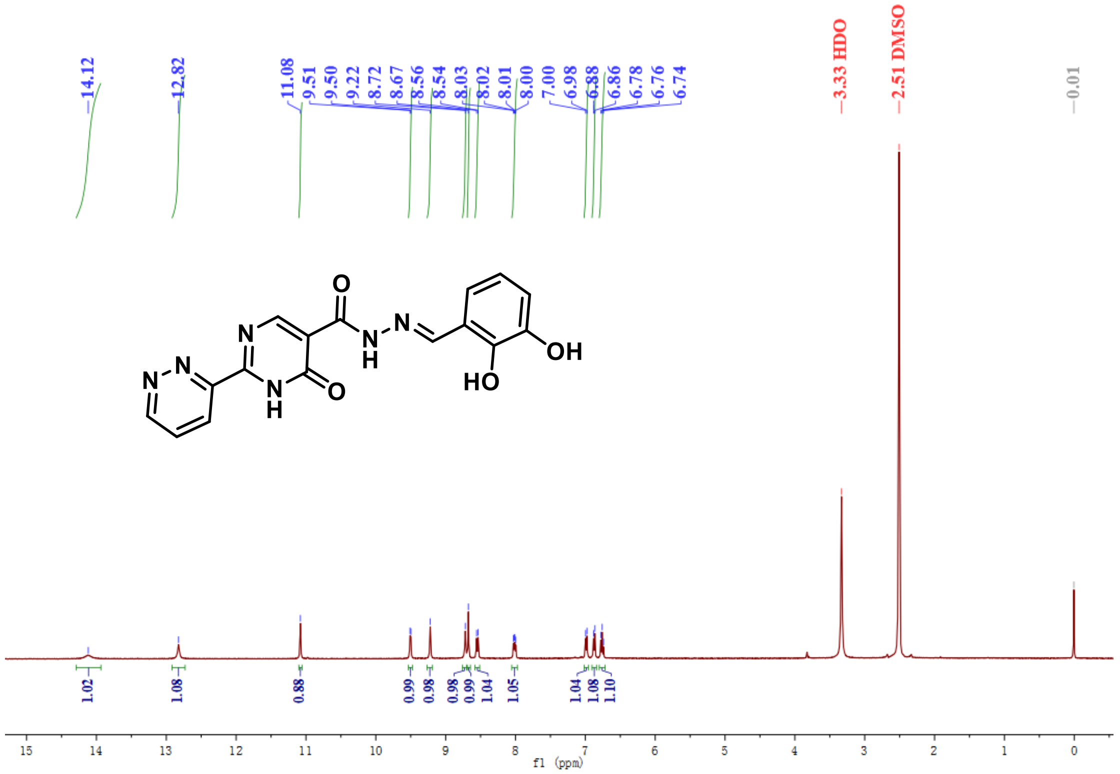

^{1}H NMR (400 MHz) of **2** in DMSO-d_6

Monoisotopic Mass, Even Electron Ions
27 formula(e) evaluated with 0 results within limits (up to 50 best isotopic matches for each mass)
Elements Used:
C: 20-22 H: 20-22 N: 2-4 O: 2-4 Cl: 2-4
DCL-8 372 (2.673)
1: TOF MS ES+

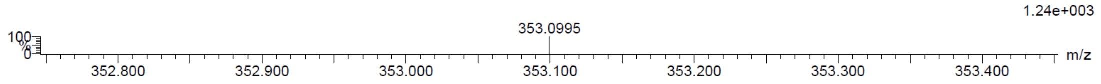

	Mass	Calc. Mass	mDa	PPM	DBE	i-FIT	Norm	Conf(%)	Formula
Minimum:					-1.5				
Maximum:			5.0	10.0	50.0				
	353.0995	---							

HR-MS of **2** in MeOH

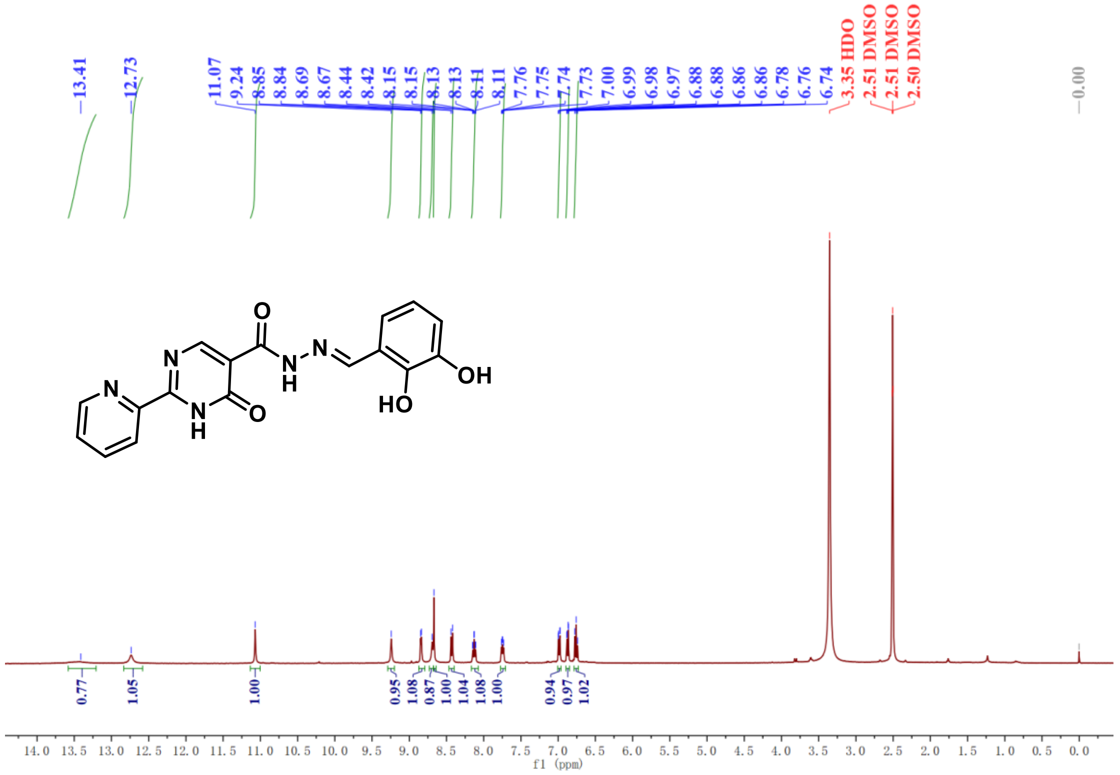

^{1}H NMR (400 MHz) of **3** in DMSO-d_6

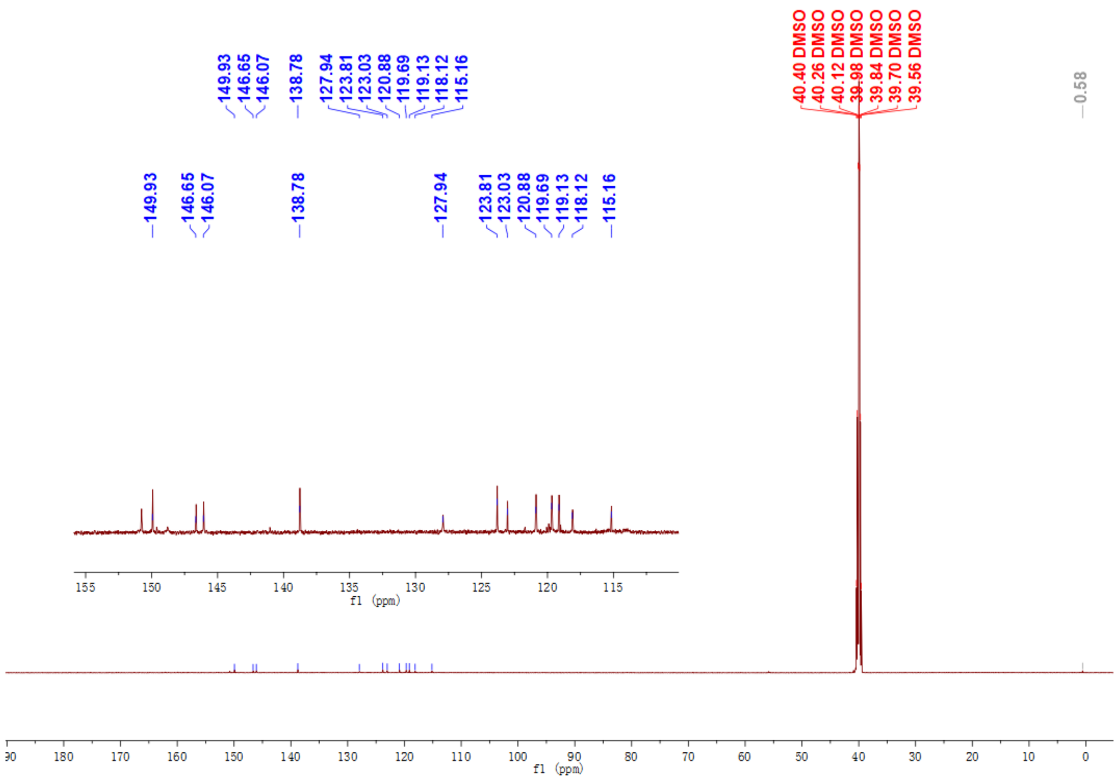

^{13}C NMR (125 MHz) of **3** in DMSO-d_6

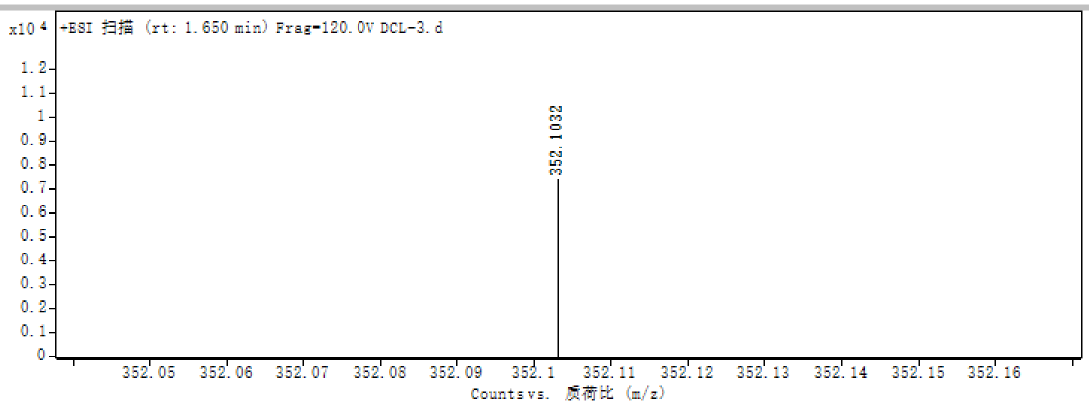

HR-MS of **3** in MeOH

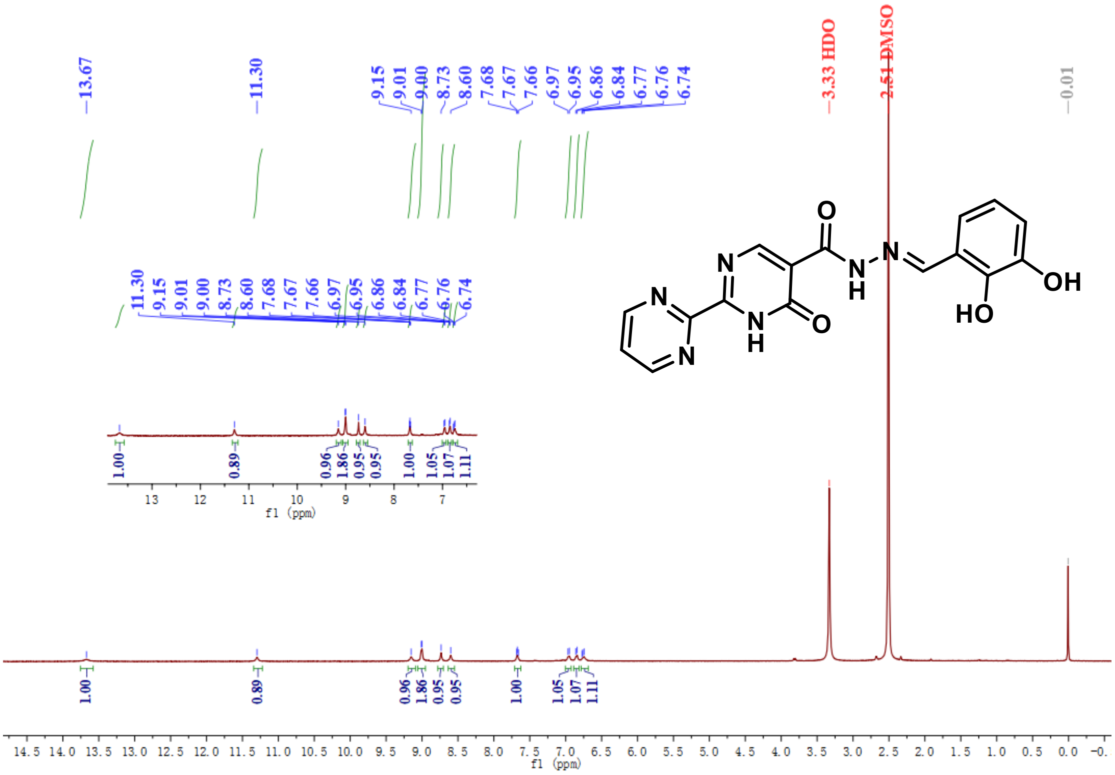

^{1}H NMR (400 MHz) of **4** in DMSO-d_6

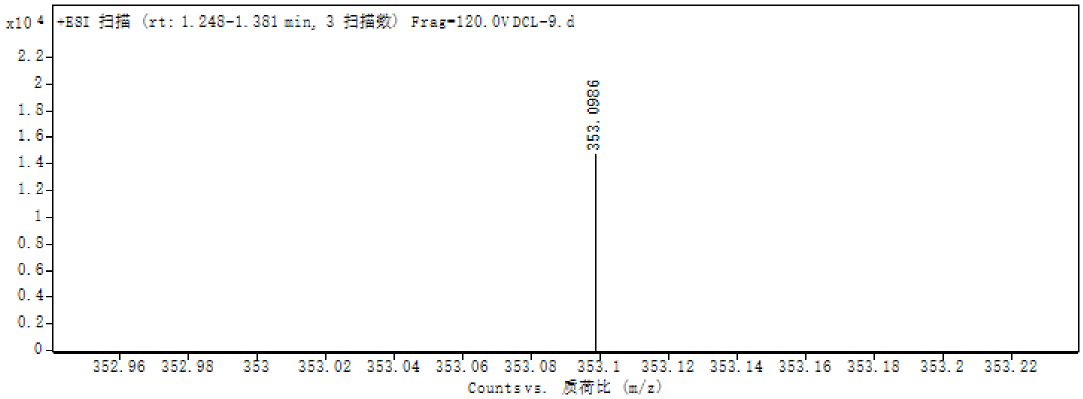

HR-MS of **4** in MeOH

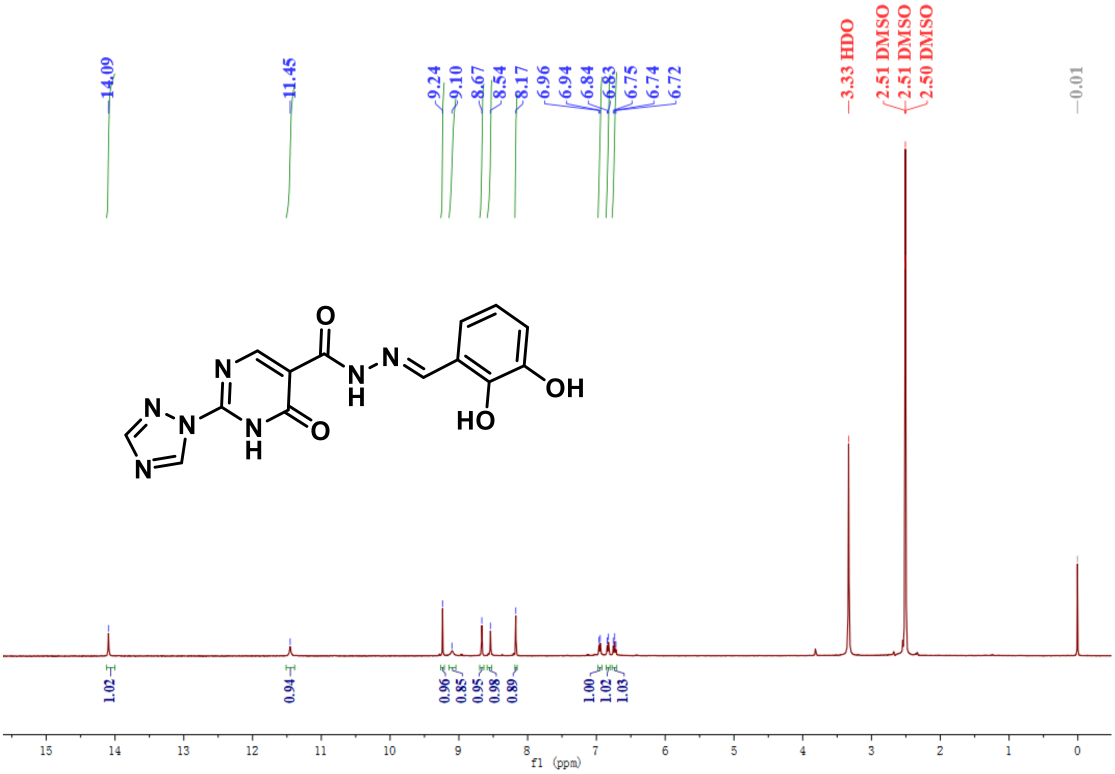

^{1}H NMR (400 MHz) of **5** in DMSO-d_6

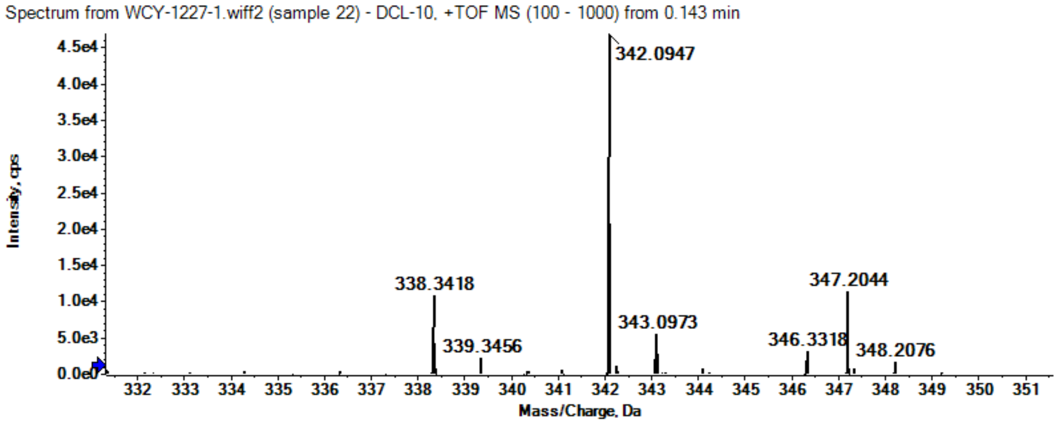

HR-MS of **5** in MeOH

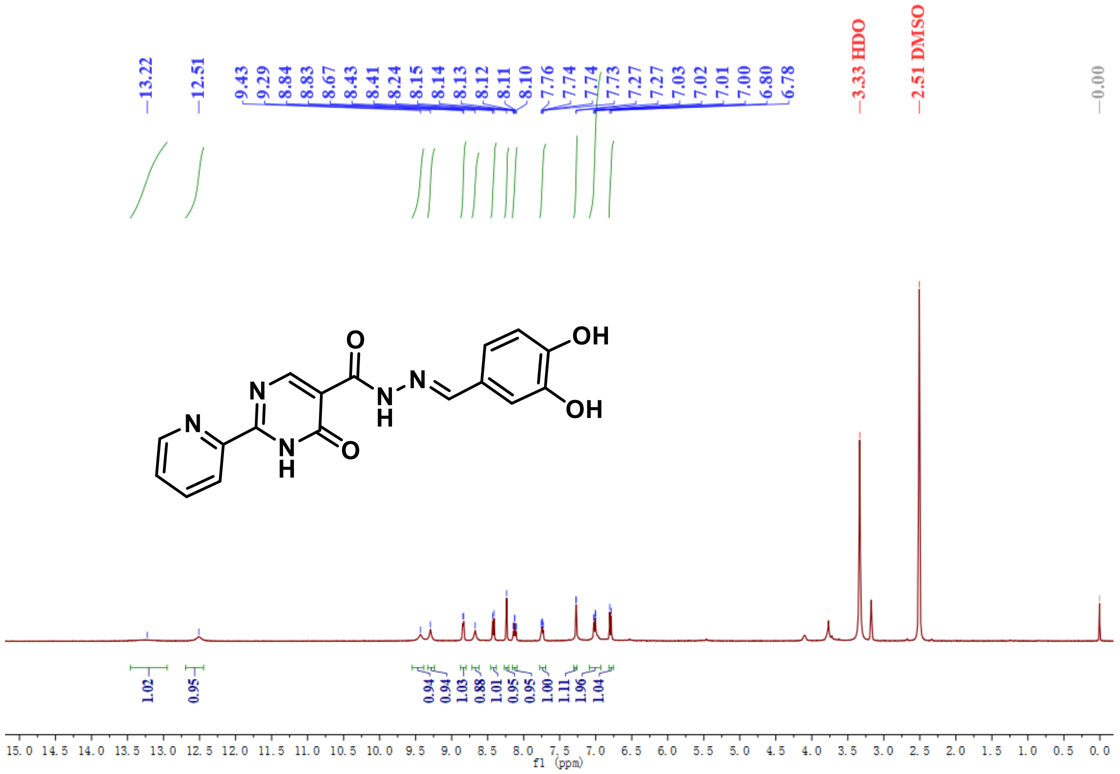

^{1}H NMR (400 MHz) of **6** in DMSO-d_6

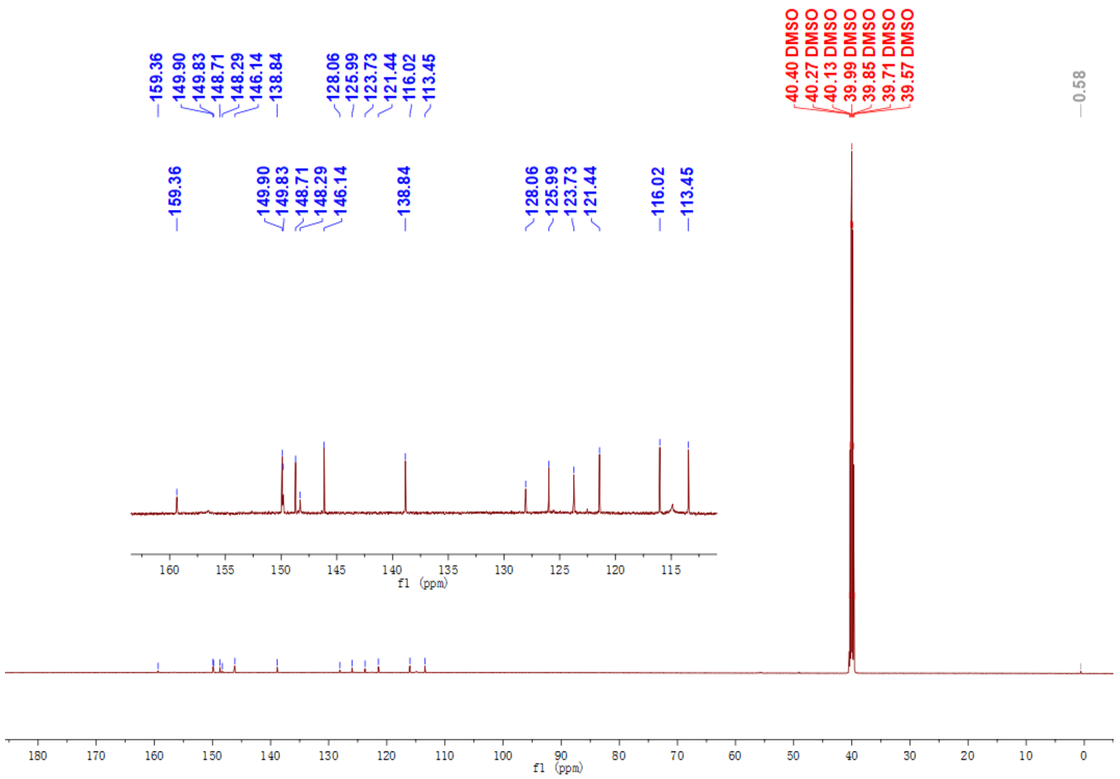

^{13}C NMR (125 MHz) of **6** in DMSO-d_6

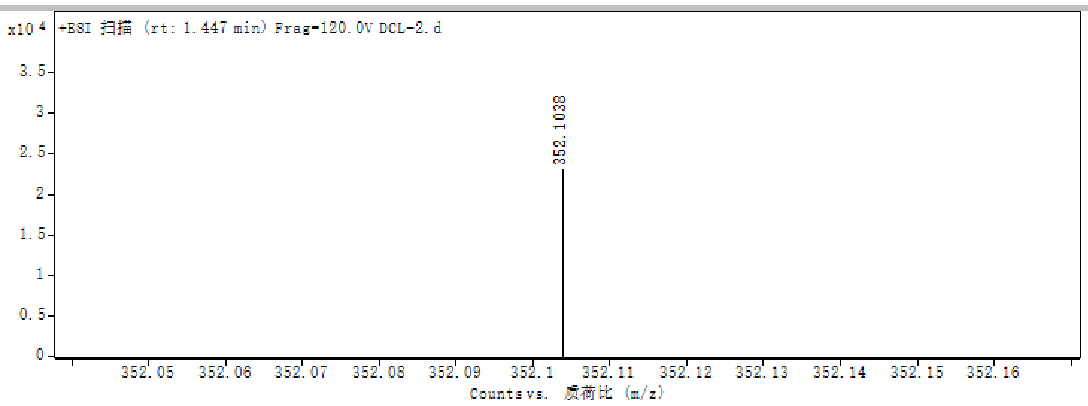

HR-MS of **6** in MeOH

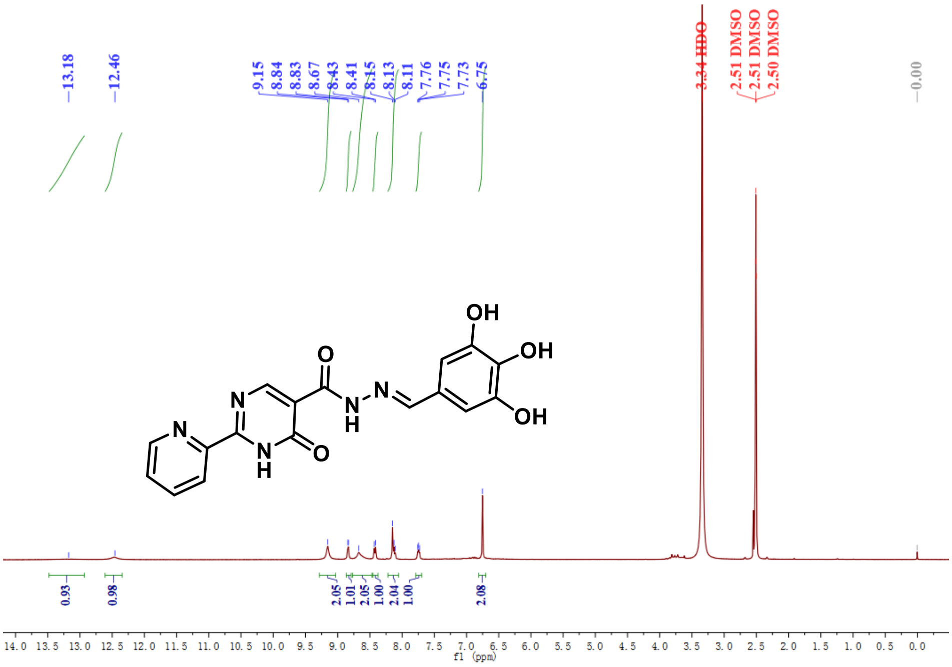

^{1}H NMR (400 MHz) of **7** in DMSO-d_6

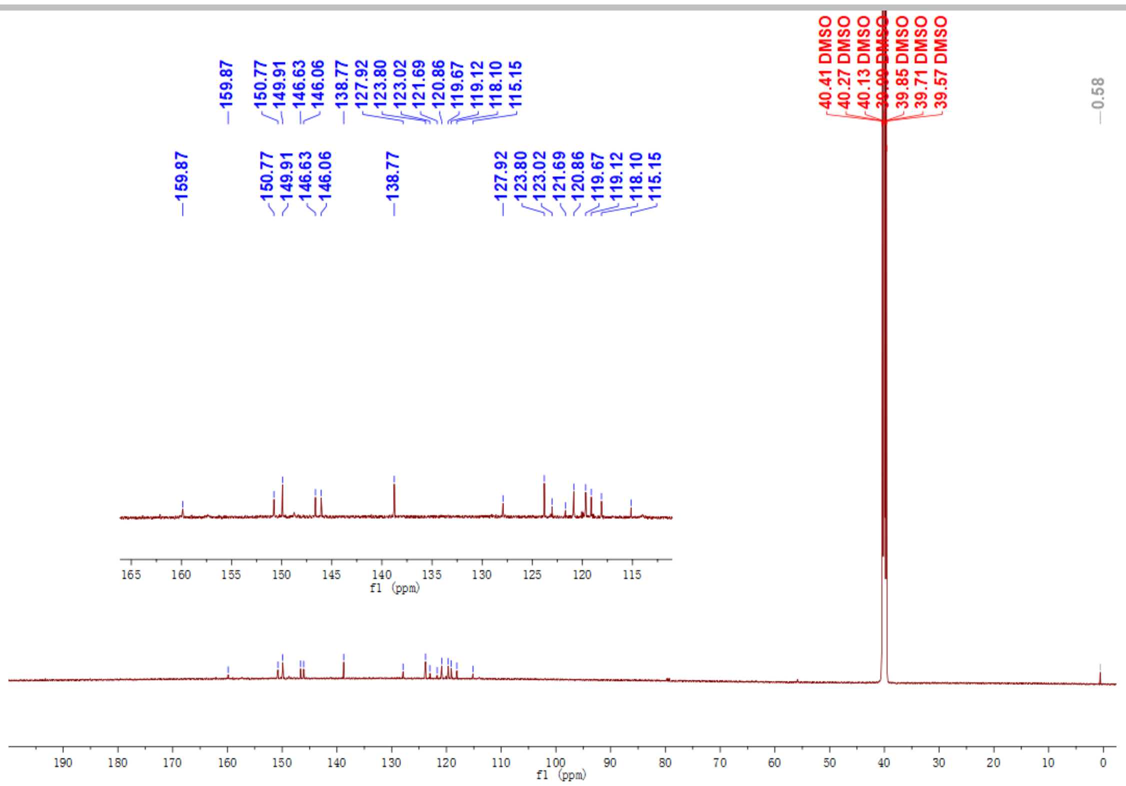

^{13}C NMR (125 MHz) of **7** in DMSO-d_6

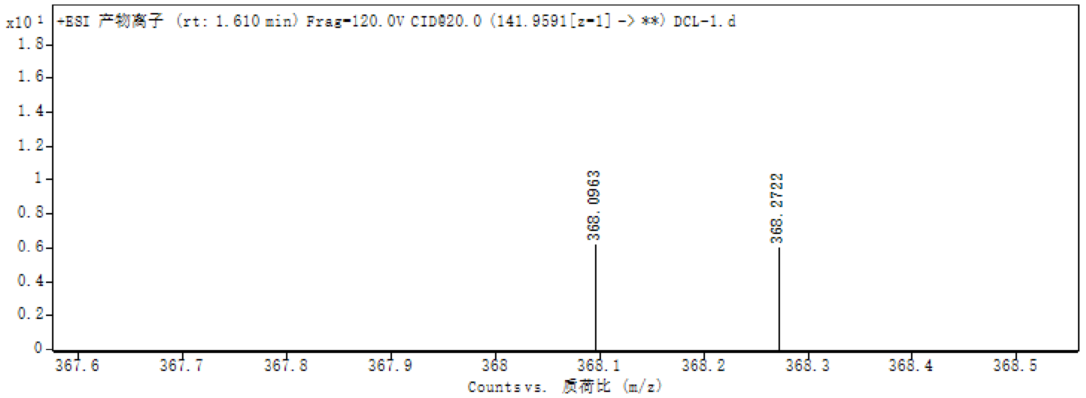

HR-MS of **7** in MeOH

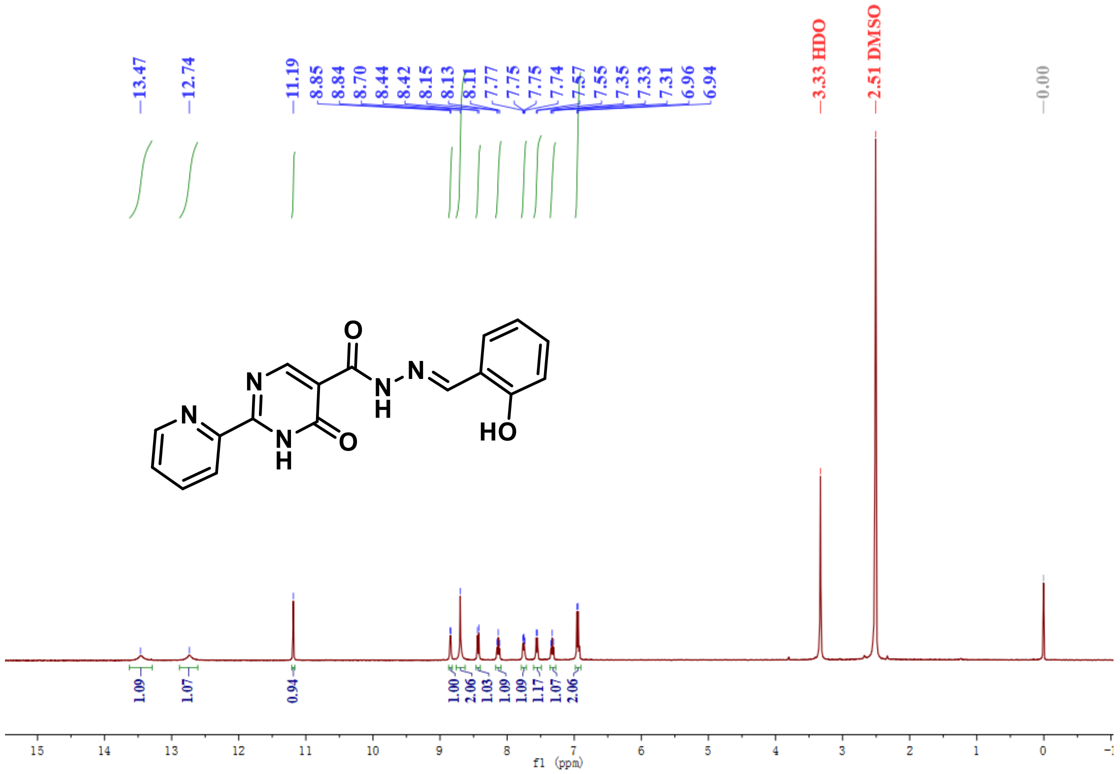

^{1}H NMR (400 MHz) of **8** in DMSO-d_6

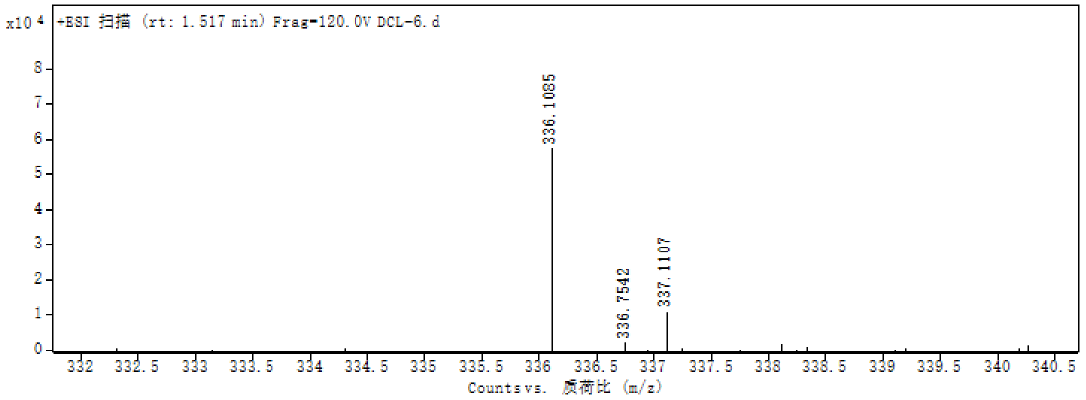

HR-MS of **8** in MeOH

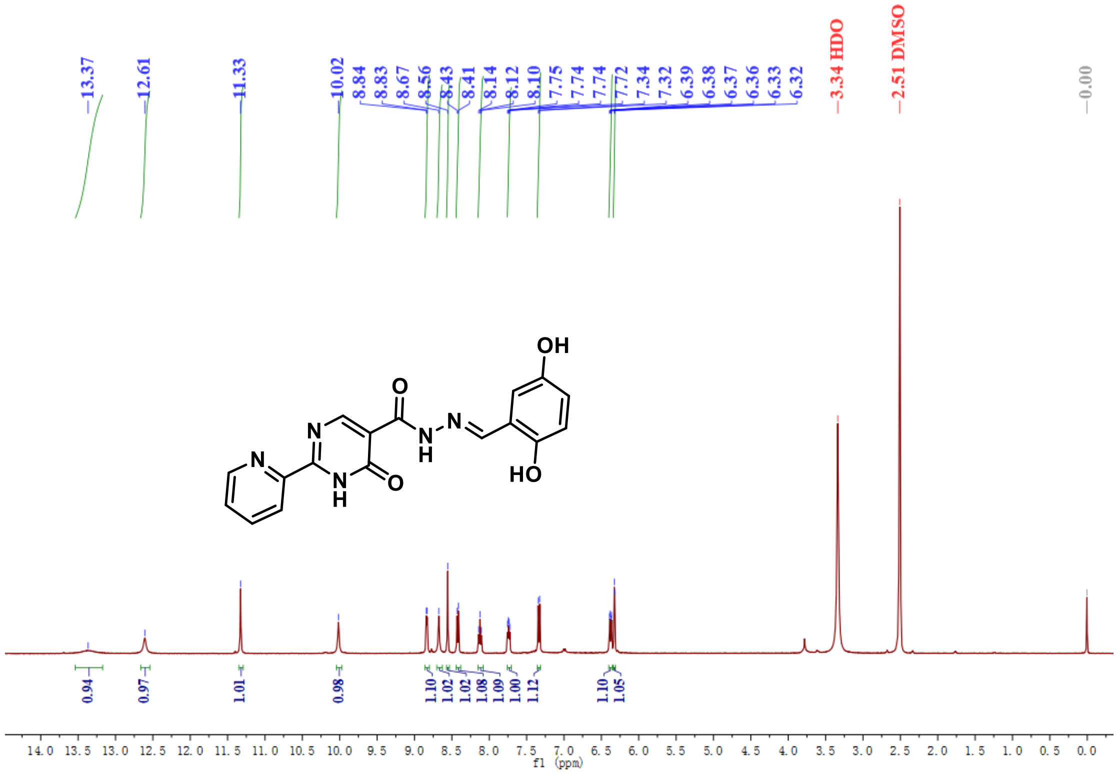

^{1}H NMR (400 MHz) of **9** in DMSO-d_6

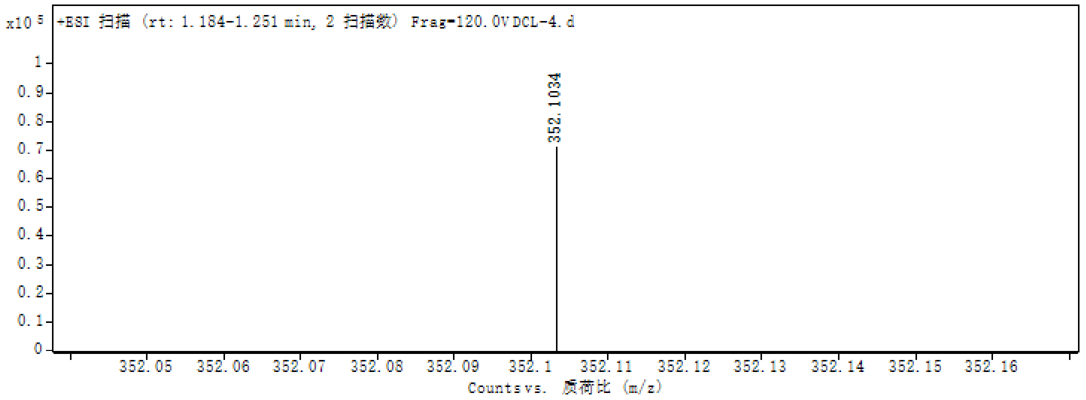

HR-MS of **9** in MeOH

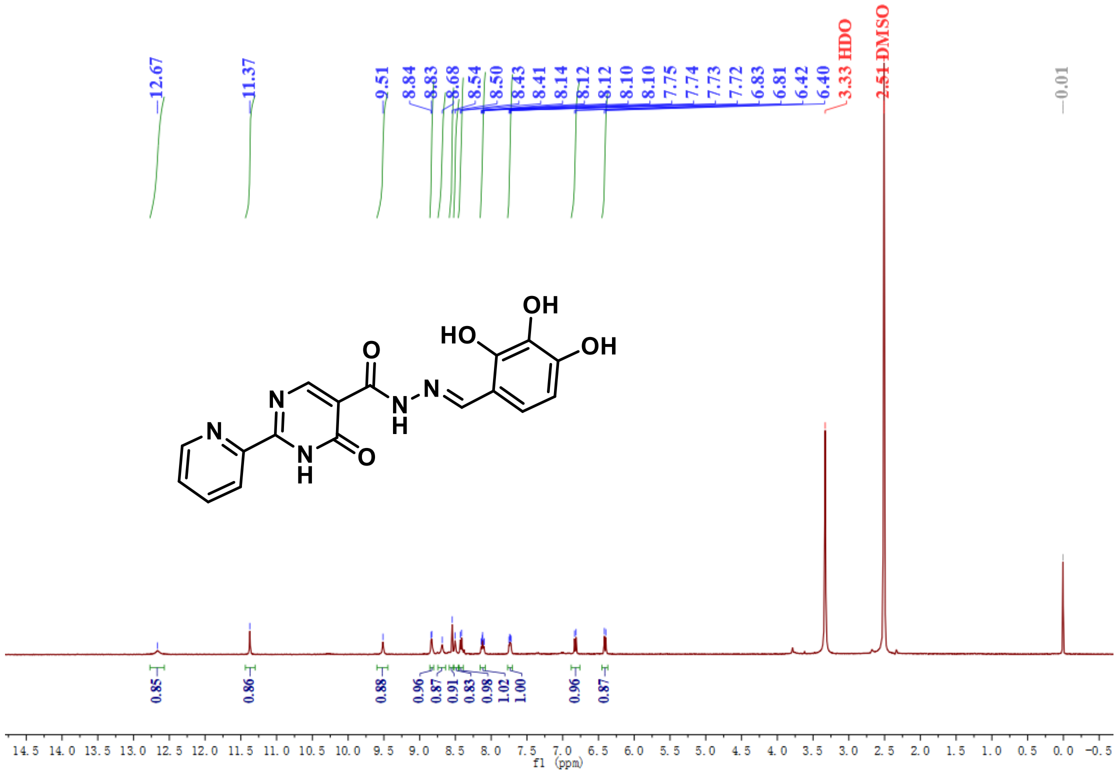

^{1}H NMR (400 MHz) of **10** in DMSO-d_6

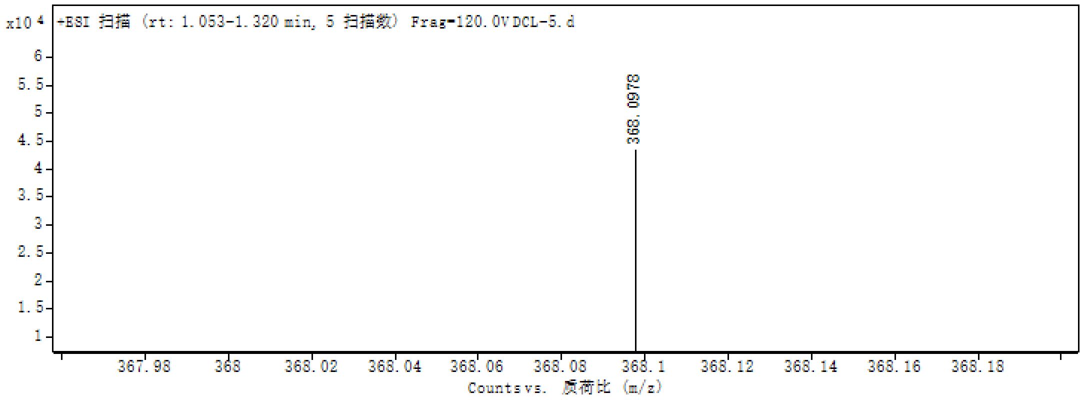

HR-MS of **10** in MeOH

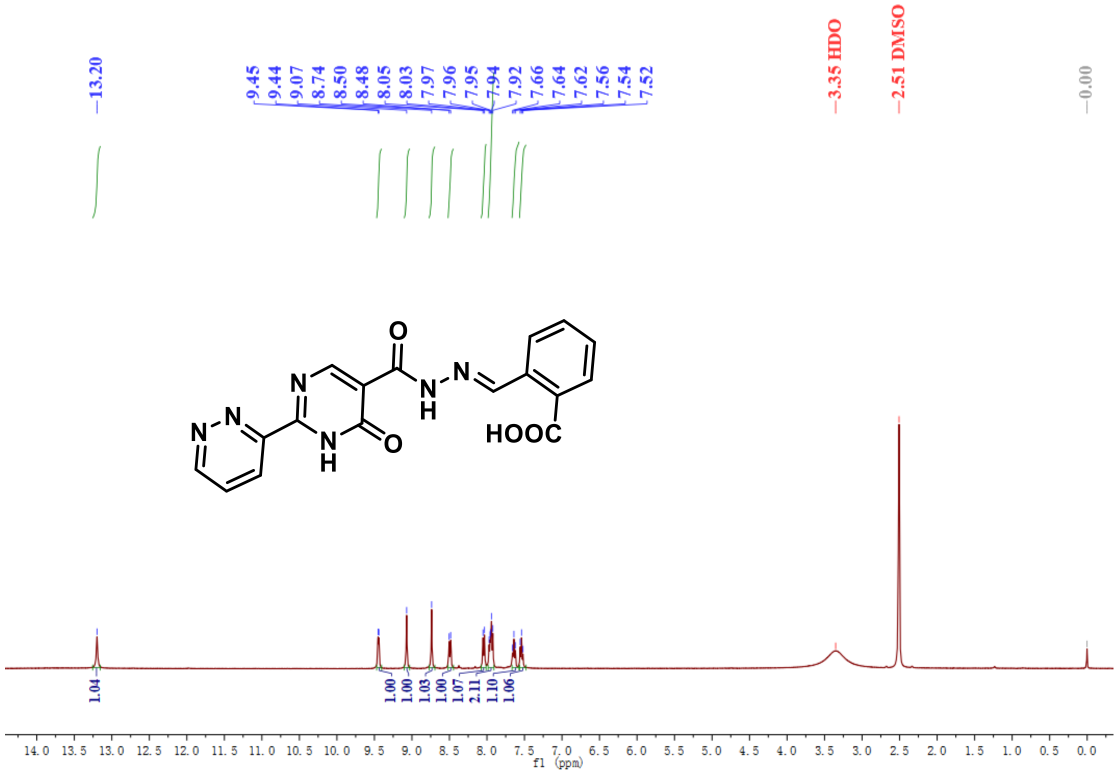

^{1}H NMR (400 MHz) of **11** in DMSO-d_6

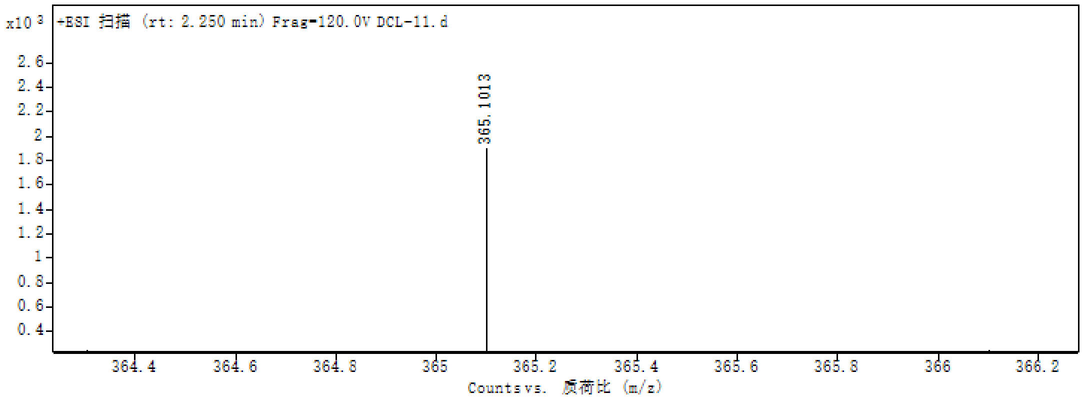

HR-MS of **11** in MeOH

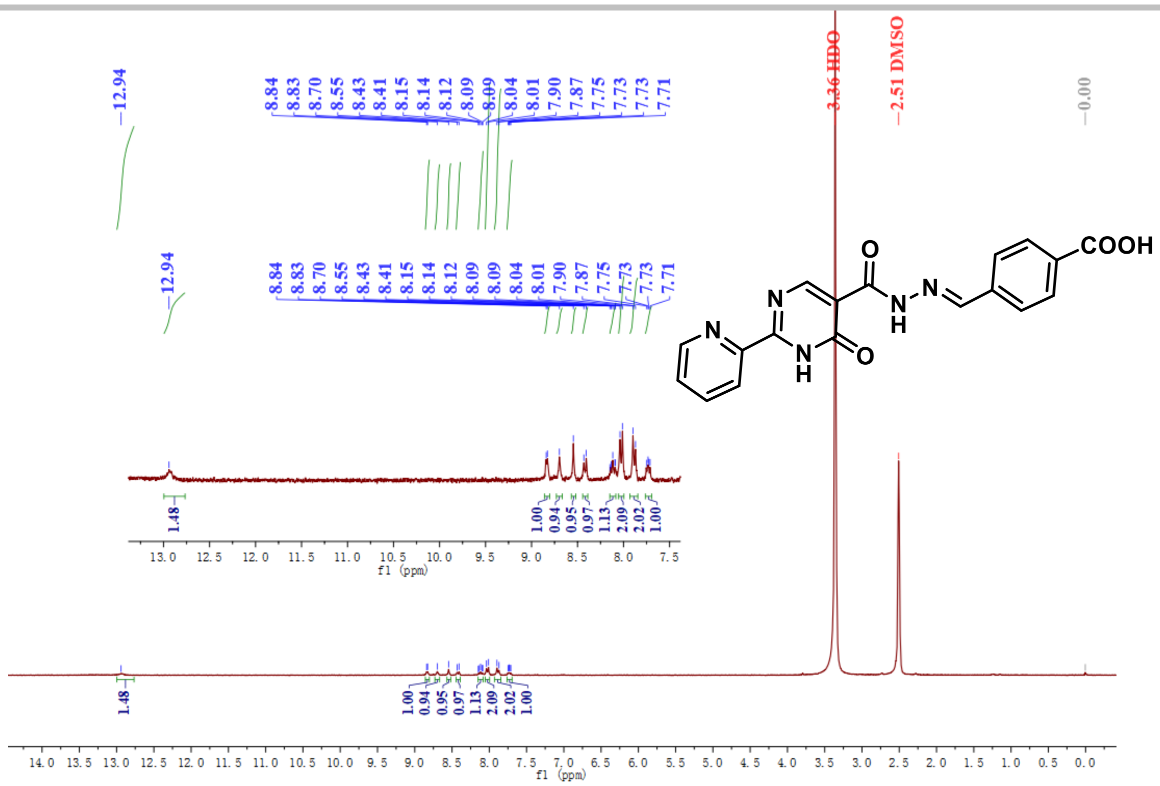

^{1}H NMR (400 MHz) of **12** in DMSO-d_6

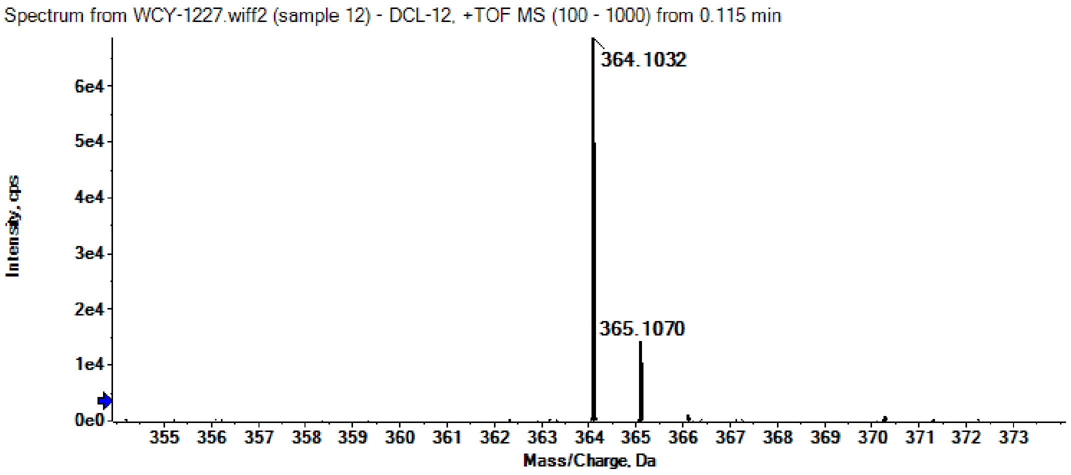

HR-MS of **12** in MeOH

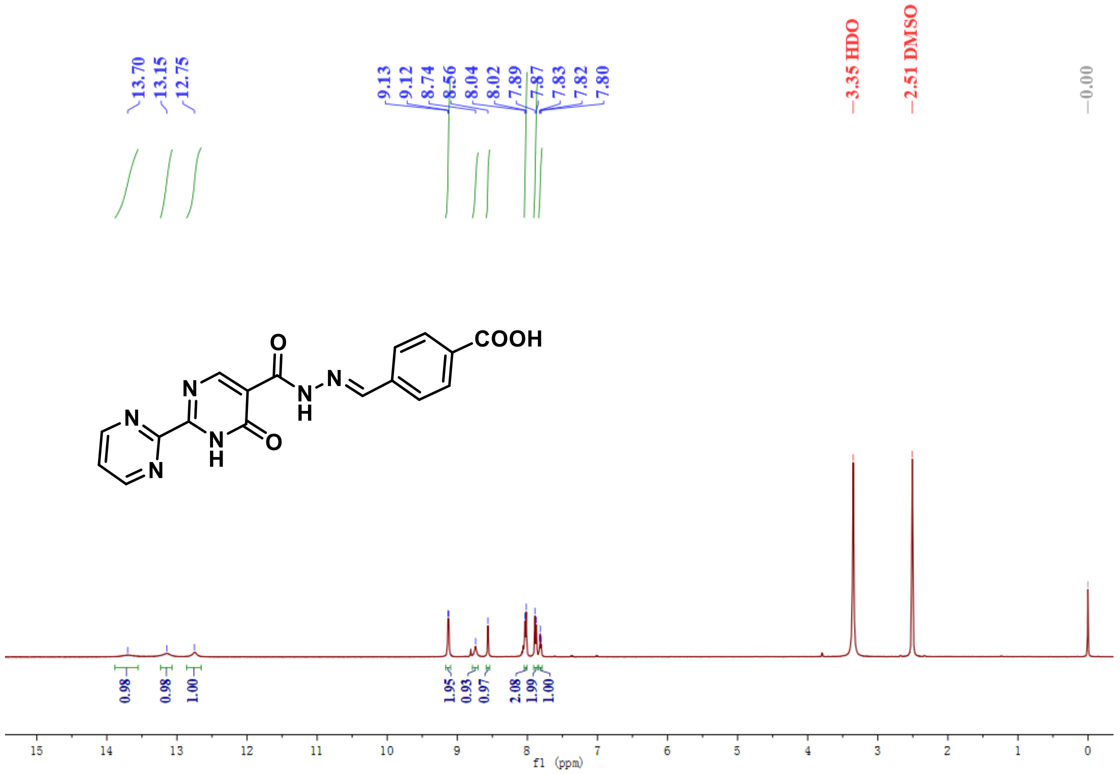

^{1}H NMR (400 MHz) of **13** in DMSO-d_6

Monoisotopic Mass, Even Electron Ions
26 formula(e) evaluated with 0 results within limits (up to 50 best isotopic matches for each mass)
Elements Used:
C: 20-22 H: 20-22 N: 2-4 O: 2-4 Cl: 2-4
DCL-13 513 (3.678)
1: TOF MS ES+

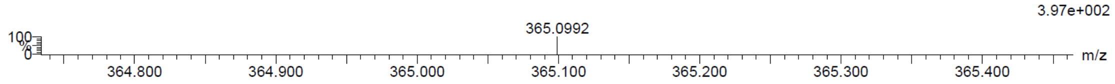

Minimum:				-1.5				
Maximum:		5.0	10.0	50.0				
Mass	Calc. Mass	mDa	PPM	DBE	i-FIT	Norm	Conf(%)	Formula
365.0992	---							

HR-MS of **13** in MeOH

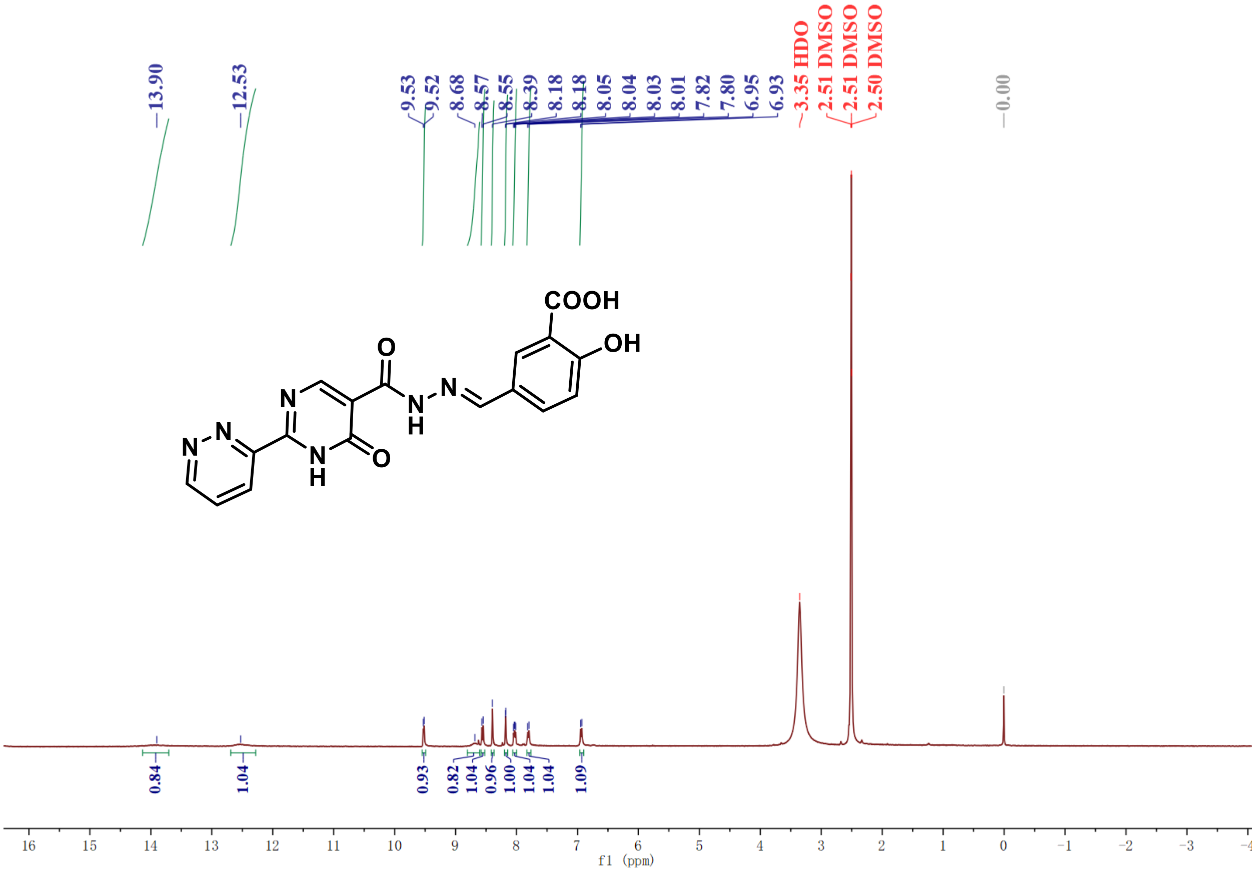

^{1}H NMR (400 MHz) of **14** in DMSO-d_6

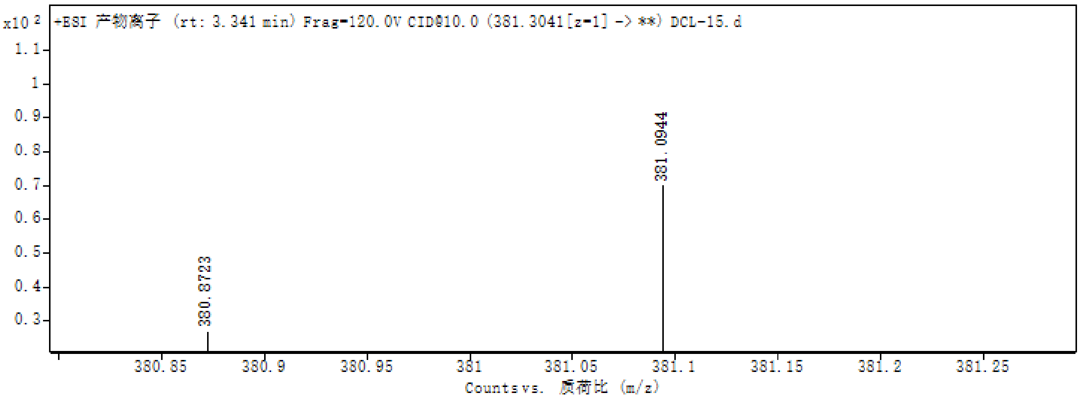

HR-MS of **14** in MeOH

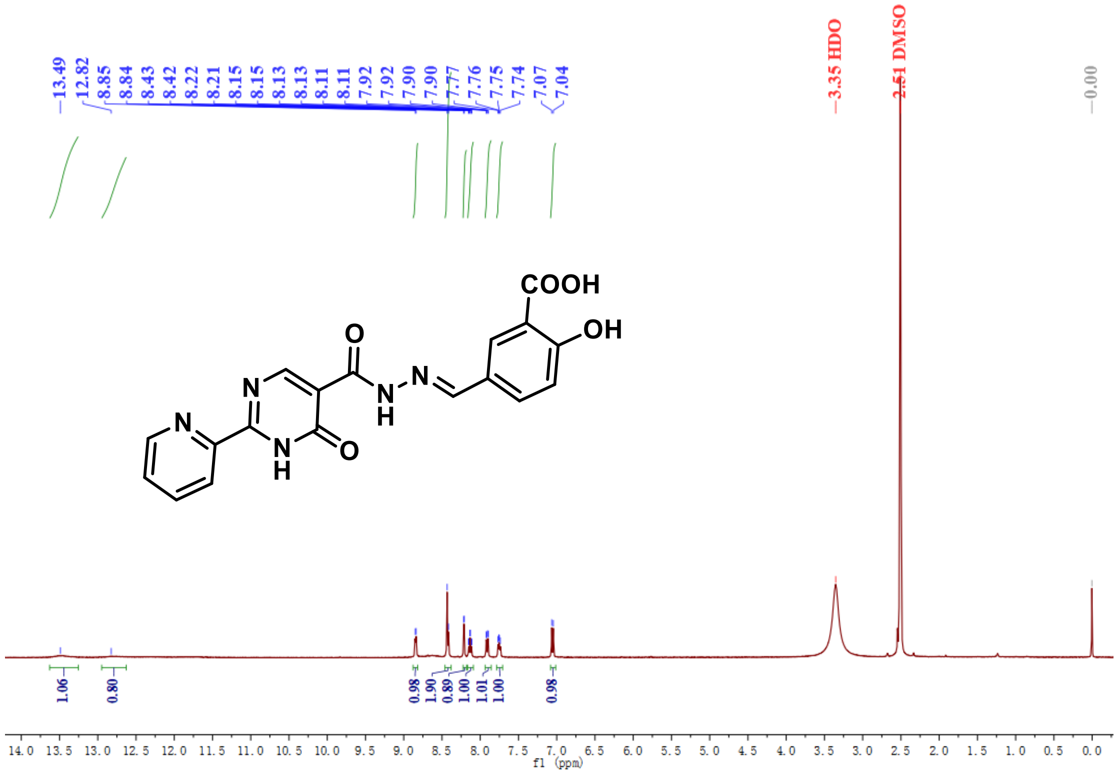

^{1}H NMR (400 MHz) of **15** in DMSO-d_6

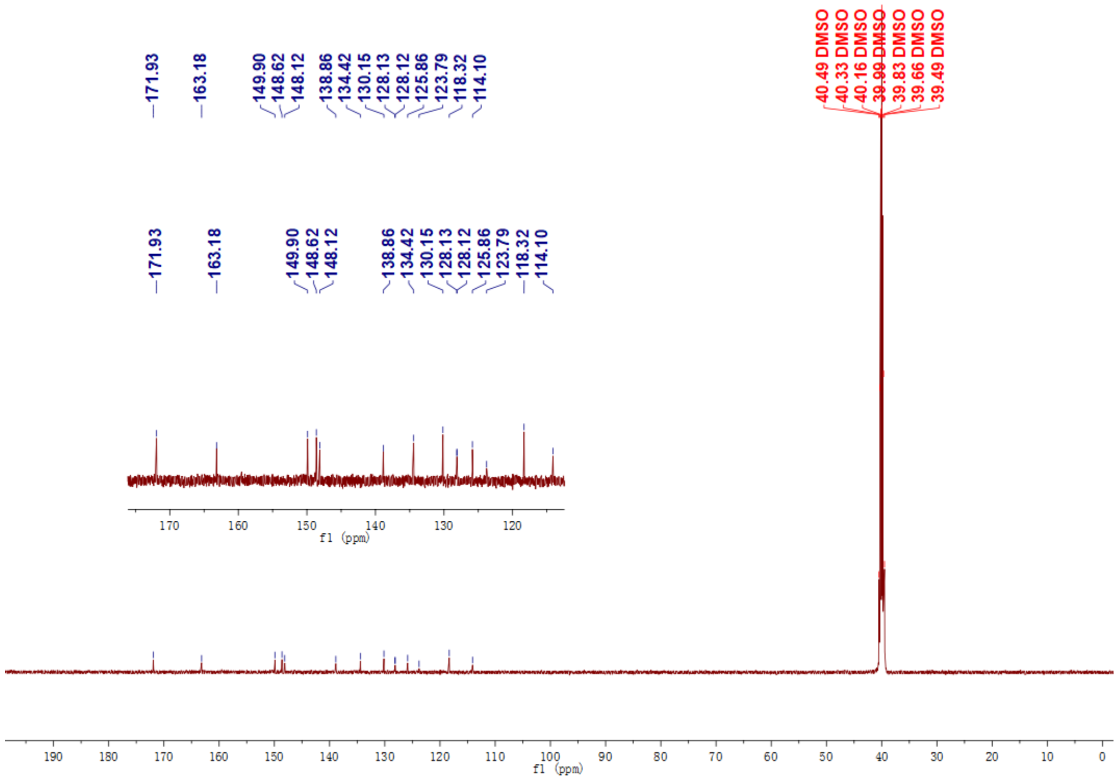

^{13}C NMR (125 MHz) of **15** in DMSO-d_6

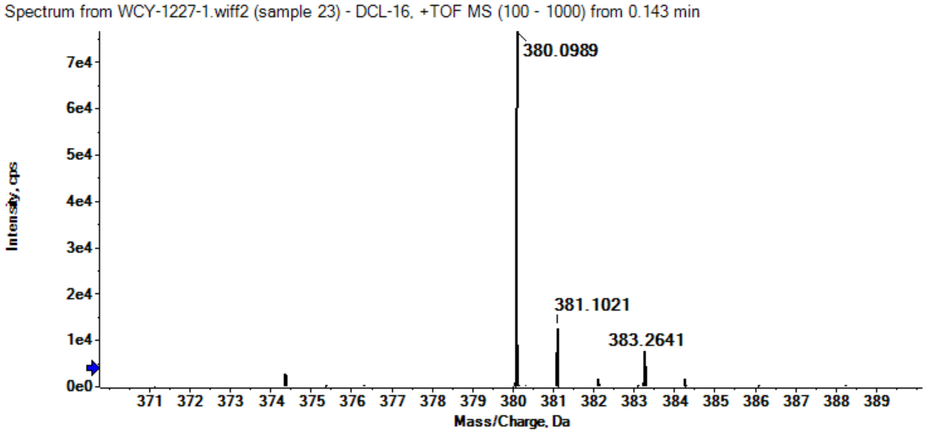

HR-MS of **15** in MeOH

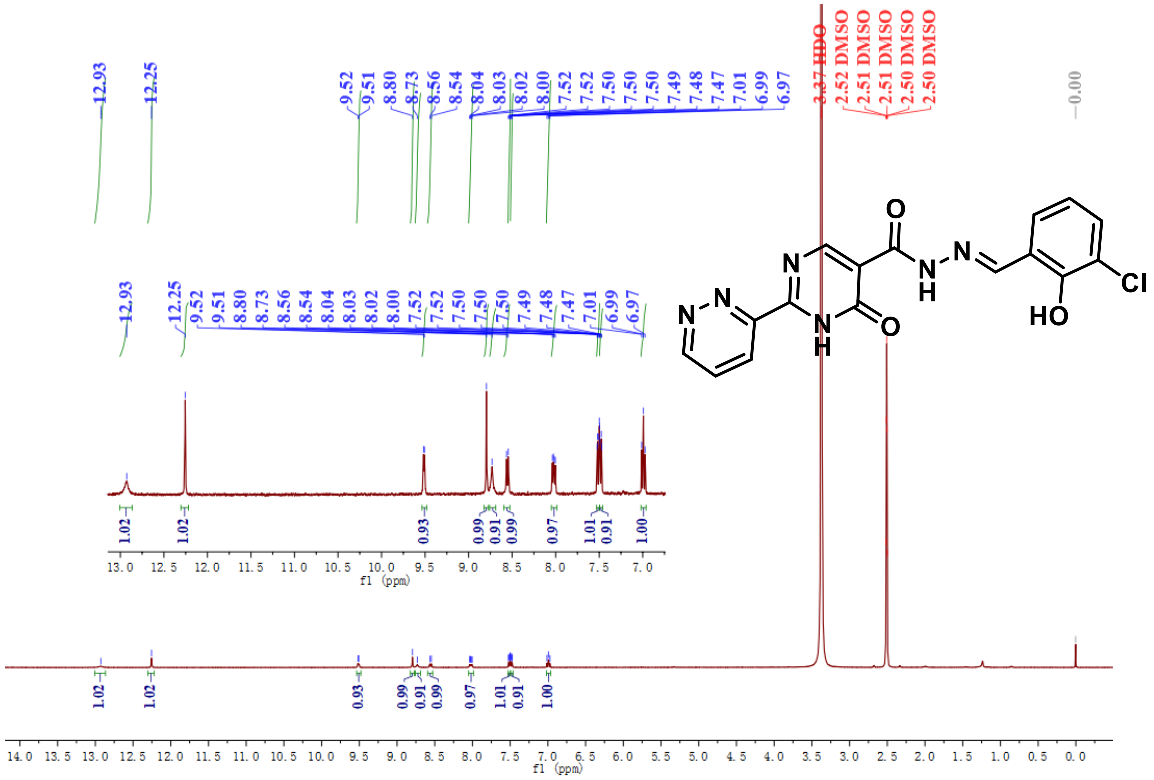

^{1}H NMR (400 MHz) of **16** in DMSO-d_6

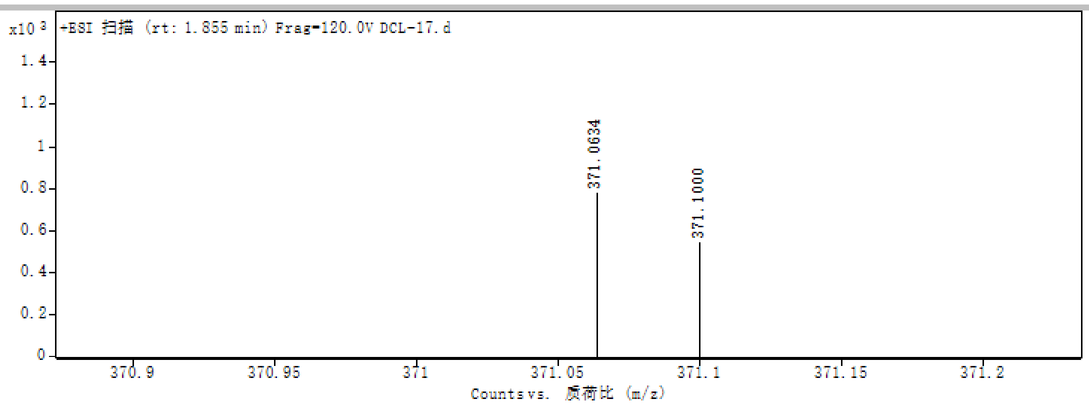

HR-MS of **16** in MeOH

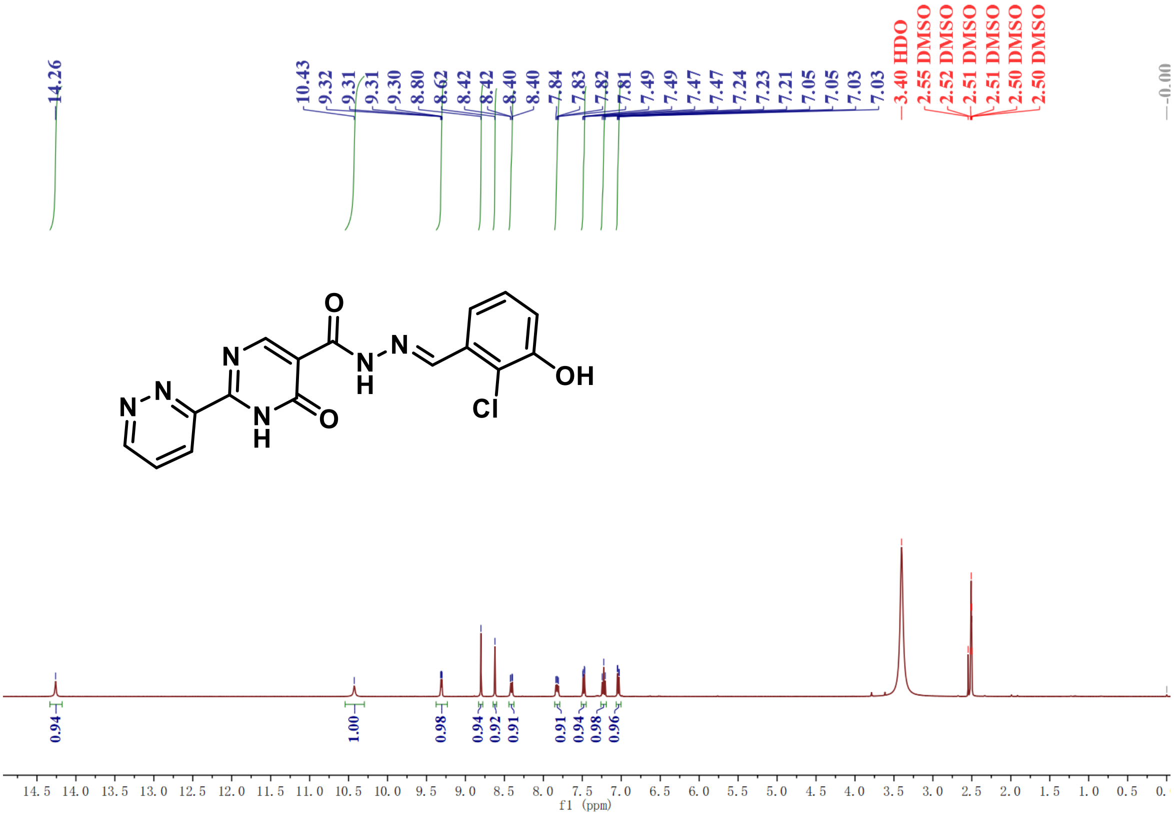

^{1}H NMR (400 MHz) of **17** in DMSO-d_6

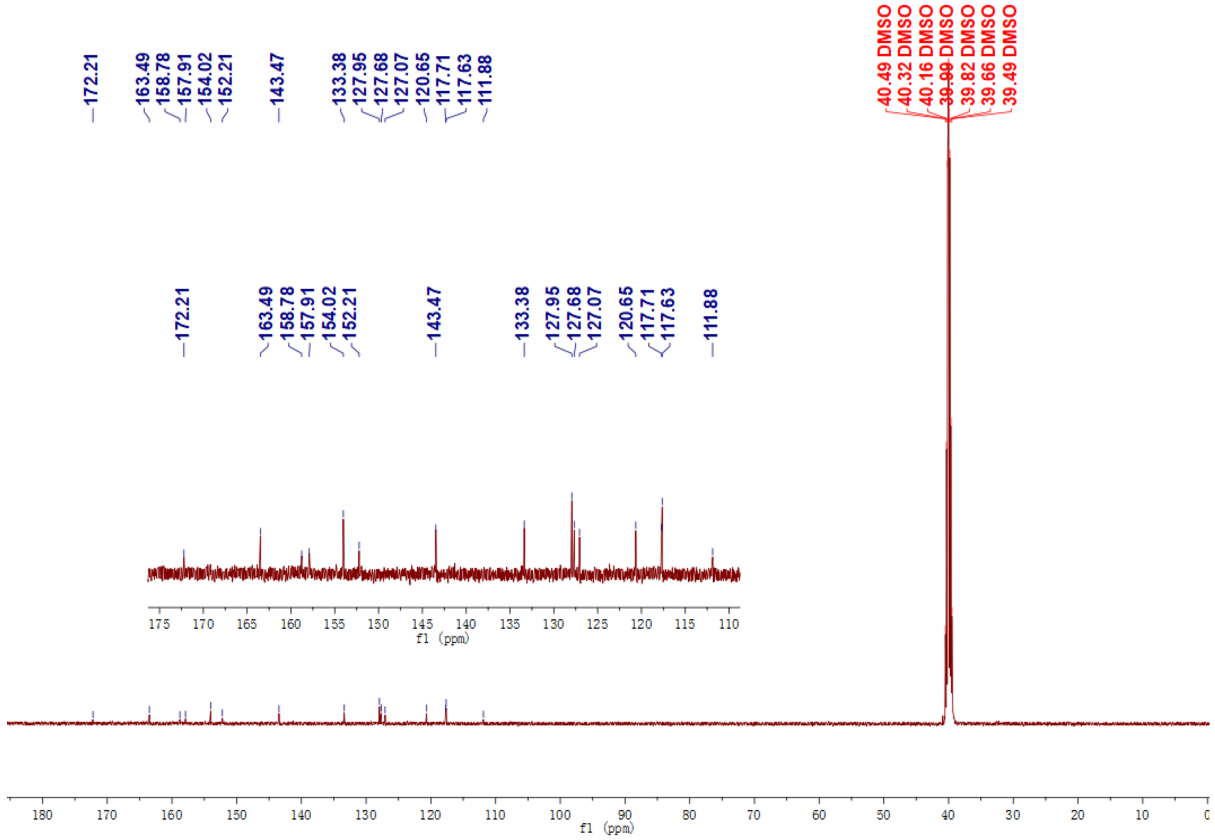

^{13}C NMR (125 MHz) of **17** in DMSO-d_6

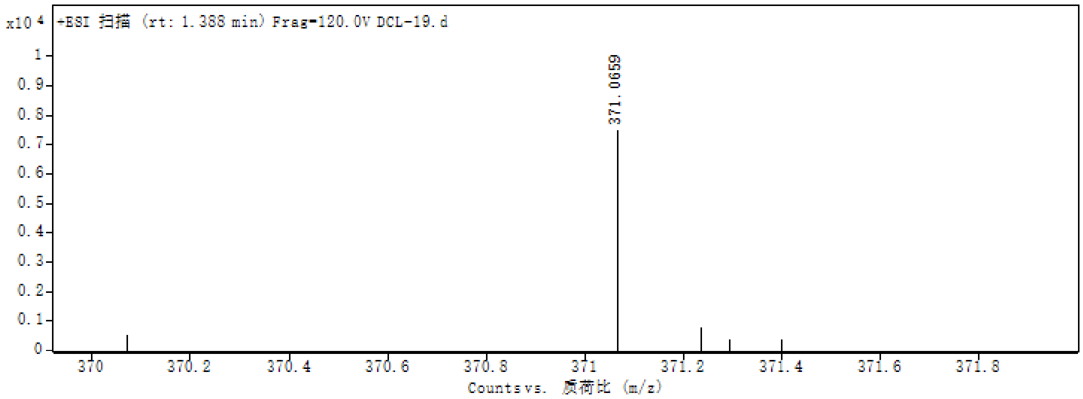

HR-MS of **17** in MeOH

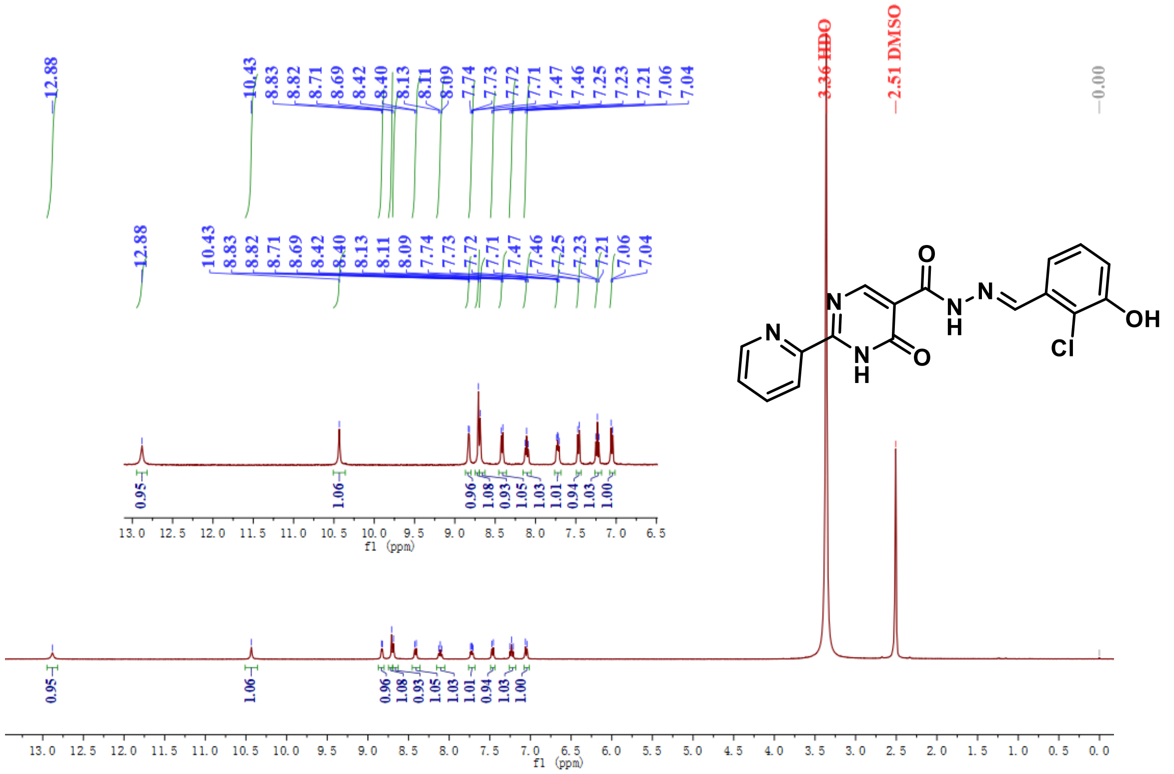

^1H NMR (400 MHz) of **18** in DMSO-d_6

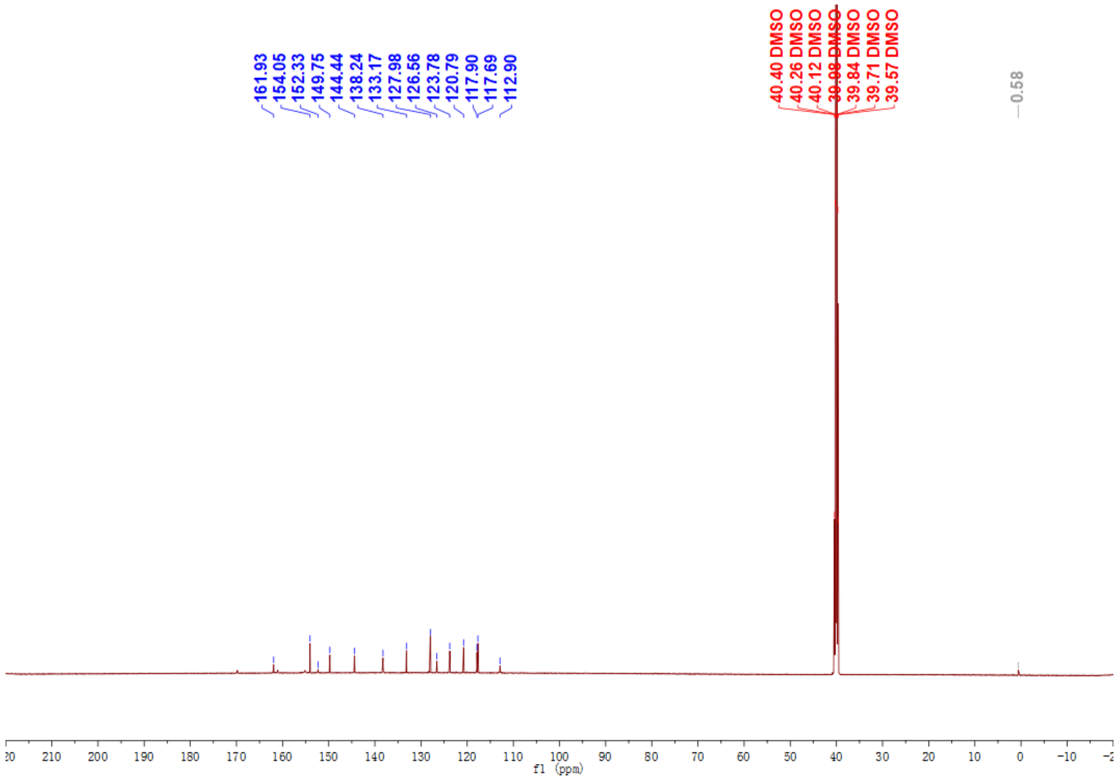

^{13}C NMR (125 MHz) of **18** in DMSO-d_6

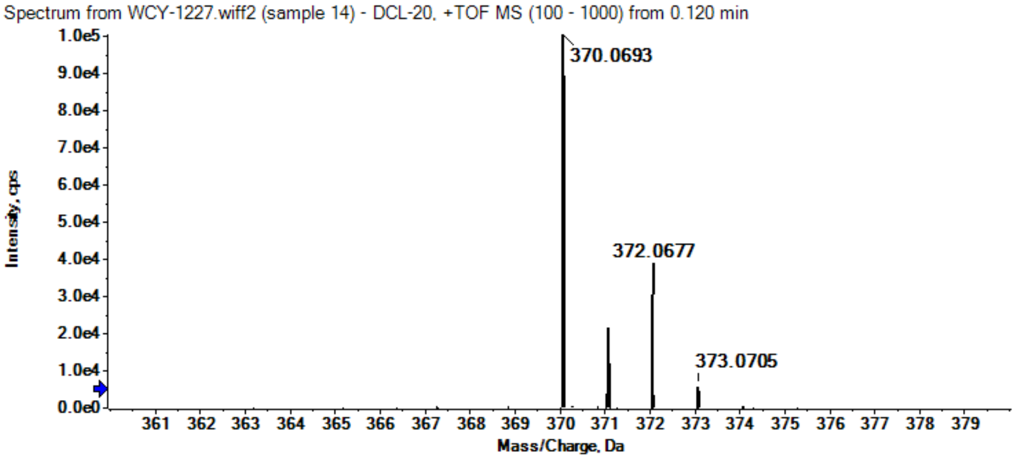

HR-MS of **18** in MeOH

References

[1] Z. Li, S. Zhen, K. Su, A. Tumber, Q. Yu, Y. Dong, M. McDonough, C. J. Schofield, X. Zhang, *Chem. Commun.* **2020**, *56*, 14199-14202.

[2] Y. R. Hong, H. T. Kim, S. Ro, J. M. Cho, S. H. Lee, I. S. Kim, Y. H. Jung, *Bioorg. Med. Chem. Lett.* **2014**, *24*, 3142-3145.

[3] X. Zhang, Y. Lei, T. Hu, Y. Wu, Z. Li, Z. Jiang, C. Yang, L. Zhang, Q. You, *J. Med. Chem.* **2020**, *63*, 10045-10060.

[4] a) Z. Li, S. Zhen, K. Su, A. Tumber, Q. Yu, Y. Dong, M. McDonough, C. J. Schofield, X. Zhang, *Chem. Commun.* **2020**, *56*, 14199-14202; b) Y. Lei, T, Hu, X. Wu, Y. Wu, Q. Bao, L. Shan, H. Xia, H. Sun, Q. You, X. Zhang, *ACS Med. Chem. Lett.* **2015**, *6*, 1236-1240.

[5] Z. Nikolovska-Coleska, R. Wang, X. Fang, H. Pan, Y. Tomita, P. Li, P. P. Roller, K. Krajewski, N. G. Saito, J. A. Stuckey, S. Wang, *Anal. Biochem.* **2004**, *332*, 261-273.

[6] T. L. Yeh, T. M. Leissing, M. I. Abboud, C. C. Thinnes, O. Atasoylu, J. P. Holt-Martyn, D. Zhang, A. Tumber, K. Lippl, C. T. Lohans, I. K. H. Leung, H. Morcrette, I. J. Clifton, T. D. W. Claridge, A. Kawamura, E. Flashman, X. Lu, P. J. Ratcliffe, R. Chowdhury, C. W. Pugh, C. J. Schofield, *Chem. Sci.* **2017**, *8*, 7651-7668.

[7] X. Yang, Y. Zhong, D. Wang, Z. Lu, *Anal. Methods.* **2021**, *13*, 5211-5215.

[8] Y. Wu, Z. Jiang, Z. Li, J. Gu, Q. You, X. Zhang, *J. Med. Chem.* **2018**, *61*, 5332-5349.

[9] X. Zhang, Y. Lei, T. Hu, Y. Wu, Z. Li, Z. Jiang, C. Yang, L. Zhang, Q. You, *J. Med. Chem.* **2020**, *63*, 10045-10060.

[10] L. Escrivá, G. Font, L. Manyes, *Food. Chem. Toxicol.* **2015**, *78*, 185-206.

[11] A. Canal-Martín, J. Sastre, M. J. Sánchez-Barrena, A. Canales, S. Baldominos, N. Pascual, L. Martínez-González, D. Molero, M. E. Fernández-Valle, E. Sáez, P. Blanco-Gabella, E. Gómez-Rubio, S. Martín-Santamaría, A. Sáiz, A. Mansilla, F. J. Cañada, J. Jiménez-Barbero, A. Martínez, R. Pérez-Fernández, *Nat. Commun.* **2019**, *10*, 2798.

Author contributions

Z.L. performed the ISISS assays, synthesis, and in vivo studies (with the help of Y.W. and L.Z.). Y.W. purified the PHD2 protein, synthesized the probe, and performed the FP assays and in vivo studies. S.Z., K.S., and F.Y. contributed to the synthesis of the inhibitors. M.A.M analysed the data from the ISISS assays. X.Z. and C.J.S. designed experiments and wrote the manuscript with the help from Z.L., Y.W., and M.A.M. All authors analysed the data.
